# Synthesis of Polysubstituted Pyridines and Pyrazines via Truce–Smiles Rearrangement of Amino Acid-Based 4-Nitrobenzenesulfonamides

**DOI:** 10.1021/acs.joc.2c03025

**Published:** 2023-02-16

**Authors:** Michaela Tkadlecová, Barbora Lemrová, Miroslav Soural

**Affiliations:** Department of Organic Chemistry, Faculty of Science, Palacký University, 17. listopadu 12, 771 46 Olomouc, Czech Republic

## Abstract

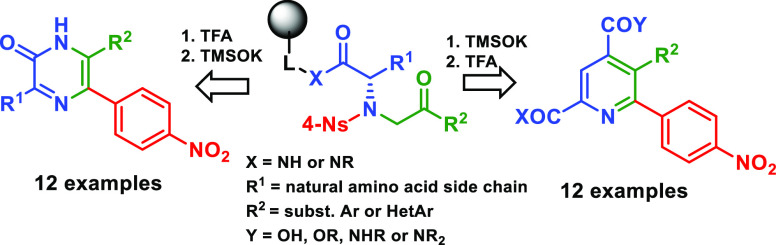

Immobilized l-glutamic acid β-methyl ester was sulfonylated with 4-nitrobenzenesulfonyl chloride and alkylated with various α-haloketones. The resulting sulfonamides were reacted with potassium trimethylsilanolate. Then, upon cleavage from the polymer support, tetrasubstituted pyridines were produced as the result of one-step C-arylation, aldol condensation, and oxidation. When cleavage from the resin occurred before the trimethylsilanolate treatment, C-arylation was followed by enamination, which yielded trisubstituted pyrazines. Through the developed protocols, targeted synthesis of novel heterocyclic derivatives was performed using mild reaction conditions and a number of readily available starting materials.

## Introduction

**Figure 1:**
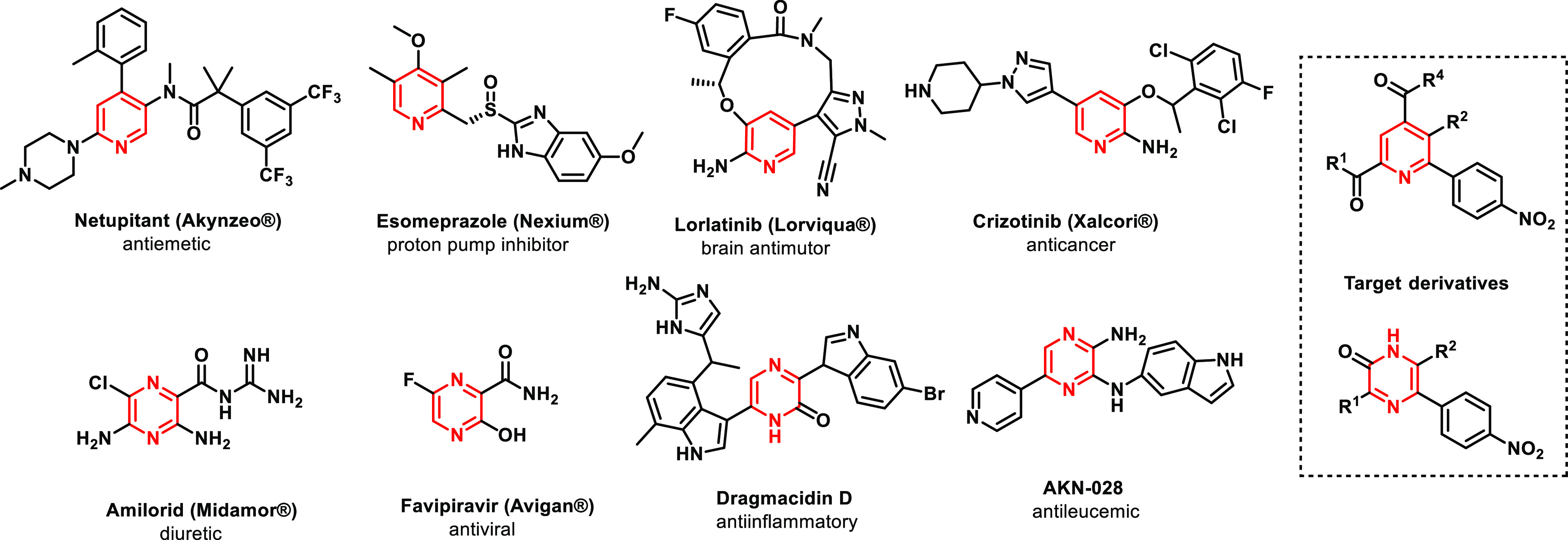
Examples of medicinally relevant, trisubstituted pyridines and pyrazines.

Pyridines are one of the most frequently studied heteroaromatics due to their occurrence in a vast number of biologically active compounds. They are known, for example, for their anti-inflammatory^1,2^ and antidepressant properties^3^ or as the reagents that modulate hypertension,^4^ hypotension,^5^ or HIV activity.^6^ In the list of FDA-approved drugs, the pyridine nucleus occupies the second position after piperazine among the nitrogen heterocycles (Figure 1).^7^

In addition to be useful as drugs, pyridines have been applied in materials,^8^ supramolecular structures,^9,10^ polymers,^11^ and organocatalysis.^12^ For all of these reasons, various synthetic approaches to substituted pyridines have been reported using either the cyclization of linear precursors^13−16^ or decoration of the pyridine nucleus.^17−19^ Although the latter approach is affected by the electron-withdrawing effect of the pyridine nitrogen atom, resulting in compromised electron density within the scaffold, recent progress in C–H activation and transition metal-catalyzed chemistry has considerably widened the synthetic possibilities. Despite this fact, the preparation of polysubstituted pyridines using cycloaddition or cyclocondensation methods still predominates. In this regard, a frequently utilized approach is based on multicomponent reactions (MCRs).^20^ Nevertheless, despite the efficacy of MCRs, harsh conditions are typically needed to proceed.

Different strategies leading to pyridines bearing diverse functionalities in certain positions have been introduced to date; however, some substitution patterns are still not available by known methods. This fact justifies the need for further development in the field. Recently, we reported a synthesis of pentasubstituted pyrroles related to the structure of atorvastatin using immobilized methyl aspartate as the starting material.^21^ In the following research, we attempted to use polymer-supported methyl glutamate to possibly receive tetrasubstituted pyridines. As in the previous case, we utilized the solid-phase synthesis (SPS). Although SPS was originally developed to comfortably prepare peptides and later oligonucleotides, the method has found its application also in the field of small molecules including heterocycles. The key advantage of SPS is that all reaction intermediates within the reaction sequence are quickly and simply isolated using filtration and wash with the fresh solvent; thus, parallel synthesis can be easily performed with only simple instrumentation and minimum hands-on time. Consequently, SPS has been frequently applied in pharmaceutical industry to produce collections of compounds for the high-throughput screening to accelerate the process of drug discovery and development.^22^

## Results and Discussion

**Scheme 1:**
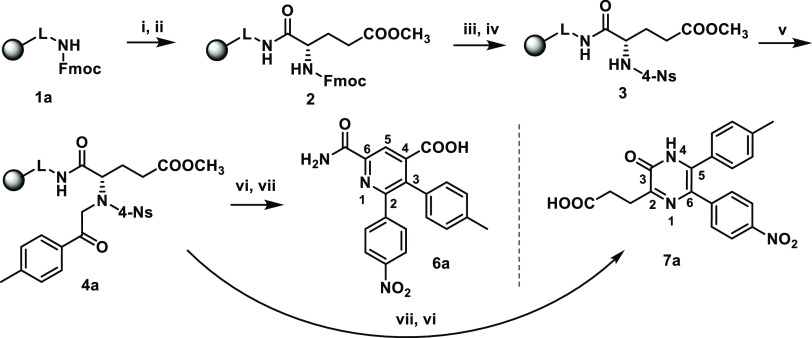
General Synthetic Pathway Reagents and conditions: (i) 50% piperidine in DMF, 20 min, rt; (ii) Fmoc-Glu(OMe)-OH, 1-hydroxybenzotriazole hydrate (HOBt), *N*,*N****′***-diisopropylcarbodiimide (DIC), DMF, dichloromethane (DCM), 16 h, rt; (iii) 10% piperidine in DMF, 20 min, rt; (iv) 4-NsCl, 2,6-lutidine, DCM, 16 h, rt; (v) haloketone, *N*,*N*-diisopropylethylamine (DIPEA), DMF, 16 h, rt; (vi) TMSOK, DMF, 30 min, rt; and (vii) 50% TFA in DCM, 1 h, rt.

Rink amide resin **1a** was acylated with Fmoc-Glu(OMe)-OH, followed by Fmoc cleavage and protection with 4-nitrobenzenesulfonyl chloride (4-NsCl) (Scheme 1). Sulfonamide **3** was alkylated with 2-bromo-1-(*p*-tolyl)ethan-1-one, and the resulting resin **4** was treated with 0.3 M potassium trimethylsilanolate (TMSOK) in dimethylformamide (DMF) as previously reported for the synthesis of pyrroles.^21^ The similar course of the reaction was observed, and after being cleaved from the polymer support with trifluoroacetic acid (TFA), the pyridine product **6a** was obtained in good crude purity (above 50%, calculated from UHPLC-UV traces) and overall yield (39%, calculated after the final purification from the loading of Rink amide resin).

**Scheme 2:**
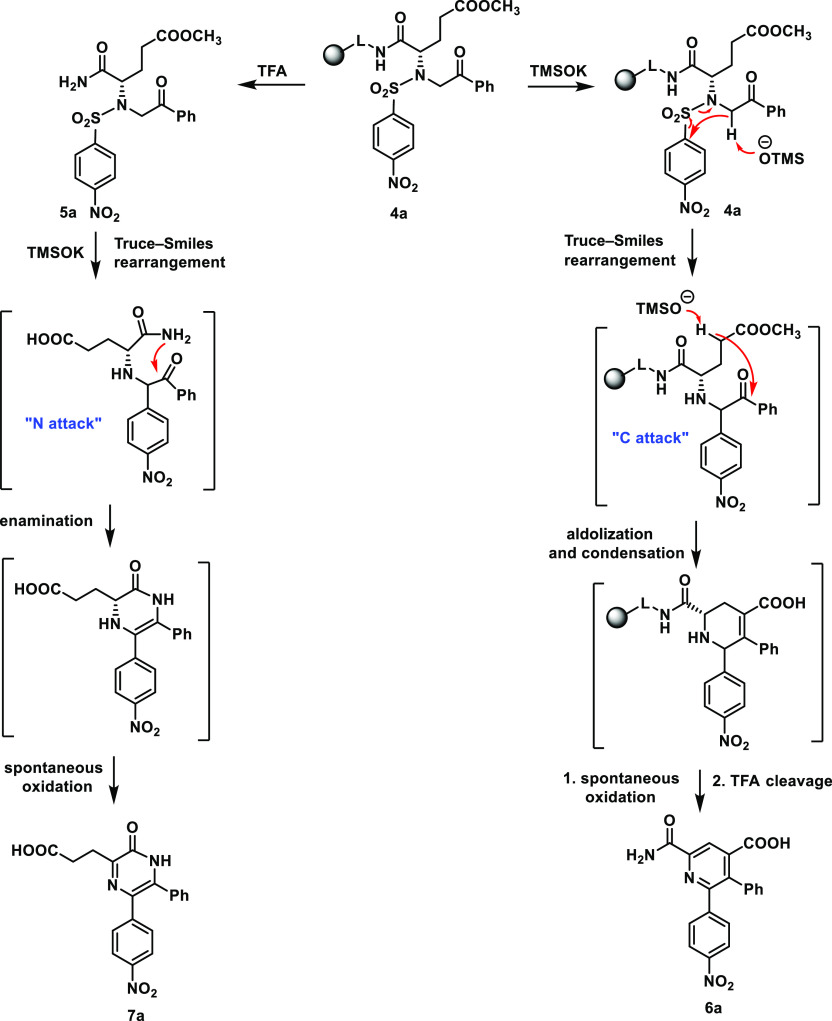
Simplified Mechanistic Description of the Pyridines or Pyrazines Formation

In contrast to the cyclization of pyrroles, the methylester was unstable toward TMSOK, and the desired pyridine was received as the free carboxylic acid, which considerably simplified further on-resin modifications (see later in the text). Interestingly, when resin **4a** was treated with TFA followed by exposure to TMSOK/DMF, a different course of reaction was observed, and the formation of pyrazine derivative **7a** was achieved as the result of subsequent C-arylation and enamination (Scheme 2).

This outcome indicated that the benzhydryl moiety of the Rink amide linker serves as the protecting group, which directs the ketone reaction toward the activated methylene group, resulting in pyridines; in the absence of benzhydryl, the nucleophilic attack of the amide nitrogen occurs, resulting in the formation of pyrazines. Pyrazine **7a** was obtained in high crude purity (79%, calculated from UHPLC-UV traces) and overall yield (34%, calculated after final purification using the loading of Rink amide resin). NMR characterization indicated that in solution, compound **7a** exclusively forms the lactim tautomer, as deduced from the absence of carbonyl signal in ^13^C NMR analysis. On the other hand, FT-IR analysis in the solid-state has proven the formation of lactam based on detection of vibrational bands for NH and CO functional groups (see the Experimental Section).

Next, we screened different bases that might be applicable to the synthesis of target compounds. Intermediate **4a** was exposed to different reagents (see Table 1).

**Table 1:** Bases Tested for the Conversion of **4a** into **6a**^a^

base	concn	solvent	crude purity (%)^b^
TMSOK	0.3	DMF	>50
TMSOK	0.03	DMF	<20
TMSOK	0.003	DMF	0 (starting material)
LDA	0.3	DMF	0 (mixture of unkn. cmpds)
BTTP	0.3	DMF	0 (mixture of unkn. cmpds)
NaH	0.3	DMF	0 (mixture of unkn. cmpds)
DBU	0.3	DMF	0 (mixture of unkn. cmpds)
DABCO	0.3	DMF	0 (mixture of unkn. cmpds)
TEA	0.3	DMF	0 (starting material)
TMSOK	0.3	THF	<10
TMSOK	0.3	DMSO	0 (mixture of unkn. cmpds)

a **4a** was shaken with a base for 30 min at room temperature, then the resin was washed with DMF and DCM, treated with 50% TFA/DCM for 30 min, the cleavage cocktail was evaporated, and the residual material was analyzed with UHPLC-MS.

b Crude purity after the entire reaction sequence calculated from UHPLC-UV traces.

Interestingly, only utilizing TMSOK led to the formation of product **6a**, whereas all other tested bases furnished either the starting material or mixtures of unknown compounds. This result proves that the base-specificity is very large and even higher than that of pyrrol cyclization, for which lithium diisopropylamide (LDA), *tert*-butylimino-tri(pyrrolidino)phosphorane (BTTP), and sodium hydride were also applicable.^21^ Additional experiments revealed that even 0.03 M TMSOK triggered the conversion (although incomplete), whereas lowering the concentration to 0.003 M was not applicable. Finally, the last two entries of Table 1 show that the TMSOK-induced cyclization is not only a base- but also solvent-dependent reaction.

**Scheme 3:**
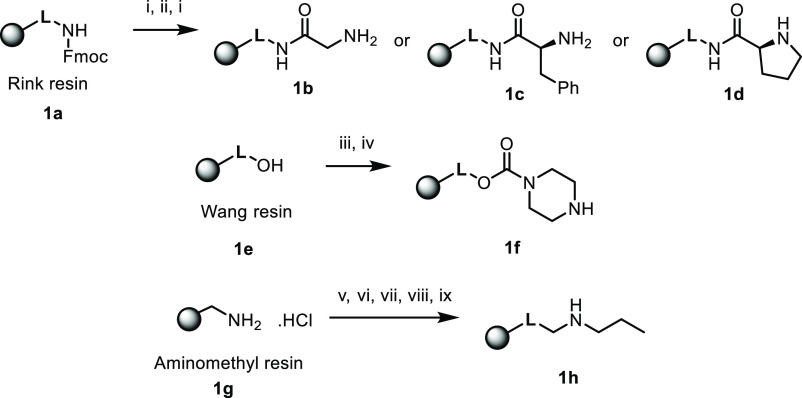
Synthesis of Different Starting Materials to Modify the C2 Position Reagents and conditions: (i) 50% piperidine in DMF; (ii) Fmoc-AA-OH, HOBt, DIC, DMF, DCM, 16 h, rt.; (iii) 1,1-carbonyldiimidazole (CDI), pyridine, DCM, 2 h, rt; (iv) piperazine, DCM, 16 h, rt; (v) 5% DIPEA, DCM, 5 min; (vi) 4-(4-formyl-3,5-dimethoxyphenoxy)butyric acid, HOBt, DIC, DCM/DMF (1:1), 16 h, rt; (vii) propylamine, 10% acetic acid, anhydrous DMF, 16 h, rt; (viii) sodium triacetoxyborohydride, 5% acetic acid, anhydrous DMF, 4 h, rt; and (ix) 20% piperidine in DMF, 10 min, rt.

With optimal conditions in hand, we began evaluating the limitations and scope using variously substituted starting materials. 2-Bromo-1-(4-fluorophenyl)ethan-1-one, 2-bromo-1-(4-methoxy-phenyl)ethan-1-one, 2-bromo-1-(3-methoxy-phenyl)ethan-1-one 2-bromo-1-(thiophen-3-yl)ethan-1-one, and 1-(4-amino-3,5-dichlorophenyl)-2-bromoethan-1-one yielded the desired pyridines **6** (Table 2), whereas alkylation with 1-chloropropane-2-one was unsuccessful under various conditions (data not shown). Next, we tested a possible variation in the position C2 (carboxamide). For this purpose, Rink amide resin was acylated with amino acids Fmoc-Gly-OH, Fmoc-Pro-OH, and Fmoc-Ala-OH, and Wang-piperazine resin **1e** was prepared as the polymer support to immobilize methyl glutamate. Furthermore, propylamine was immobilized as **1h** via reductive amination of an aminomethyl resin equipped with a benzaldehyde linker (Scheme 3). Starting resins **1d**, **f**, and **h** yielded the corresponding pyridines (Table 2), whereas the application of **1b** and **c** failed in the cyclization step.

**Table 2:**
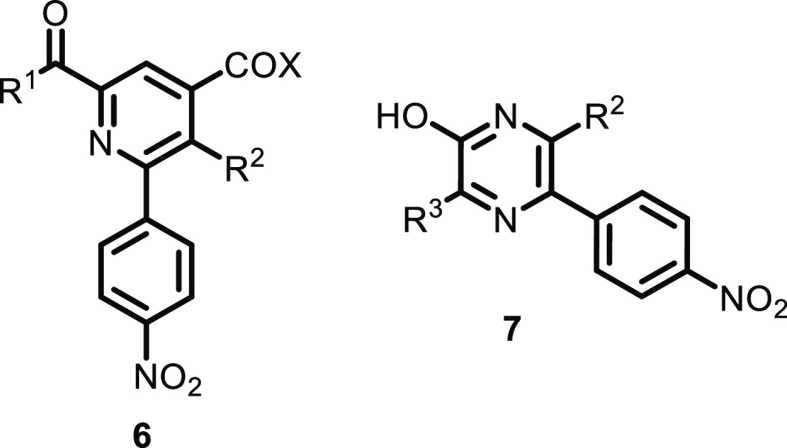

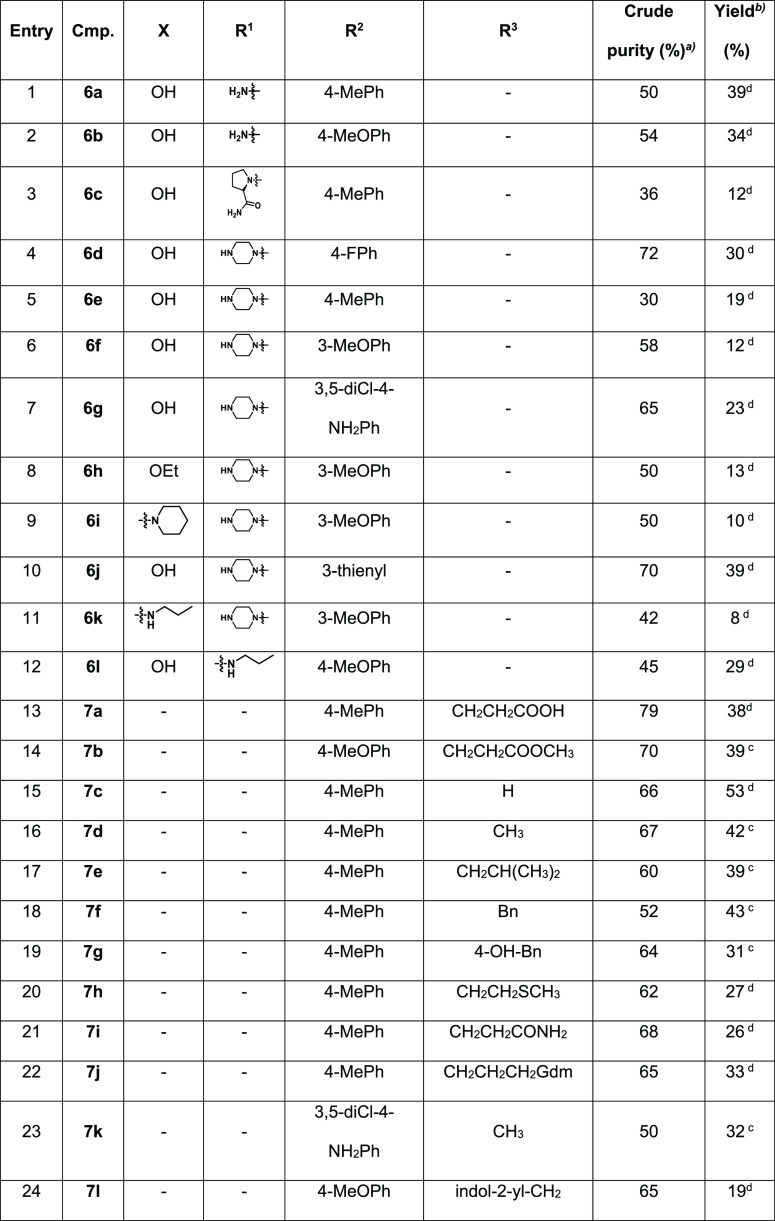
List of Synthesized and Fully Characterized Pyridines and Pyrazines

a Crude purity after the entire reaction sequence, as calculated from UHPLC-UV traces.

b Overall yield after the entire reaction sequence and final purification, as calculated from the loading of the starting resin.

c Purified via column chromatography on silica gel.

d Reversed-phase semipreparative HPLC purification.

**Figure 2:**
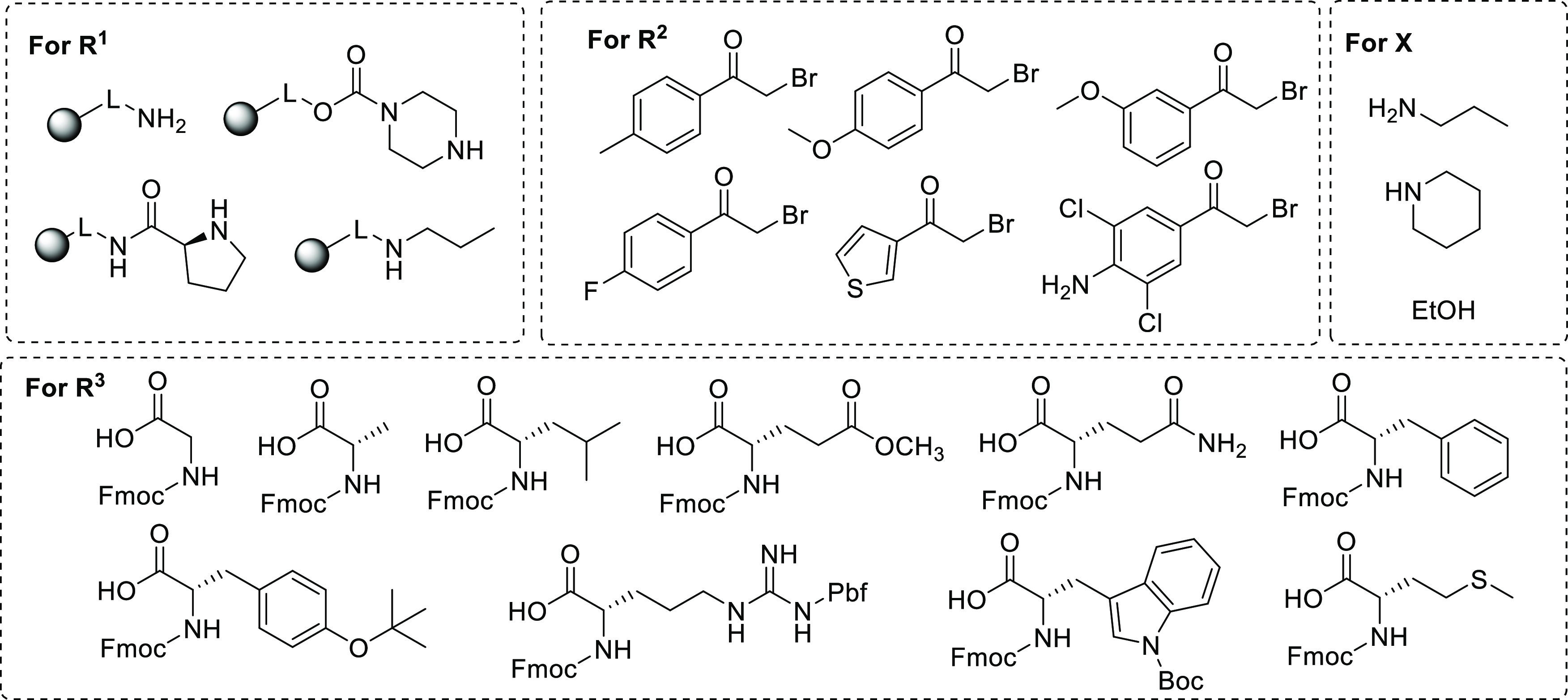
List of successfully tested building blocks.

**Scheme 4:**
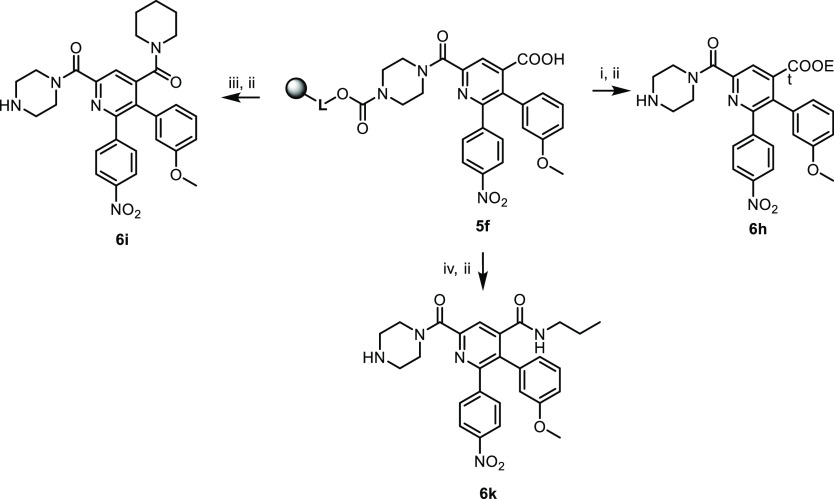
Further On-Resin Modification of **5f** Reagents and conditions: (i) HBTU, ethanol, DIPEA, DMAP, DMF, 16 h, rt; (ii) 50% TFA in DCM, 1 h, rt; (iii) HBTU, piperidine, DIPEA, DMF, 16 h, rt; and (iv) (a) *N*-hydroxysuccinimide, DIC, DMF, 2 h, rt, (b) 10% propylamine, DMF, 16 h, rt.

On-resin modification of the C4 position was also performed by converting immobilized carboxylic acid **5f** into its ethyl ester or preparing representative amides **6i** and **6k** using either *N*,*N*,*N*′,*N*′-tetramethyl-*O*-(1*H*-benzotriazol-1-yl)uronium hexafluorophosphate (HBTU) or *N*-hydroxysuccinimide as the activating agents (Scheme 4). Finally, the large variation in the substitution of pyrazines was proven to be accessible using different Fmoc-amino acids, such as Fmoc-Gly-OH, Fmoc-Ala-OH, Fmoc-Gln-OH, Fmoc-Met-OH, Fmoc-Phe-OH, Fmoc-Arg(Pbf)-OH, Fmoc-Tyr(*t*Bu)-OH, Fmoc-Leu-OH, and Fmoc-Trp(Boc)-OH (see Figure 2 for all tested building blocks). It is worth mentioning that Fmoc-Ser(*t*Bu)-OH was not applicable due to the formation of oxazines in the cyclization step, as reported previously.^23^

## Conclusions

We have developed a simple method to prepare tetrasubstituted pyridines with a substitution pattern that is not accessible by other synthetic approaches reported to date. With this method, the pyridine scaffold can be modified with symmetrical or nonsymmetrical carboxamides at positions C^2^ and C^4^ in combination with variously substituted aryl moieties at position 5. Furthermore, when reversing the last two steps of the reaction sequence, novel trisubstituted pyrazines can be obtained from immobilized α-amino acids as the starting materials. Compared to previously reported synthetic routes, this approach utilized only mild reaction conditions within the reaction sequence. In total, 24 representative products (pyrazines and pyridines) were synthesized and fully characterized. Nevertheless, with respect to the synthetic capacity of SPS and considering the number of readily available building blocks (amines, amino acids, haloketones), larger collections of target compounds can be rapidly prepared with the developed protocols to screen biological target(s) of choice, eventually using semi-automated or fully automated synthetic approaches.

## Experimental Section

### General Information

Solvents and chemicals were purchased from Sigma-Aldrich (Milwaukee, USA, www.sigmaaldrich.com), Acros Organic (Geel, Belgium, www.acros.com), and Fluorochem (Derbyshire, UK, www.fluorochem.co.uk). Rink Amide resin (100–200 mesh, 1% DVB, 0.6 mmol/g), Wang resin (100–200 mesh, 1% DVB, 1.4 mmol/g), and aminomethyl resin (100–200 mesh, 1% DVB, 0.98 mmol/g) were obtained from AAPPTec (Louisville, USA, www.aapptec.com). SPS was carried out in plastic reaction vessels (syringes, each equipped with a porous disk) using a manually operated synthesizer (Torviq, Niles, USA, www.torviq.com). Dry solvents were dried over 4 Å molecular sieves or stored as received from commercial suppliers.

All reactions were carried out at ambient temperature (23 °C) unless stated otherwise. For the LC/MS analyses, a sample of resin (∼5 mg) was treated with 50% TFA in DCM, the cleavage cocktail was evaporated under a stream of nitrogen, the cleaved compounds dissolved in CH_3_CN/H_2_O (20 or 50%; 1 mL) and the resin was removed by filtration. LC/MS analyses were carried out using a UHPLC-MS system consisting of UHPLC chromatograph Acquity with photodiode array detector and single quadrupole mass spectrometer (Waters) using C18 X-Select HSS T3 column (2.5 μm, 3.0 mm × 50 mm) at 30 °C and flow rate of 0.6 mL/min. Mobile phase (MP) was (A) 0.01 M ammonium acetate (AmAc) in H_2_O, and (B) CH_3_CN, linearly programmed gradient elution. The ESI source operated at capillary voltage of 3 kV, desolvation temperature of 350 °C and source temperature of 120 °C. Purification was carried out using semipreparative HPLC (Waters, USA) equipped with PDA and MS detector. All separations were done using C18 reverse phase column (YMC-Actus Pro C18 column, 20 × 100 mm, 5 μm particle size). The MP consists of the aqueous solution of 0.01 M ammonium acetate and HPLC gradient grade acetonitrile. A linear gradient was formed by acetonitrile in aqueous ammonium acetate in 6 min with flow rate 15 mL/min. In case of **7b**, **7d**, **7e**, **7f**, **7g**, and **7k**, the purification was performed by normal phase (silica gel chromatography). The precise purification method is specified for each product in its experimental procedure. Residual solvents (H_2_O and AmAc buffer) were lyophilized by the ScanVac Coolsafe 110–4 operating at −110 °C. HRMS analysis was performed using LC–MS (Dionex Ultimate 3000, Thermo Fischer Scientific, USA) with Exactive Plus Orbitrap high-resolution mass spectrometer (Thermo Exactive plus, Thermo Fischer Scientific, USA) operating at positive or negative full scan mode (120 000 fwmh) in the range of 100–1000 *m*/*z* with electrospray ionization operating at 150 °C and the source voltage of 3.6 kV. Chromatographic separation was performed on Phenomenex Gemini column (C18, 50 × 2 mm, 3 μm particle size) with isocratic elution and MP containing MeOH/H_2_O/formic acid 95:5:0.1. The samples were dissolved in CH_3_CN or MeOH/H_2_O (95:5 v/v). NMR experiments were performed with the use of ECX500 spectrometer (JEOL RESONANCE, Tokyo, Japan) at a magnetic field strength of 11.75 T corresponding to ^1^H and ^13^C resonance frequencies of 500.16 and 125.77 MHz at a temperature of 25 °C or ECA400II spectrometer (JEOL) at magnetic field strengths of 9.39 T with operating frequencies 399.78 MHz for ^1^H and 100.53 MHz for ^13^C at a temperature of 27 °C. In the case of **6d**, **6e**, **6f**, **6i**, and **6k**, the carbon measurements were performed at 120 °C. Deuterated TFA was used (^13^C-116.5 quartet) due to the poor solubility of derivative **6c** in DMSO-*d*_6_. Chemical shifts (δ) are reported in parts per million (ppm) and coupling constants (*J*) are reported in Hertz (Hz). The signals of DMSO-*d*_6_ and CDCl_3_ were set at 2.50 and 7.26 ppm in ^1^H NMR spectra and at 39.50, 77.00, and *d*-TFA 116.5 (quartet) ppm in ^13^C NMR spectra. Abbreviations in NMR spectra: br. s—broad singlet, s—singlet, d—doublet, dd—doublet of doublets, ddd—doublet of doublets of doublets, dt—doublet of triplets, t—triplet, and m—multiplet. IR spectra were recorded by DRIFT (diffuse reflectance infrared Fourier transform) on a Thermo Nicolet AVATAR 370 FTIR spectrometer. Absorbance peaks (wavenumbers) are reported in reciprocal centimeters (cm^–1^), and transmittances (*T*) are reported in percentages (%).

### General Method for Calculation of Yields Using ^1^H NMR

^1^H NMR spectra of external standard in DMSO-*d*_6_ at three different concentrations were recorded. In each spectrum, the solvent signal was integrated followed by the integration of selected H_Ar_ signals of the external standard. Ratios of solvent/standard signal areas along with a known quantity of the standard were used to construct a calibration curve. Then, ^1^H NMR spectra of the studied sample were measured, and the ratio of solvent/sample (selected H_Ar_ signal) areas was determined. Using the calibration curve, the quantity of compound in the sample was calculated.

### General Method for Calculation of Crude Purity

The UHPLC-UV data (see the parameters of instrumentation and used gradient above) was processed by the MassLynx Software version 4.1. The obtained spectrum (diode array 200–500 nm) was integrated using the method of internal normalization.

### Quantification of the Resin Loading

Quantification of loading of Rink or Wang resin with immobilized Fmoc-Glu(OMe)-OH, Fmoc-Ala-OH, Fmoc-Gly-OH, Fmoc-Pro-OH, Fmoc-Leu-OH, Fmoc-Gln-OH, Fmoc-Met-OH, Fmoc-Arg(Pbf)-OH, Fmoc-Phe-OH, Fmoc-Tyr(*t*Bu)-OH, and Fmoc-Trp(Boc)-OH: after immobilization, the sample of resin (∼30 mg) was washed with DCM (3×), MeOH (5×), dried, and divided into two samples (2 × 10 mg). Both samples were cleaved from the resin using TFA in DCM (0.5 mL, 50%; for immobilized Fmoc-Arg(Pbf)-OH 0.5 mL 90%) for 2 h at ambient temperature. The cleavage cocktail was evaporated by a stream of nitrogen and oily residue was extracted into 1 mL of MeOH and analyzed by UHPLC-UV-MS. Loading of resin was calculated with the use of an external standard (Fmoc-Ala-OH, 0.5 mg/mL).

### Quantification of Resin with Immobilized Piperazine and Propylamine

After immobilization, the resin (∼30 mg) was reacted with Fmoc-OSu (65 mg, 0.2 mmol) in DCM (0.5 mL) for 30 min at room temperature and subsequently washed with DCM (3×), with MeOH (5×), dried, and divided into two samples (2 × 10 mg). The quantification was carried out according to the procedure described in the previous paragraph.

## Experimental Procedures

### Immobilization of Fmoc-Amino Acids on Rink Amide Resin (**2**, **1b**, **1c**, **1d**)

Rink amide resin (1 g, 0.6 mmol/g) was washed with DCM (3×) and DMF (3×) and then subjected to Fmoc-deprotection using 50% solution of piperidine in DMF (10 mL) at room temperature. After shaking for 20 min, the resin was washed with DMF (5×). A solution of Fmoc-amino acid (2 mmol), HOBt (306 mg, 2.0 mmol), and DIC (312 μL, 2.0 mmol) in DMF/DCM (10 mL, 50%) was added and the reaction mixtures were shaken for 24 h at ambient temperature. After the reaction, the resins were washed with DMF (3×) and with DCM (3×). The quantification was carried out as described in the paragraph “Quantification of the Resin Loading”. The analytical sample of cleaved products (**2**, **1b**, **1c**, **1d**) was obtained as a white amorphous solid. Calculated loadings were 0.3 mmol/g.

### Immobilization of Piperazine to Wang Resin (**1f**)

Wang resin (1 g, 1.4 mmol/g) was washed with DCM (3×). A solution of CDI (810 mg, 5.0 mmol) and pyridine (400 μL, 5.0 mmol) in 10 mL of DCM was added and the resin slurry was shaken for 2 h at ambient temperature. The resin was then washed with DCM (3×) and a solution of piperazine (430 mg, 5.0 mmol) in 10 mL of DCM was added. The resin was shaken for 16 h and washed with DCM (5×). Sample of the resulting resin was derivatized with FmocOSu according to the procedure described in the paragraph “Quantification of the Resin Loading”. The analytical sample of cleaved product **1f** was obtained as a white amorphous solid. Calculated loading was 0.6 mmol/g.

### Immobilization of Propylamine by Reductive Amination on Aminomethylene Resin with Benzaldehyde Linker (**1h**)

Aminomethylene resin (200 mg, 0.98 mol/g) was washed with DCM (3×), neutralized with 5% DIPEA in DCM (2 mL) for 5 min, and washed with DCM (3×). A solution of 4-(4-formyl-3,5-dimethoxyphenoxy)butyric acid (214 mg, 0.8 mmol), HOBt (122 mg, 0.8 mmol), and DIC (122 μL, 0.8 mmol) in 2 mL of DCM/DMF (50%) was added to the resin and the suspension was shaken for 16 h. The resin was washed with DMF (3×), DCM (3×), and anhydrous DMF (3×). Propylamine (66 μL, 0.8 mmol) was dissolved in a 10% solution of AcOH (200 μL) in anhydrous DMF (1.8 mL). The resulting solution was added to the resin and the suspension was shaken for 16 h. Sodium triacetoxyborohydride (56.5 mg, 0.26 mmol) was dissolved in a 5% solution AcOH (50 μL) in anhydrous DMF (950 μL) and the solution was added to the resin. The syringe was pierced with a needle at the top and was placed in a horizontal position. Suspension was shaken for 1 h. A second part of sodium triacetoxyborohydride (56.5 mg) was added and the resin was shaken for 1 h. Then, the last part of sodium triacetoxyborohydride (56.5 mg) was added and the mixture was shaken for 2 h. The resin was washed with DMF (3×) and neutralized with 20% piperidine in DMF (2 mL) for 10 min. Finally, the resin was washed with DMF (3×) and DCM (3×). Sample of the resulting resin was derivatized with FmocOSu according to the procedure described in the paragraph “Quantification of the Resin Loading”. The analytical sample of cleaved product **1h** was obtained as a white amorphous solid. Calculated loading was 0.6 mmol/g.

### Nosylation with 4-Nitrobenzenesulfonyl Chloride (**3**)

Resin **2** (1 g) was treated with 20% piperidine in DMF (10 mL) for 20 min and subsequently washed with DMF (3×) and DCM (5×). A solution of 4-NsCl (640 mg; 3 mmol) and 2,6-lutidine (380 μL, 3 mmol) in DCM (10 mL) was added to the resin and the resin slurry was shaken for 16 h. The resin was washed with DCM (5×) and analyzed by the standard procedure. The analytical sample of cleaved products **3** was obtained as a light yellow amorphous solid.

### Alkylation with α-Haloketones (**4**)

Resin **3** (500 mg) was washed with DMF (3×) and a solution of α-haloketone (2.5 mmol) and DIPEA (870 μL, 5 mmol) in DMF (5 mL) was added. The reaction mixture was shaken for 16 h at ambient temperature. The resin was washed with DMF (3×) and with DCM (3×) and analyzed by the standard procedure. The analytical sample of cleaved products **4** was obtained as a brown oily residue.

### Pyridine Cyclization and Cleavage (**6**)

Resin **4** (500 mg which theoretically corresponds to 0.15 mmol for **4a–b**; 0.3 mmol for **4d–l**) was washed with DMF (3×), a solution of TMSOK (1 g; 0.3 mmol) in 5 mL of DMF was added and the slurry was shaken at room temperature for 30 min. The resin was washed with DMF (3×) and DCM (3×) and then analyzed. The resin (500 mg) was treated with 50% TFA in DCM (5 mL) for 1 h (90% TFA solution for 2 h was used for derivative **6a**, **6b**). The cleavage cocktail was separated, and the resin was washed with 50% TFA in DCM (2×). The washes were collected and evaporated by a stream of nitrogen. The crude product was purified by semipreparative RP-HPLC by method 20_50 CH_3_CN in AmAc for **6a–f**, method 20_40 CH_3_CN in AmAc for **6g** and **6j**, and method 25_50 CH_3_CN in AmAc for **6l**.

### Pyrazine Cyclization (**7**)

Resin **4** (500 mg which theoretically corresponds to 0.15 mmol) was treated with 50% TFA in DCM for 1 h (90% TFA solution for 2 h was used for derivative **7j**). The cleavage cocktail was separated and the resin was washed with 50% TFA in DCM (2×). The washes were collected and evaporated by a stream of nitrogen. The oily residue was dissolved in 2 mL of DMF followed by TMSOK addition to pH 12 at 0 °C, and the resulting solution was stirred at ambient temperature for 30 min. The cyclization of derivative **7b** was carried out at pH 10 to prevent the methyl ester group saponification. After the cyclization, DMF was lyophilized and the crude product was purified using silica gel chromatography with MP ethyl acetate (EA) for **7b** and **7g**, DCM/EA (1:1) for **7d**, **7f**, and **7k**, and hexane/EA (1:1) for **7e**, or by semipreparative RP-HPLC by method 30_60 CH_3_CN in AmAc for **7a**, **7i**, and **7j**, method 40_70 CH_3_CN in AmAc for **7c**, and method 50_80 CH_3_CN in AmAc for **7h** and **7l**.

### Esterification (**6h**)

The resin **5f** (500 mg which theoretically corresponds to 0.3 mmol) was washed with DCM (3×) and DMF (3×). A solution of HBTU (760 mg; 0.4 mmol), ethanol (117 μL; 0.4 mmol), DIPEA (350 μL; 0.4 mmol), and DMAP (61 mg; 0.1 mmol) in DMF (5 mL) was added to the resin and the suspension was shaken at room temperature for 16 h. The resin was washed with DMF (3×) and DCM (3×). The compound was cleaved from the resin using the standard procedure and purified with RP-HPLC by method 30_60 CH_3_CN in AmAc.

### Amidation Using the HBTU Technique (**6i**)

The resin **5f** (500 mg which theoretically corresponds to 0.3 mmol) was washed with DCM (3×) and DMF (3×). A solution of HBTU (760 mg; 0.4 mmol), piperidine (200 μL; 0.4 mmol), and DIPEA (350 μL; 0.4 mmol) in DMF (5 mL) was added to the resin and the suspension was shaken at room temperature for 16 h. The resin was washed with DMF (3×) and DCM (3×). The compound was cleaved from the resin using the standard procedure and purified with RP-HPLC by method 30_60 CH_3_CN in AmAc.

### Amidation via an Activated Ester Using *N*-Hydroxysuccinimide (**6k**)

The resin **5f** (500 mg which theoretically corresponds to 0.3 mmol) was washed with DCM (3×) and DMF (3×). A solution of *N*-hydroxysuccinimide (230 mg; 0.4 mmol) and DIC (155 μL; 0.2 mmol) in DMF (5 mL) was added to the resin and the suspension was shaken at room temperature for 2 h. The resin was washed with DMF (5×), a 10% solution of propylamine in DMF (5 mL) was added and the resin was shaken for 16 h. The resin was washed with DMF (3×) and DCM (3×). The compound was cleaved from the resin using the standard procedure and purified with RP-HPLC by method 25_50 CH_3_CN in AmAc.

#### 6-Carbamoyl-2-(4-nitrophenyl)-3-(*p*-tolyl)isonicotinic Acid (**6a**)

Yellow solid, 5 mg (39%). ^1^H NMR (500 MHz, DMSO-*d*_6_): δ = 8.07–8.03 (m, 3H), 7.90 (s, 1H), 7.67 (s, 1H), 7.58–7.54 (m, 2H), 7.05–7.02 (m, 4H), 2.26 (s, 3H) ppm. ^13^C{^1^H} NMR (126 MHz, DMSO-*d*_6_): δ = 168.0, 165.2, 154.7, 149.2, 146.7, 146.0, 137.0, 134.7, 133.3, 131.3, 129.6, 128.6, 122.6, 119.0, 20.7 ppm. IR (DRIFT): ν_max_ = 3427; 3407 (N–H, O–H); 1727 (C=O); 1666 (C=O); 1514 (N–O); 1345 (N–O); 1314 (C=N) cm^–1^. HRMS (ESI-Orbitrap) *m*/*z*: [M + H]^+^ calcd for C_20_H_16_N_3_O_5_^+^ 378.1084; found, 378.1081 and [M – H]^−^ calcd for C_20_H_14_N_3_O_5_^–^ 376.0928; found, 376.0938.

#### 6-Carbamoyl-3-(4-methoxyphenyl)-2-(4-nitrophenyl)isonicotinic Acid (**6b**)

Yellow solid, 6.6 mg (34%). ^1^H NMR (500 MHz, DMSO-*d*_6_): δ = 8.08–8.05 (m, 2H), 8.03 (br s, 1H), 7.86 (s, 1H), 7.65 (br s, 1H), 7.59–7.54 (m, 2H), 7.11–7.05 (m, 2H), 6.82–6.76 (m, 2H), 3.72 (s, 3H) ppm. ^13^C{^1^H} NMR (126 MHz, DMSO-*d*_6_): δ = 167.9, 165.2, 158.9, 154.9, 149.2, 146.7, 145.9, 134.8, 131.3, 131.0, 128.1, 122.6, 119.1, 113.6, 55.0 ppm. IR (DRIFT): ν_max_ = 3398 (N–H); 1674 (C=O); 1607 (C=O); 1516 (N–O); 1349 (N–O); 1293 (C=N); 1203 (C–O); 1025 (C–O) cm^–1^. HRMS (ESI-Orbitrap) *m*/*z*: [M + H]^+^ calcd for C_20_H_16_N_3_O_6_^+^ 394.1034; found, 394.1033 and [M – H]^−^ calcd for C_20_H_14_N_3_O_6_^–^ 392.0877; found, 392.0889.

#### 6-(3-Carbamoylpyrrolidine-1-carbonyl)-2-(4-nitrophenyl)-3-(*p*-tolyl)isonicotinic Acid (**6c**)

Yellow-brown oily substance, 8.9 mg (12%), received as the mixture of rotamers.^21^^1^H NMR (400 MHz, DMSO-*d*_6_): δ = 8.12–8.03 (m, 2H), 7.89 and 7.88 (two singlets for C–H, 1H), 7.54–7.46 (m, 2H), 7.42 and 7.31 (two singlets for N–H, 1H), 7.12–6.98 (m, 4H), 6.95 and 6.85 (two singlets for N–H, 1H), 5.07 and 4.45 (two doublet of doublets for C–H, *J* = 4.1 Hz; 4.2 Hz, 1H), 3.92–3.82 (m, 1H), 3.78–3.50 (m, 2H), 2.28 and 2.27 (two singlets for CH_3_, 3H), 2.24–2.05 (m, 1H), 2.01–1.90 (m, 1H), 1.89–1.78 (m, 2H) ppm. Measured at 120 °C ^1^H NMR (500 MHz, DMSO-*d*_6_): δ = 8.05–8.01 (m, 2H), 7.91 (s, 1H), 7.54–7.45 (m, 2H), 7.13–7.00 (m, 4H), 6.69 (br s, 2H), 3.89–3.71 (m, 2H), 2.29 (s, 3H), 2.25–2.13 (m, 1H), 2.06–1.92 (m, 2H), 1.93–1.81 (m, 2H) ppm. ^13^C{^1^H} NMR (101 MHz, DMSO-*d*_6_): δ = 173.4, 173.3, 168.0, 165.0, 164.4, 154.3, 153.9, 152.6, 152.4, 146.6, 146.5, 146.3, 146.1, 137.0, 133.5, 133.4, 133.2, 131.1, 131.0, 129.6, 128.7, 122.8, 122.7, 121.0, 120.9, 61.5, 61.3, 60.7, 49.5, 47.9, 32.4, 29.2, 24.9, 21.8, 20.8 ppm. IR (DRIFT): ν_max_ = 3394 (O–H); 3170 (N–H); 1674 (C=O); 1668 (C=O); 1622 (C=O); 1601 (C=O); 1517 (N–O); 1345 (N–O); 1314 (C=N) cm^–1^. HRMS (ESI-Orbitrap) *m*/*z*: [M + H]^+^ calcd for C_25_H_23_N_4_O_6_^+^ 475.1612; found, 475.1611 and [M – H]^−^ calcd for C_25_H_21_N_4_O_6_^–^ 473.1456; found, 473.1467.

#### 3-(4-Fluorophenyl)-2-(4-nitrophenyl)-6-(piperazine-1-carbonyl)isonicotinic Acid (**6d**)

Yellow solid, 44.6 mg (30%). ^1^H NMR (500 MHz, DMSO-*d*_6_): δ = 8.10–8.07 (m, 2H), 7.55 (s, 1H), 7.46–7.43 (m, 2H), 7.20–7.16 (m, 2H), 7.08–7.03 (m, 2H), 3.79–3.69 (m, 4H), 3.13–2.96 (m, 4H) ppm. ^13^C{^1^H} NMR (126 MHz, TFA-*d*): δ = 170.0, 166.2 (d, ^1^*J*_*C*-*F*_ = 236.8 Hz, 1C), 165.2, 158.9, 150.9, 148.5, 147.5, 142.8, 140.4, 133.2 (d, ^3^*J*_*C*-*F*_ = 9.0 Hz, 1C), 133.1, 129.6, 126.5, 125.8, 118.2 (d, ^2^*J*_*C*-*F*_ = 22.6 Hz, 1C), 46.3, 46.0, 41.9 ppm. IR (DRIFT): ν_max_ = 3598 (O–H); 1646 (C=O); 1593 (C=O); 1511 (N–O); 1346 (N–O); 1304 (C=N); 1221 (C–F) cm^–1^. HRMS (ESI-Orbitrap) *m*/*z*: [M + H]^+^ calcd for C_23_H_20_FN_4_O_5_^+^ 451.1412; found, 451.1411 and [M – H]^−^ calcd for C_23_H_18_FN_4_O_5_^–^ 449.1256; found, 449.1266.

#### 2-(4-Nitrophenyl)-6-(piperazine-1-carbonyl)-3-(*p*-tolyl)isonicotinic Acid (**6e**)

Yellow solid, 23.2 mg (19%). ^1^H NMR (400 MHz, DMSO-*d*_6_): δ = 8.10–7.99 (m, 2H), 7.54 (s, 1H), 7.49–7.40 (m, 2H), 7.07–6.95 (m, 4H), 3.73–3.63 (m, 4H), 3.04–2.91 (m, 4H), 2.18 (s, 3H) ppm. ^13^C{^1^H} NMR (126 MHz, DMSO-*d*_6_): δ = 167.3, 165.4, 153.9, 151.8, 146.4, 145.9, 136.3, 132.9, 132.6, 130.2, 129.3, 127.7, 121.8, 119.9, 43.8, 19.9 ppm. IR (DRIFT): ν_max_ = 3643 (O–H); 1632 (C=O); 1599 (C=O); 1517 (N–O); 1344 (N–O); 1302 (C=N) cm^–1^. HRMS (ESI-Orbitrap) *m*/*z*: [M + H]^+^ calcd for C_24_H_23_N_4_O_5_^+^ 447.1663; found, 447.1658 and [M – H]^−^ calcd for C_24_H_21_N_4_O_5_^–^ 445.1506; found, 445.1518.

#### 3-(3-Methoxyphenyl)-2-(4-nitrophenyl)-6-(piperazine-1-carbonyl)isonicotinic Acid (**6f**)

Yellow solid, 20.5 mg (12%). ^1^H NMR (500 MHz, DMSO-*d*_6_): δ = 8.09–8.04 (m, 2H), 7.58 (s, 1H), 7.50–7.45 (m, 2H), 7.08 (t, *J* = 7.9 Hz, 1H), 6.80–6.76 (m, 1H), 6.76–6.72 (m, 1H), 6.68 (d, *J* = 7.7 Hz, 1H), 3.77–3.66 (m, 4H), 3.57 (s, 3H), 3.06–2.95 (m, 4H) ppm. ^13^C{^1^H} NMR (126 MHz, DMSO-*d*_6_): δ = 169.9, 166.1, 158.5, 153.8, 151.5, 146.8, 146.5, 138.4, 131.9, 130.9, 128.8, 122.6, 122.4, 120.0, 116.0, 112.6, 54.8, 44.2, 43.0, 42.6 ppm. IR (DRIFT): ν_max_ = 3430 (N–H); 1640 (C=O); 1588 (C=O); 1506 (N–O); 1343 (N–O); 1304 (C=N); 1217 (C–O); 1013 (C–O) cm^–1^. HRMS (ESI-Orbitrap) *m*/*z*: [M + H]^+^ calcd for C_24_H_23_N_4_O_6_^+^ 463.1612; found, 463.1611 and for [M – H]^−^ calcd for C_24_H_21_N_4_O_6_^–^ 461.1456; found, 461.1467.

#### 3-(4-Amino-3,5-dichlorophenyl)-2-(4-nitrophenyl)-6-(piperazine-1-carbonyl)isonicotinic Acid (**6g**)

Yellow-orange solid, 40.1 mg (23%). ^1^H NMR (500 MHz, DMSO-*d*_6_): δ = 8.16–8.12 (m, 2H), 7.54–7.51 (m, 2H), 7.51 (s, 1H), 7.01 (s, 2H), 5.52 (s, 2H), 3.80–3.67 (m, 4H), 3.10–2.97 (m, 4H) ppm. ^13^C{^1^H} NMR (126 MHz, DMSO-*d*_6_): δ = 165.5, 163.9, 153.9, 151.8, 148.9, 146.5, 146.0, 139.6, 130.3, 129.0, 125.3, 121.9, 119.9, 117.2, 43.9 ppm. IR (DRIFT): ν_max_ = 3396 (N–H); 1630 (C=O); 1602 (C=O); 1522 (N–O); 1347 (N–O); 1302 (C=N); 734 (C–Cl); 688 (C–Cl) cm^–1^. HRMS (ESI-Orbitrap) *m*/*z*: [M + H]^+^ calcd for C_23_H_20_Cl_2_N_5_O_5_^+^ 516.0836; found, 516.0839 and [M – H]^−^ calcd for C_23_H_18_Cl_2_N_5_O_5_^–^ 514.0680; found, 514.0692.

#### Ethyl 3-(3-Methoxyphenyl)-2-(4-nitrophenyl)-6-(piperazine-1-carbonyl)isonicotinate (**6h**)

Yellow solid, 18.1 mg (13%). ^1^H NMR (500 MHz, DMSO-*d*_6_): δ = 8.13–8.08 (m, 2H), 7.88 (s, 1H), 7.56–7.51 (m, 2H), 7.24–7.18 (m, 1H), 6.91–6.86 (ddd, *J* = 8.3, 2.5, 0.5 Hz, 1H), 6.77–6.73 (m, 1H), 6.71–6.68 (m, 1H), 4.06 (q, *J* = 7.1 Hz, 2H), 3.64 (s, 3H), 3.52–3.47 (m, 4H), 2.84–2.72 (m, 4H), 0.94 (t, *J* = 7.1 Hz, 3H) ppm. ^13^C{^1^H} NMR (126 MHz, DMSO-*d*_6_): δ = 166.0, 165.3, 158.8, 154.8, 153.3, 146.8, 145.6, 142.4, 137.0, 133.6, 131.0, 129.3, 122.7, 122.0, 120.9, 115.3, 113.7, 61.5, 55.1, 47.8, 45.7, 45.1, 42.6, 13.4 ppm. IR (DRIFT): ν_max_ = 3425 (N–H); 1729 (C=O); 1632 (C=O); 1519 (N–O); 1347 (N–O); 1288 (C=N); 1200 (C–O); 1025 (C–O); 1008 (C–O) cm^–1^. HRMS (ESI-Orbitrap) *m*/*z*: [M + H]^+^ calcd for C_26_H_27_N_4_O_6_^+^ 491.1925; found, 491.1924.

#### [3-(3-Methoxyphenyl)-2-(4-nitrophenyl)-6-(piperazine-1-carbonyl)pyridin-4-yl](piperidin-1-yl)methanone (**6i**)

Yellow solid, 15.2 mg (10%). ^1^H NMR (500 MHz, DMSO-*d*_6_): δ = 8.11–8.06 (m, 2H), 7.60 (s, 1H), 7.59–7.55 (m, 2H), 7.20 (t, *J* = 7.9 Hz, 1H), 6.90–6.86 (m, 1H), 6.82 (s, 1H), 6.74 (d, *J* = 7.3 Hz, 1H), 3.76–3.66 (m, 4H), 3.65 (s, 3H), 3.41–3.33 (m, 4H), 2.98–2.89 (m, 4H), 1.45–1.31 (m, 3H), 1.32–1.04 (m, 2H), 0.96–0.78 (m, 1H) ppm. ^13^C{^1^H} NMR (126 MHz, DMSO-*d*_6_): δ = 165.1, 164.6, 158.6, 153.8, 152.4, 146.7, 146.5, 145.4, 136.0, 131.7, 130.3, 128.5, 122.0, 121.9, 119.1, 115.5, 113.7, 54.7, 46.4, 44.4, 41.0, 24.6, 24.1, 22.9 ppm. IR (DRIFT): ν_max_ = 1627 (C=O); 1600 (C=O); 1519 (N–O); 1346 (N–O); 1316 (C=N); 1287 (C–O); 1016 (C–O) cm^–1^. HRMS (ESI-Orbitrap) *m*/*z*: [M + H]^+^ calcd for C_29_H_32_N_5_O_5_^+^ 530.2398; found, 530.2399.

#### 2-(4-Nitrophenyl)-6-(piperazine-1-carbonyl)-3-(thiophen-3-yl)isonicotinic Acid (**6j**)

Yellow-orange solid, 46.6 mg (39%). ^1^H NMR (500 MHz, DMSO-*d*_6_): δ = 9.53 (br s, 1H), 8.12–8.07 (m, 2H), 7.53 (s, 1H), 7.51–7.46 (m, 2H), 7.35 (dd, *J* = 4.9, 3.0 Hz, 1H), 7.31 (dd, *J* = 2.9, 1.1 Hz, 1H), 6.80 (dd, *J* = 4.9, 1.0 Hz, 1H), 3.80–3.67 (m, 4H), 3.10–2.96 (m, 4H) ppm. ^13^C{^1^H} NMR (126 MHz, DMSO-*d*_6_): δ = 167.5, 165.4, 154.1, 151.9, 148.0, 146.6, 146.0, 135.6, 129.9, 129.0, 127.6, 125.0, 124.5, 121.9, 119.7, 44.0 ppm. IR (DRIFT): ν_max_ = 3410 (N–H); 1628 (C=O); 1582 (C=O); 1516 (N–O); 1344 (N–O); 1316 (C=N) cm^–1^. HRMS (ESI-Orbitrap) *m*/*z*: [M + H]^+^ calcd for C_21_H_19_N_4_O_5_S^+^ 439.1071; found, 439.1072 and [M – H]^−^ calcd for C_21_H_17_N_4_O_5_S^–^ 437.0914; found, 437.0924.

#### 3-(3-Methoxyphenyl)-2-(4-nitrophenyl)-6-(piperazine-1-carbonyl)-*N*-propylisonicotinamide (**6k**)

Yellow solid, 10.9 mg (8%). ^1^H NMR (500 MHz, DMSO-*d*_6_): δ = 9.29 (br s, 1H), 8.44 (t, *J* = 5.8 Hz, 1H), 8.13–8.08 (m, 2H), 7.71 (s, 1H), 7.54–7.48 (m, 2H), 7.21–7.15 (m, 1H), 6.88–6.84 (m, 1H), 6.79–6.76 (m, 1H), 6.70–6.67 (m, 1H), 3.94–3.83 (m, 4H), 3.64 (s, 3H), 3.24–3.15 (m, 4H), 3.02–2.95 (m, 2H), 1.27–1.20 (m, 2H), 0.68 (t, *J* = 7.4 Hz, 3H) ppm. ^13^C{^1^H} NMR (126 MHz, DMSO-*d*_6_): δ = 166.0, 165.6, 158.7, 154.0, 151.8, 148.0, 146.8, 145.8, 136.6, 133.4, 130.8, 129.2, 122.8, 122.2, 120.9, 115.7, 113.5, 55.0, 43.7, 42.8, 42.4, 21.9, 11.2 ppm. IR (DRIFT): ν_max_ = 3419 (N–H); 1644 (C=O); 1601 (C=O); 1518 (N–O); 1346 (N–O); 1287 (C=N); 1219 (C–O); 1024 (C–O) cm^–1^. HRMS (ESI-Orbitrap) *m*/*z*: [M + H]^+^ calcd for C_27_H_30_N_5_O_5_^+^ 504.2241; found, 504.2241 and [M – H]^−^ calcd for C_27_H_28_N_5_O_5_^–^ 502.2085; found, 502.2100.

#### 3-(4-Methoxyphenyl)-2-(4-nitrophenyl)-6-(propylcarbamoyl)isonicotinic Acid (**6l**)

Yellow solid, 23 mg (29%). ^1^H NMR (500 MHz, DMSO-*d*_6_): δ = 8.78 (t, *J* = 6.2 Hz, 1H), 8.16 (s, 1H), 8.13–8.09 (m, 2H), 7.62–7.58 (m, 2H), 7.09–7.05 (m, 2H), 6.88–6.84 (m, 2H), 3.73 (s, 3H), 3.31 (2H, overlay with water), 1.60–1.53 (m, 2H), 0.88 (t, *J* = 7.4 Hz, 3H) ppm. ^13^C{^1^H} NMR (126 MHz, DMSO-*d*_6_): δ = 169.0, 163.5, 158.5, 154.2, 148.5, 146.8, 146.5, 131.25, 131.18, 129.0, 128.3, 122.6, 118.5, 113.3, 112.6, 54.9, 40.6, 22.5, 11.3 ppm. IR (DRIFT): ν_max_ = 3377 (N–H); 1675 (C=O); 1602 (C=O); 1514 (N–O); 1348 (N–O); 1311 (C=N); 1254 (C–O); 1019 (C–O) cm^–1^. HRMS (ESI-Orbitrap) *m*/*z*: [M + H]^+^ calcd for C_23_H_22_N_3_O_6_^+^ 436.1503; found, 436.1501 and [M – H]^−^ calcd for C_23_H_20_N_3_O_6_^–^ 434.1347; found, 434.1357.

#### 3-[6-(4-Nitrophenyl)-3-oxo-5-(*p*-tolyl)-3,4-dihydropyrazin-2-yl]propanoic Acid (**7a**)

Yellow solid, 19.6 mg (38%). ^1^H-NMR (500 MHz, DMSO-*d*_6_): δ = 8.05 (d, *J* = 8.9 Hz, 2H), 7.43 (d, *J* = 9.0 Hz, 2H), 7.23–7.19 (m, 4H), 2.99 (t, *J* = 6.9 Hz, 2H), 2.68 (t, *J* = 6.9 Hz, 2H), 2.33 (s, 3H). ^13^C{^1^H} NMR (126 MHz, DMSO-*d*_6_): δ = 174.3, 156.5, 153.3, 145.6, 145.2, 139.5, 139.0, 130.8, 129.8, 129.5, 129.2, 129.0, 122.9, 30.6, 27.5, 20.9 ppm. IR (DRIFT): ν_max_ = 3438 (O–H); 1639 (2x C=O); 1591 (N–H); 1511 (N–O); 1337 (N–O); 1310 (C=N) cm^–1^. HRMS (ESI-Orbitrap) *m*/*z*: [M + H]^+^ calcd for C_20_H_18_N_3_O_5_^+^ 380.1241; found, 380.1239 and [M – H]^−^ calcd for C_20_H_16_N_3_O_5_^–^ 378.1084; found, 378.1090.

#### Methyl 3-[5-(4-Methoxyphenyl)-6-(4-nitrophenyl)-3-oxo-3,4-dihydropyrazin-2-yl]propanoate (**7b**)

Yellow solid, 21.3 mg (39%). ^1^H-NMR (500 MHz, chloroform-*d*): δ = 11.78 (s, 1H), 8.08–8.05 (m, 2H), 7.47–7.45 (m, 2H), overlapped with CDCl3 signal 7.28–7.25 (m, 2H), 6.92 (d, *J* = 8.7 Hz, 2H), 3.86 (s, 3H), 3.69 (s, 3H), 3.18 (t, *J* = 6.9 Hz, 2H), 2.83 (t, *J* = 6.9 Hz, 2H) ppm. ^13^C{^1^H}-NMR (126 MHz, chloroform-*d*): δ = 173.5, 161.3, 156.3, 155.6, 146.6, 144.1, 135.8, 130.8, 129.9, 129.0, 123.8, 123.2, 114.8, 55.4, 51.6, 29.9, 27.4 ppm. IR (DRIFT): ν_max_ = 1732 (C=O); 1639 (C=O); 1596 (N–H); 1513 (N–O); 1342 (N–O); 1301 (C=N); 1256 (C–O); 1175 (C–O); 1109 (C–O); 1027 (C–O) cm^–1^. HRMS (ESI-Orbitrap) *m*/*z*: [M + H]^+^ calcd for C_21_H_20_N_3_O_6_^+^ 410.1347; found, 410.1340 and [M – H]^−^ calcd for C_21_H_18_N_3_O_6_^–^ 408.1197; found, 408.1190.

#### 5-(4-Nitrophenyl)-6-(*p*-tolyl)pyrazin-2(1*H*)-one (**7c**)

Yellow solid, 22 mg (53%). ^1^H-NMR (500 MHz, DMSO-*d*_6_): δ = 12.48 (s, 1H), 8.14 (s, 1H), 8.10–8.07 (m, 2H), 7.46 (d, *J* = 8.7 Hz, 2H), 7.24 (d, *J* = 8.2 Hz, 2H), 7.19 (d, *J* = 8.0 Hz, 2H), 2.32 (s, 3H) ppm. ^13^C{^1^H} NMR (126 MHz, DMSO-*d*_6_): δ = 156.9, 146.0, 144.8, 139.3, 130.1, 129.6, 129.1, 123.1, 20.9 ppm. IR (DRIFT): ν_max_ = 1645 (C=O); 1591 (N–H); 1509 (N–O); 1342 (N–O); 1259 (N=C) cm^–1^. HRMS (ESI-Orbitrap) *m*/*z*: [M + H]^+^ calcd for C_17_H_14_N_3_O_3_^+^ 308.1030; found, 308.1031 and [M-H]—calcd for C_17_H_12_N_3_O_3_^–^ 306.0873; found, 306.0882.

#### 3-Methyl-5-(4-nitrophenyl)-6-(*p*-tolyl)pyrazin-2(1*H*)-one (**7d**)

Yellow solid, 18 mg (42%). ^1^H-NMR (500 MHz, chloroform-*d*): δ = 11.65 (s, 1H), 8.07 (dt, *J* = 9.3, 2.3 Hz, 2H), 7.47 (dt, *J* = 9.3, 2.3 Hz, 2H), 7.22–7.18 (m, 4H), 2.51 (s, 3H), 2.40 (s, 3H) ppm. ^13^C{^1^H} NMR (126 MHz, chloroform-*d*): δ = 156.7, 155.8, 146.7, 144.1, 140.8, 135.7, 130.1, 130.0, 129.5, 129.1, 129.0, 123.3, 21.4, 20.0 ppm. IR (DRIFT): ν_max_ = 1645 (C=O); 1590 (N–H); 1510 (N–O); 1339 (N–O); 1250 (C=N) cm^–1^. HRMS (ESI-Orbitrap) *m*/*z*: [M + H]^+^ calcd for C_18_H_16_N_3_O_3_^+^ 322.1186; found, 322.1185 and [M-H]—calcd for C_18_H_14_N_3_O_3_^–^ 320.1030; found, 320.1035.

#### 3-Isobutyl-5-(4-nitrophenyl)-6-(*p*-tolyl)pyrazin-2(1*H*)-one (**7e**)

Yellow solid, 19.3 mg (39%). ^1^H-NMR (500 MHz, chloroform-*d*): δ 11.65 (s, 1H), 8.07 (dt, *J* = 9.3, 2.2 Hz, 2H), 7.50–7.47 (m, 2H), 7.24–7.19 (m, 4H), 2.73 (d, *J* = 7.0 Hz, 2H), 2.41 (s, 3H), 2.31–2.22 (m, 1H), 1.01 (d, *J* = 6.6 Hz, 6H) ppm. ^13^C{^1^H} NMR (126 MHz, chloroform-*d*): δ = 158.3, 156.8, 146.8, 144.3, 140.9, 135.7, 130.2, 130.1, 129.5, 129.3, 129.2, 123.4, 41.8, 26.9, 22.9, 21.6 ppm. IR (DRIFT): ν_max_ = 1644 (C=O); 1592 (N–H); 1513 (N–O); 1340 (N–O); 1255 (C=N); cm^–1^. HRMS (ESI-Orbitrap) *m*/*z*: [M + H]^+^ calcd for C_21_H_22_N_3_O_3_^+^ 364.1656; found, 364.1653 and [M – H]^−^ calcd for C_21_H_20_N_3_O_3_^–^ 362.1499; found, 362.1508.

#### 3-Benzyl-5-(4-nitrophenyl)-6-(*p*-tolyl)pyrazin-2(1*H*)-one (**7f**)

Yellow solid, 23 mg (43%). ^1^H-NMR (500 MHz, chloroform-*d*): δ 12.26 (s, 1H), 8.09–8.06 (m, 2H), 7.48–7.45 (m, 2H), 7.36–7.34 (m, 2H), overlapped with CDCl_3_ signal 7.27–7.19 (m, 5H), 4.13 (s, 2H), 2.43 (s, 3H) ppm. ^13^C{^1^H} NMR (126 MHz, chloroform-*d*): δ = 156.54, 156.46, 146.7, 144.0, 140.9, 137.2, 136.4, 130.0, 129.9, 129.7, 129.5, 129.3, 128.9, 128.3, 126.6, 123.3, 21.5. IR (DRIFT): ν_max_ = 1645 (C=O); 1591 (N–H); 1512 (N–O); 1339 (N–O); 1258 (C=N) cm^–1^. HRMS (ESI-Orbitrap) *m*/*z*: [M + H]^+^ calcd for C_24_H_20_N_3_O_3_^+^ 398.1499; found, 398.1497 and [M – H]^−^ calcd for C_24_H_18_N_3_O_3_^–^ 396.1343; found, 396.1348.

#### 3-(4-Hydroxybenzyl)-5-(4-nitrophenyl)-6-(*p*-tolyl)pyrazin-2(1*H*)-one (**7g**)

Yellow solid, 17.5 mg (31%). ^1^H-NMR (500 MHz, DMSO-*d*_6_): δ = 12.47 (s, 1H), 9.20 (s, 1H), 8.07 (d, *J* = 8.9 Hz, 2H), 7.42 (d, *J* = 8.6 Hz, 2H), 7.22–7.15 (m, 6H), 6.70–6.67 (m, 2H), 3.96 (s, 2H), 2.32 (s, 3H) ppm. ^13^C{^1^H} NMR (126 MHz, DMSO-*d*_6_): δ = 155.8, 155.4, 145.8, 144.8, 139.3, 130.03, 129.98, 129.6, 129.2, 127.7, 123.0, 115.1, 37.6, 20.9 ppm. IR (DRIFT): ν_max_ = 3439 (O–H); 1643 (C=O); 1591 (N–H); 1512 (N–O); 1339 (N–O); 1314 (C=N) cm^–1^. HRMS (ESI-Orbitrap) *m*/*z*: [M + H]^+^ calcd for C_24_H_20_N_3_O_4_^+^ 414.1448; found, 414.1440 and [M – H]^−^ calcd for C_24_H_18_N_3_O_4_^–^ 412.1292; found, 412.1300.

#### 3-[2-(Methylthio)ethyl]-5-(4-nitrophenyl)-6-(*p*-tolyl)pyrazin-2(1*H*)-one (**7h**)

Yellow solid, 13.8 mg (27%). ^1^H-NMR (500 MHz, DMSO-*d*_6_): δ = 12.48 (s, 1H), 8.07 (d, *J* = 8.9 Hz, 2H), 7.44 (d, *J* = 8.9 Hz, 2H), 7.23–7.19 (m, 4H), 3.05 (t, *J* = 7.3 Hz, 2H), 2.90 (t, *J* = 7.3 Hz, 2H), 2.33 (s, 3H), 2.11 (s, 3H) ppm. ^13^C{^1^H} NMR (126 MHz, DMSO-*d*_6_): δ = 155.6, 145.8, 144.8, 139.4, 130.0, 129.6, 129.2, 123.0, 32.0, 30.0, 20.9, 14.7 ppm. IR (DRIFT): ν_max_ = 1640 (C=O); 1590 (N–H); 1511 (N–O); 1340 (N–O); 1252 (C=N) cm^–1^. HRMS (ESI-Orbitrap) *m*/*z*: [M + H]^+^ calcd for C_20_H_20_N_3_O_3_S^+^ 382.1220; found, 382.1223 and [M – H]^−^ calcd for C_20_H_18_N_3_O_3_S^–^ 380.1063; found, 380.1072.

#### 3-[6-(4-Nitrophenyl)-3-oxo-5-(*p*-tolyl)-3,4-dihydropyrazin-2-yl]propanamide (**7i**)

Yellow solid, 13.2 mg (26%). ^1^H-NMR (500 MHz, DMSO-*d*_6_): δ = 12.45 (s, 1H), 8.07–8.04 (m, 2H), 7.46–7.43 (m, 2H), 7.37 (s, 1H), 7.24–7.18 (m, 4H), 6.77 (s, 1H), 2.97 (t, *J* = 7.3 Hz, 2H), 2.55 (t, *J* = 7.3 Hz, 2H), 2.33 (s, 3H) ppm. ^13^C{^1^H} NMR (126 MHz, DMSO-*d*_6_): δ = 173.6, 155.4, 145.7, 144.8, 139.4, 130.0, 129.6, 129.3, 122.9, 30.9, 27.5, 20.9 ppm. IR (DRIFT): ν_max_ = 3396 (N–H); 3178 (N–H); 1658 (C=O); 1644 (C=O); 1591 (N–H); 1510 (N–O); 1342 (N–O); 1291 (C=N) cm^–1^. HRMS (ESI-Orbitrap) *m*/*z*: [M + H]^+^ calcd for C_20_H_19_N_4_O_4_^+^ 379.1401; found, 379.1396 and [M – H]^−^ calcd for C_20_H_17_N_4_O_4_^–^ 377.1244; found, 377.1252.

#### 1-{3-[6-(4-Nitrophenyl)-3-oxo-5-(*p*-tolyl)-3,4-dihydropyrazin-2-yl]propyl}guanidine (**7j**)

Yellow solid, 18.1 mg (33%). ^1^H-NMR (500 MHz, DMSO-D6): δ = 9.31 (s, 1H), 8.00–7.97 (m, 2H), 7.65 (s, 3H), 7.42–7.39 (m, 2H), 7.12 (dd, *J* = 15.6, 8.2 Hz, 4H), 3.17–3.13 (m, 2H), 2.72 (t, *J* = 7.4 Hz, 2H), 2.28 (s, 3H), 1.91–1.86 (m, 2H), 1.78 (s, 1H) ppm. ^13^C{^1^H} NMR (126 MHz, DMSO-*d*_6_): δ = 157.3, 146.6, 145.0, 137.9, 129.5, 129.4, 129.0, 128.8, 122.9, 40.5, 29.5, 26.00, 22.1 ppm. Ammonium acetate buffer residue. IR (DRIFT): ν_max_ = 2995 (N–H); 2881 (N–H); 1658 (C=N); 1634 (C=O); 1594 (N–H); 1514 (N–O); 1338 (N–O); 1310 (C=N) cm^–1^. HRMS (ESI-Orbitrap) *m*/*z*: [M + H]^+^ calcd for C_21_H_23_N_6_O_3_^+^ 407.1826; found, 407.1825 and [M – H]^−^ calcd for C_21_H_21_N_6_O_3_^–^ 405.1670; found, 405.1676.

#### 6-(4-Amino-3,5-dichlorophenyl)-3-methyl-5-(4-nitrophenyl)pyrazin-2(1*H*)-one (**7k**)

Yellow solid, 16.7 mg (32%). ^1^H-NMR (500 MHz, chloroform-*d*): δ = 12.52 (s, 1H), 8.16–8.14 (m, 2H), 7.55 (dt, *J* = 9.2, 2.2 Hz, 2H), 7.21 (s, 2H), 4.78 (s, 2H), 2.55 (s, 3H) ppm. ^13^C{^1^H} NMR (126 MHz, chloroform-*d*): δ = 157.1, 155.8, 150.2, 146.9, 143.8, 141.9, 133.7, 130.1, 128.9, 123.6, 121.1, 119.6, 19.9 ppm. IR (DRIFT): ν_max_ = 3480 (N–H); 3378 (N–H); 1644 (C=O); 1592 (N–H); 1514 (N–O); 1342 (N–O); 1247 (C=N); 882 (C–Cl); 865 (C–Cl) cm^–1^. HRMS (ESI-Orbitrap) *m*/*z*: [M + H]^+^ calcd for C_17_H_13_Cl_2_N_4_O_3_^+^ 391.0359; found, 391.0353 and [M – H]^−^ calcd for C_17_H_11_Cl_2_N_4_O_3_^–^ 389.0203; found, 389.0211.

#### 3-[(1*H*-Indol-3-yl)methyl]-6-(4-methoxyphenyl)-5-(4-nitrophenyl)pyrazin-2(1*H*)-one (**7l**)

Yellow solid, 11.8 mg (19%). ^1^H-NMR (500 MHz, DMSO-*d*_6_): δ = 12.46 (s, 1H), 10.87 (s, 1H), 8.07 (d, *J* = 8.9 Hz, 2H), 7.71 (d, *J* = 7.9 Hz, 1H), 7.42 (d, *J* = 8.4 Hz, 2H), 7.34 (d, *J* = 8.0 Hz, 1H), 7.28–7.18 (m, 3H), 7.08–7.04 (m, 1H), 7.00–6.97 (m, 1H), 6.94–6.91 (m, 2H), 4.18 (s, 2H), 3.76 (s, 3H) ppm. ^13^C{^1^H} NMR (126 MHz, DMSO-*d*_6_): δ = 160.1, 155.4, 145.7, 145.0, 136.1, 131.2, 129.9, 127.3, 123.7, 123.0, 120.8, 118.9, 118.3, 114.1, 111.3, 110.1, 55.2, 28.5 ppm. IR (DRIFT): ν_max_ = 3401 (N–H); 1638 (C=O); 1595 (N–H); 1509 (N–O); 1343 (N–O); 1297 (C=N); 1179 (C–O); 1024 (C–O) cm^–1^. HRMS (ESI-Orbitrap) *m*/*z*: [M + H]^+^ calcd for C_26_H_21_N_4_O_4_^+^ 453.1557; found, 453.1556 and [M – H]^−^ calcd for C_26_H_19_N_4_O_4_^–^ 451.1401; found, 451.1409.

## Data Availability

The data underlying this study are available in the published article and its Supporting Information.

## Supporting Information Available

^1^H and ^13^C NMR spectra of the synthetized compounds (PDF)
