# Supplementary material for: Synthesis of Polysubstituted Pyridines and Pyrazines via Truce–Smiles Rearrangement of Amino Acid-Based 4-Nitrobenzenesulfonamides
Source: J Org Chem. 2023 Feb 16;88(5):3228–37. doi: 10.1021/acs.joc.2c03025 (PMC10017029; doi:10.1021/acs.joc.2c03025)
Supplement: Supplementary file 1 — jo2c03025_si_001.pdf [file jo2c03025_si_001.pdf]

Supporting Information

Synthesis of Polysubstituted Pyridines and Pyrazines *via* Truce-Smiles Rearrangement of Amino Acid-based 4-nitrobenzenesulfonamides

Michaela Tkadlecová,[#] Barbora Lemrová[#] and Miroslav Soural[*]

Department of Organic Chemistry, Faculty of Science, Palacký University, 771 46 Olomouc, Czech Republic

*Corresponding author. E-mail: miroslav.soural@upol.cz

[#] Equal contribution

Table of Contents:

NMR spectra of final compounds……………………………………………………………………………….S2-S26

HRMS spectra of final compounds……………………………………………………………………………...S27-S51

HPLC-UV spectra of crude final compounds………………………………………………………………….S52-S60

NMR spectra of final compounds:

6-carbamoyl-2-(4-nitrophenyl)-3-(*p*-tolyl)isonicotinic acid (6a)

^{1}H NMR (500Mhz; DMSO-d_6) spectrum of **6a**

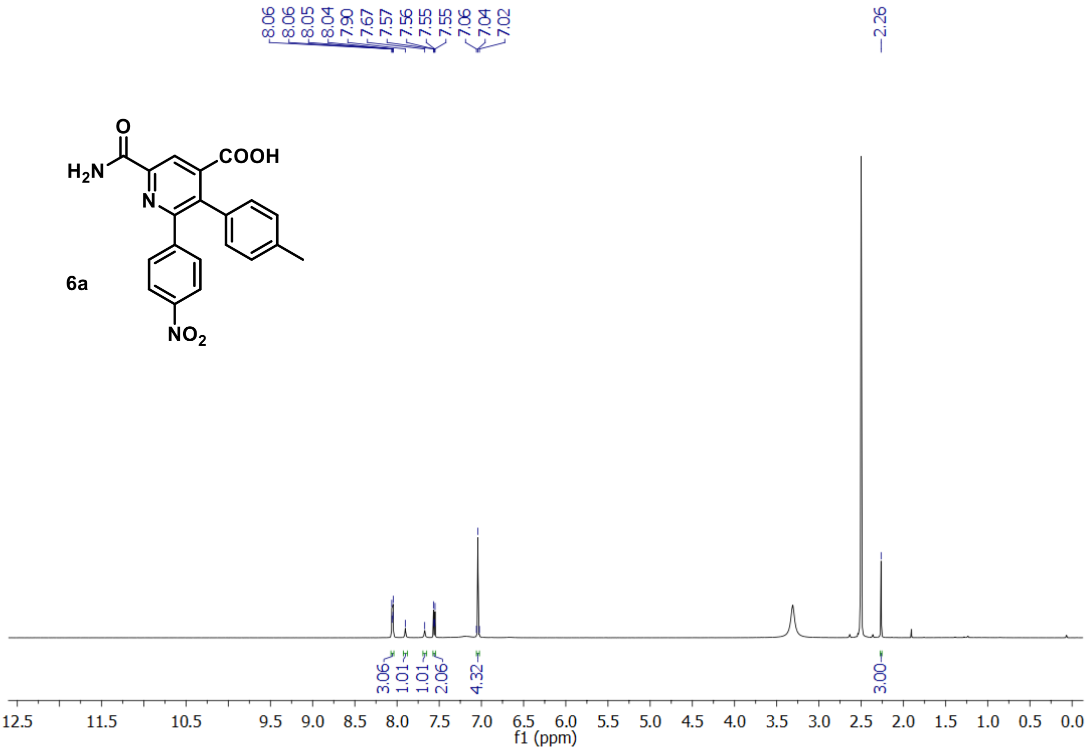

^{13}C{^{1}H} NMR (126 MHz; DMSO-d_6) spectrum of **6a**

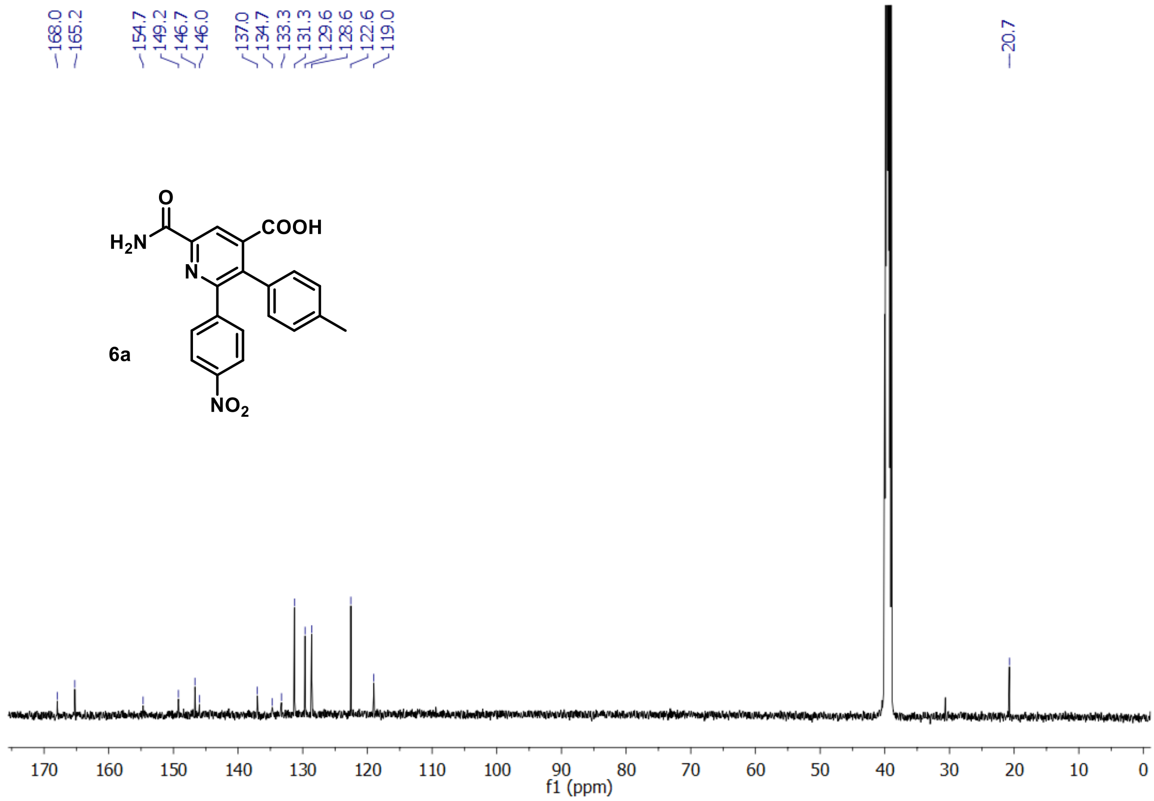

6-carbamoyl-3-(4-methoxyphenyl)-2-(4-nitrophenyl)isonicotinic acid (6b)

^{1}H NMR (500Mhz; DMSO-d_6) spectrum of **6b**

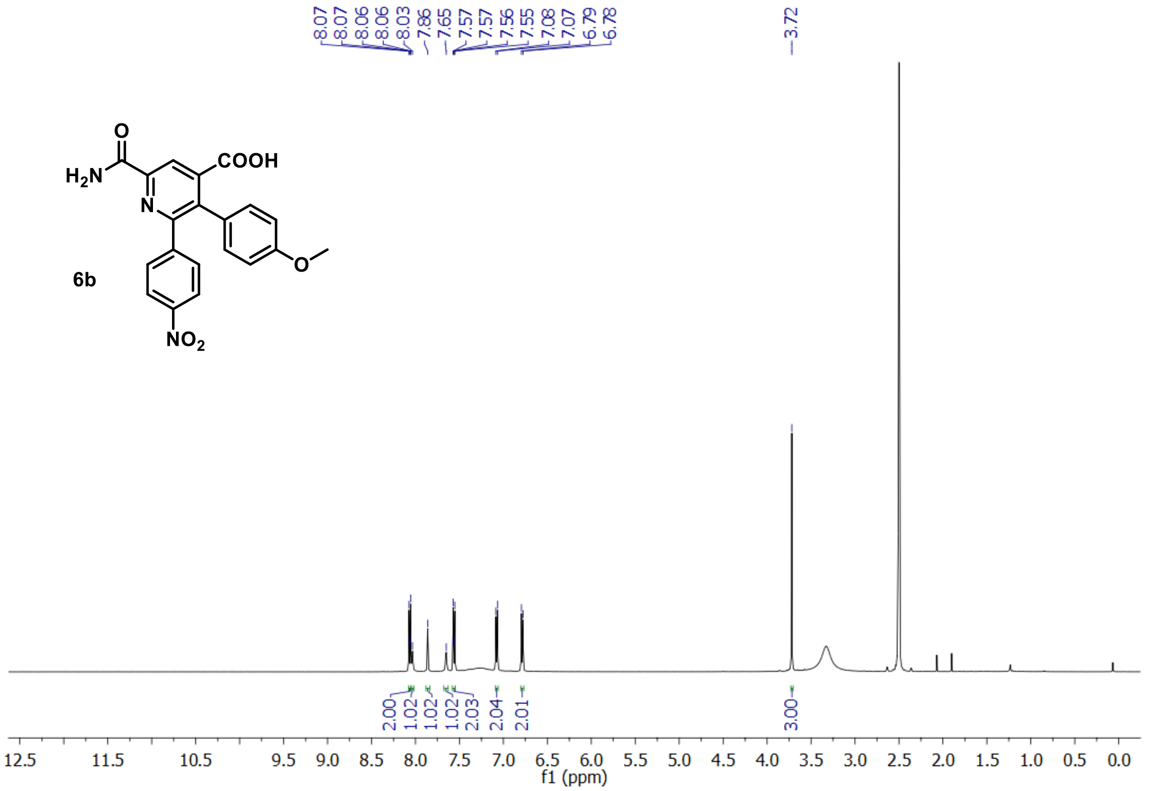

^{13}C{^{1}H} NMR (126 MHz; DMSO-d_6) spectrum of **6b**

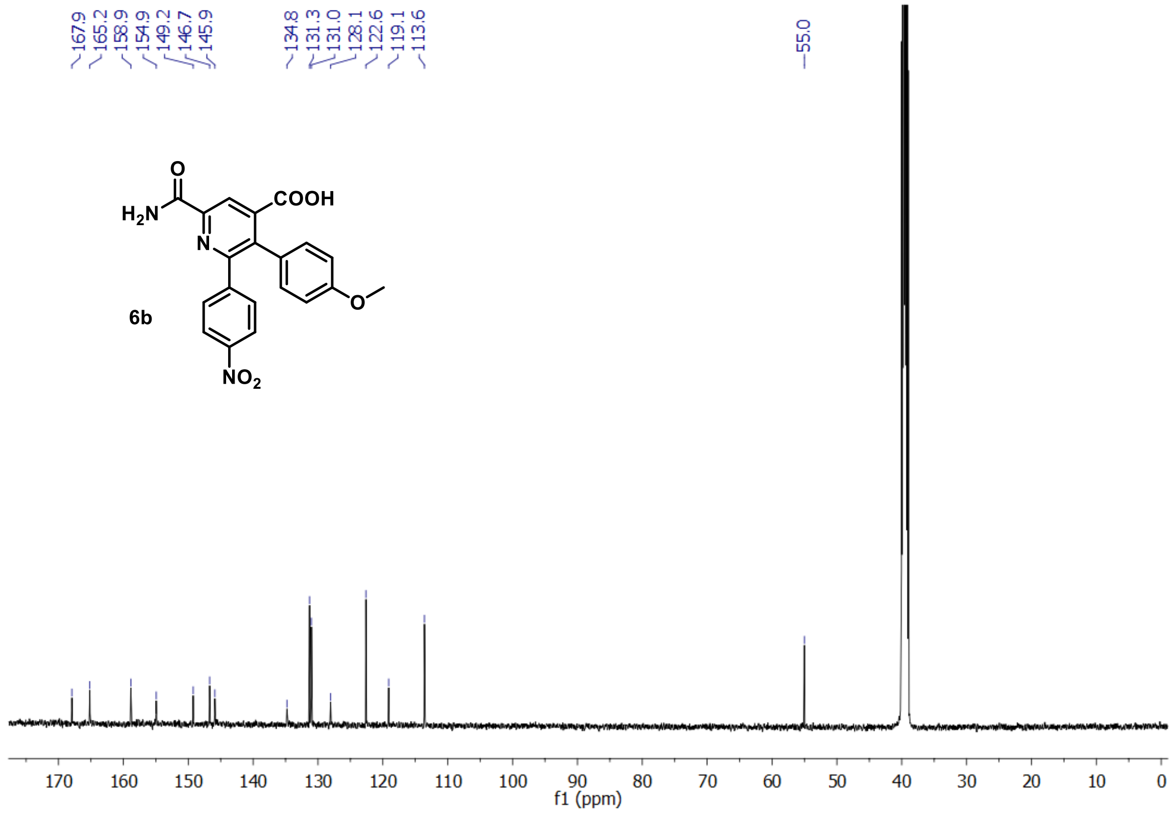

6-(3-carbamoylpyrrolidine-1-carbonyl)-2-(4-nitrophenyl)-3-(p-tolyl)isonicotinic acid (6c)

^{1}H NMR (400Mhz; DMSO-d_6 at 25°C) spectrum of **6c**

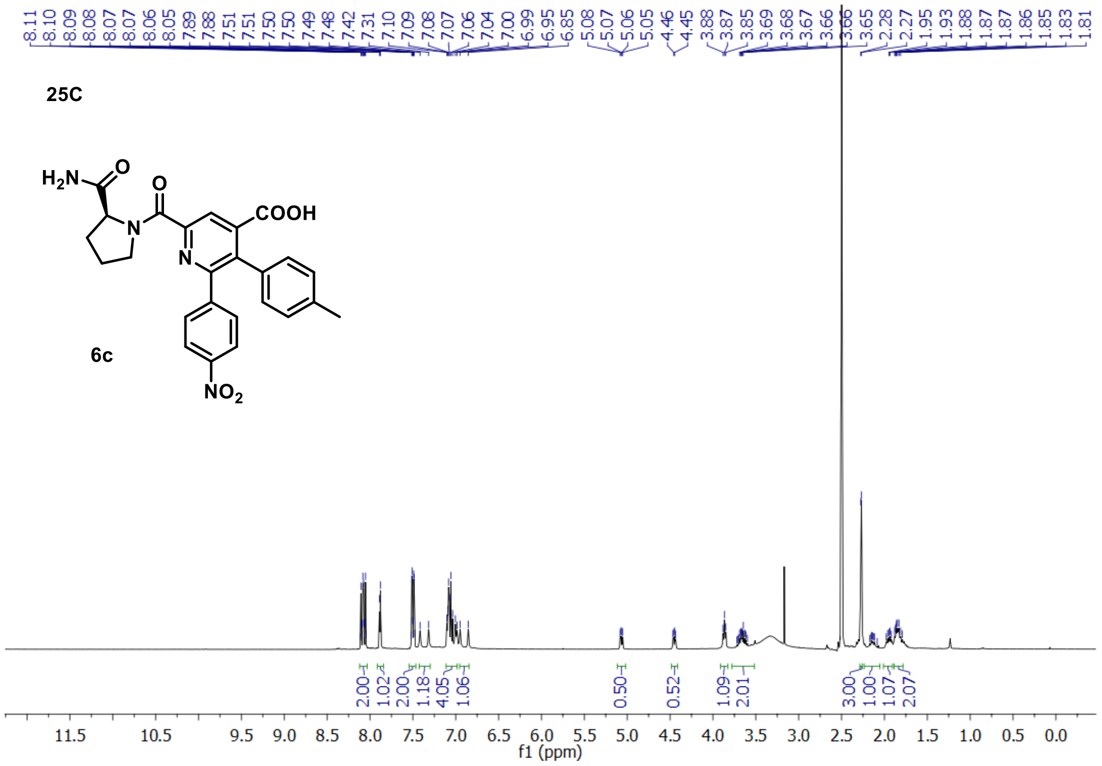

^{1}H NMR (500Mhz; DMSO-d_6 at 120°C) spectrum of **6c**

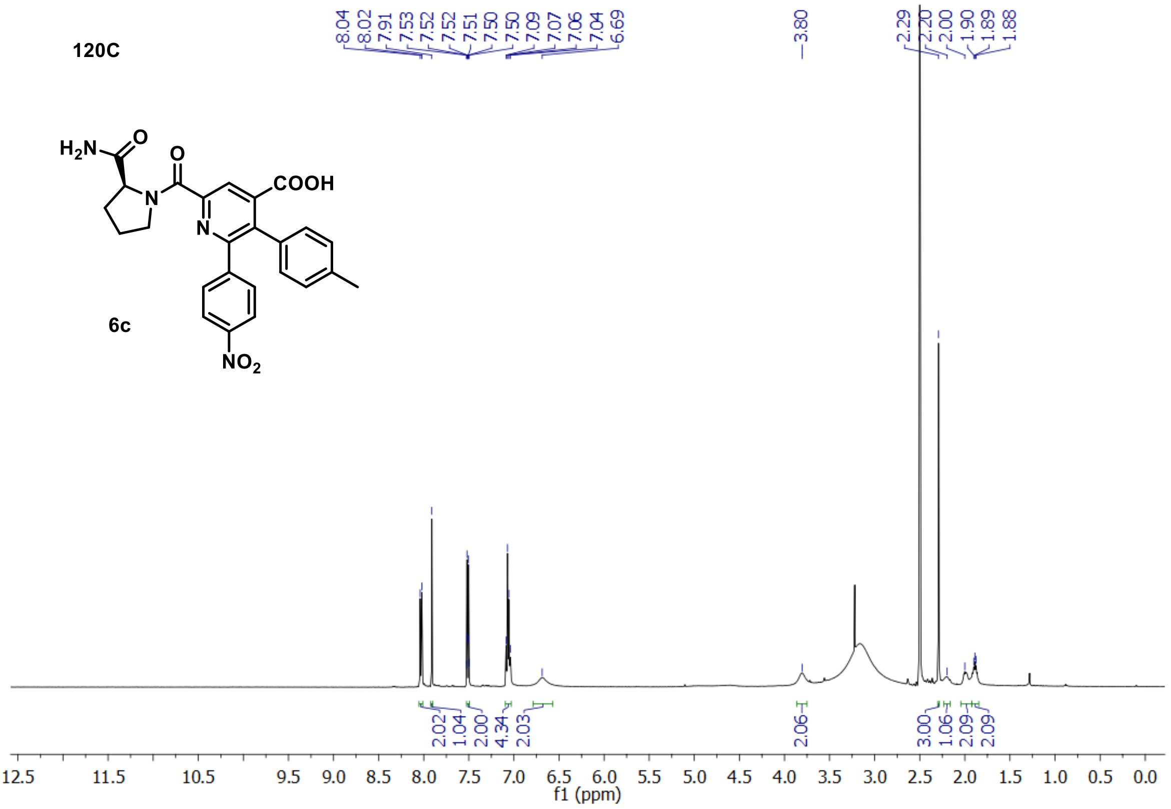

$^{13}C\{^1H\}$ NMR (101 MHz; DMSO-d_6 at 25°C) spectrum of **6c**

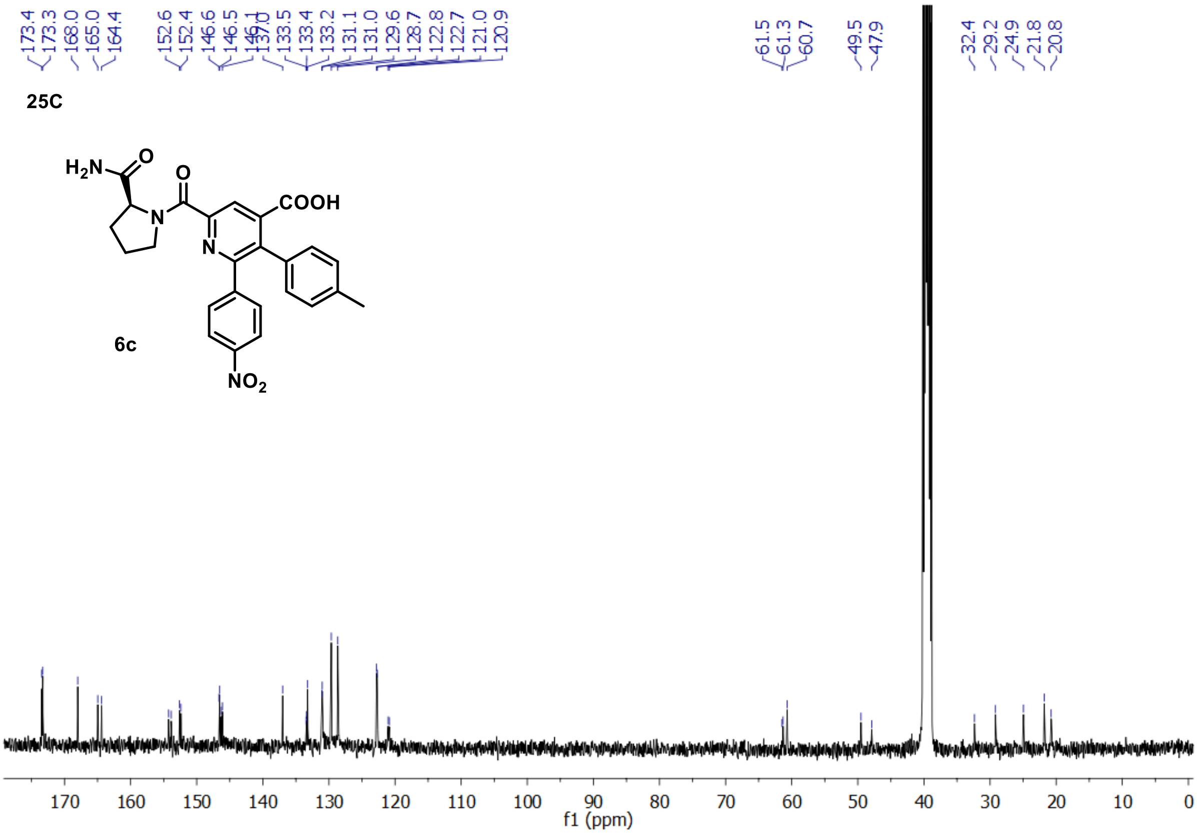

3-(4-fluorophenyl)-2-(4-nitrophenyl)-6-(piperazine-1-carbonyl)isonicotinic acid (6d)

^{1}H NMR (500Mhz; DMSO-d_6) spectrum of **6d**

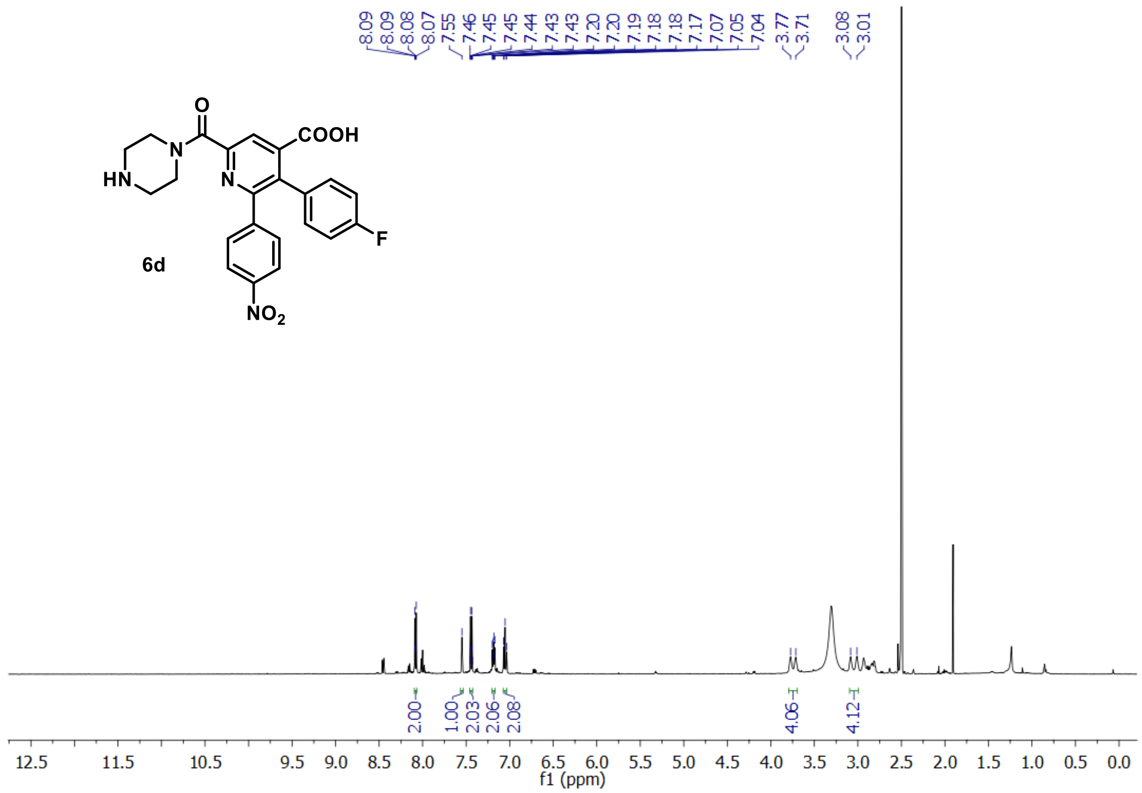

^{13}C{^{1}H} NMR (126 MHz; TRIFLUOROACETIC ACID-D) spectrum of **6d**

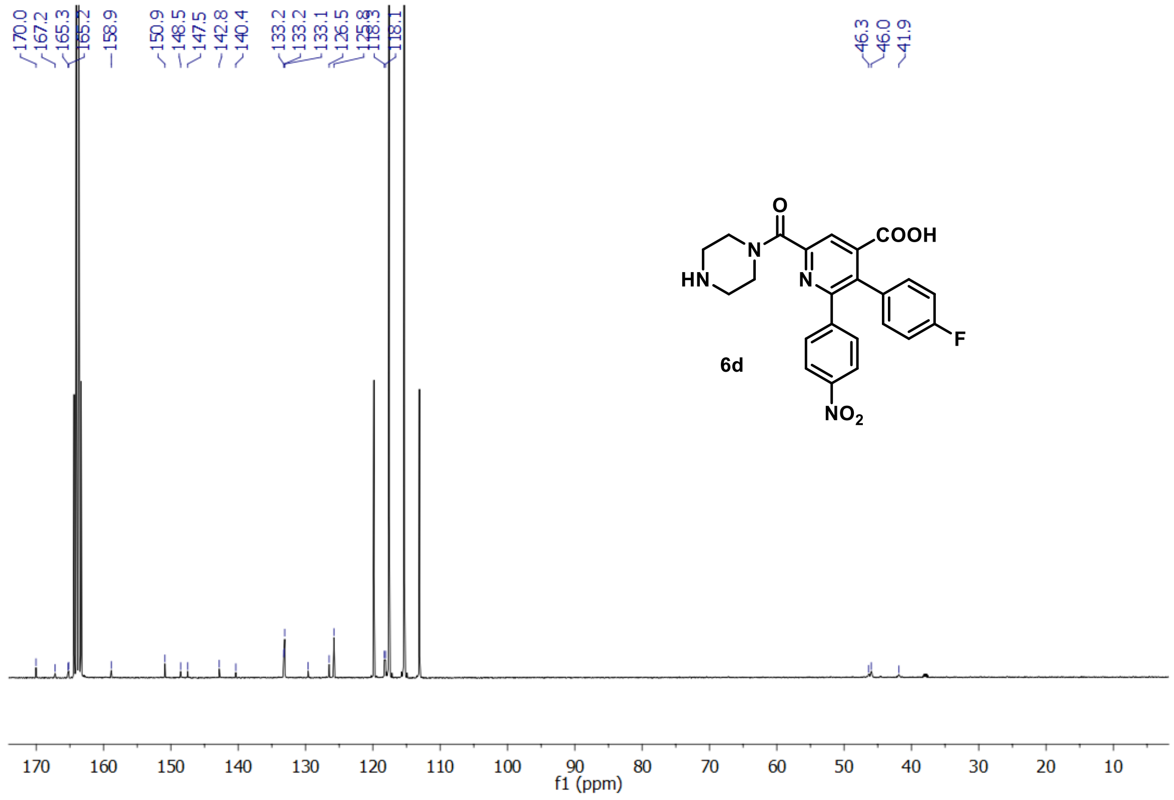

2-(4-nitrophenyl)-6-(piperazine-1-carbonyl)-3-(*p*-tolyl)isonicotinic acid (6e)

^{1}H NMR (500Mhz; DMSO-*d*$_6$) spectrum of **6e**

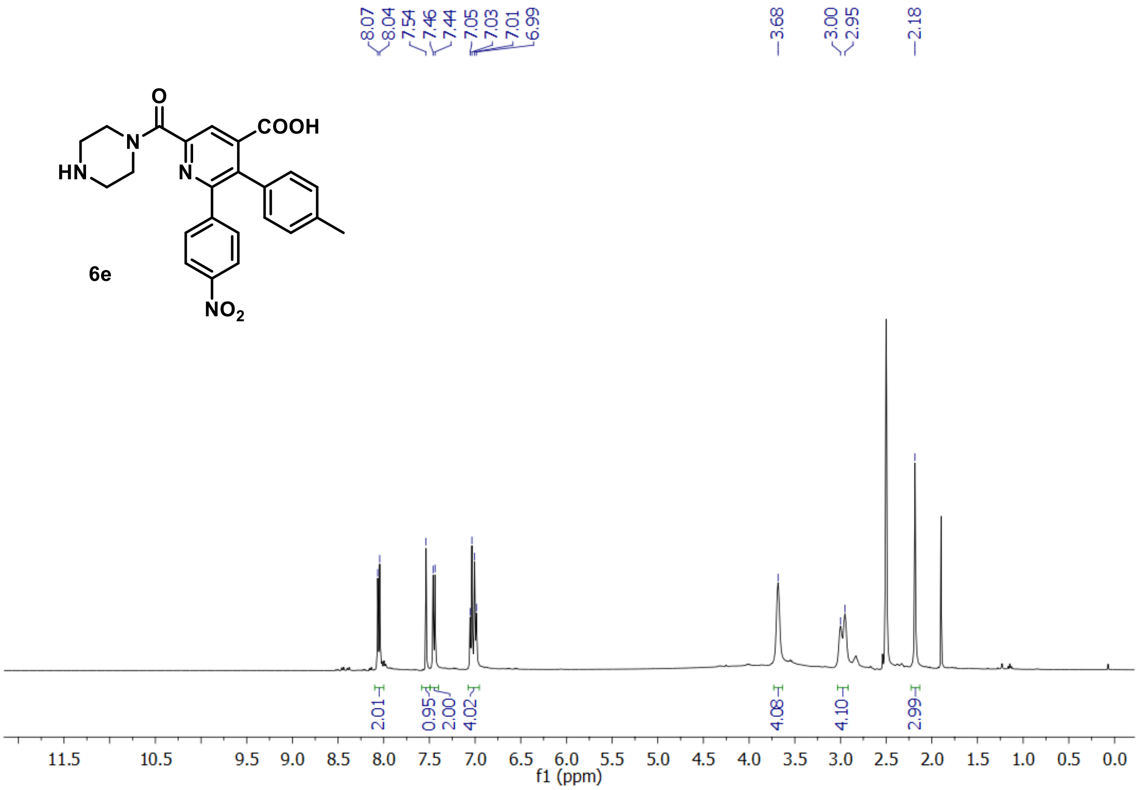

^{13}C{^{1}H} NMR (126 MHz; DMSO-*d*$_6$) spectrum of **6e**

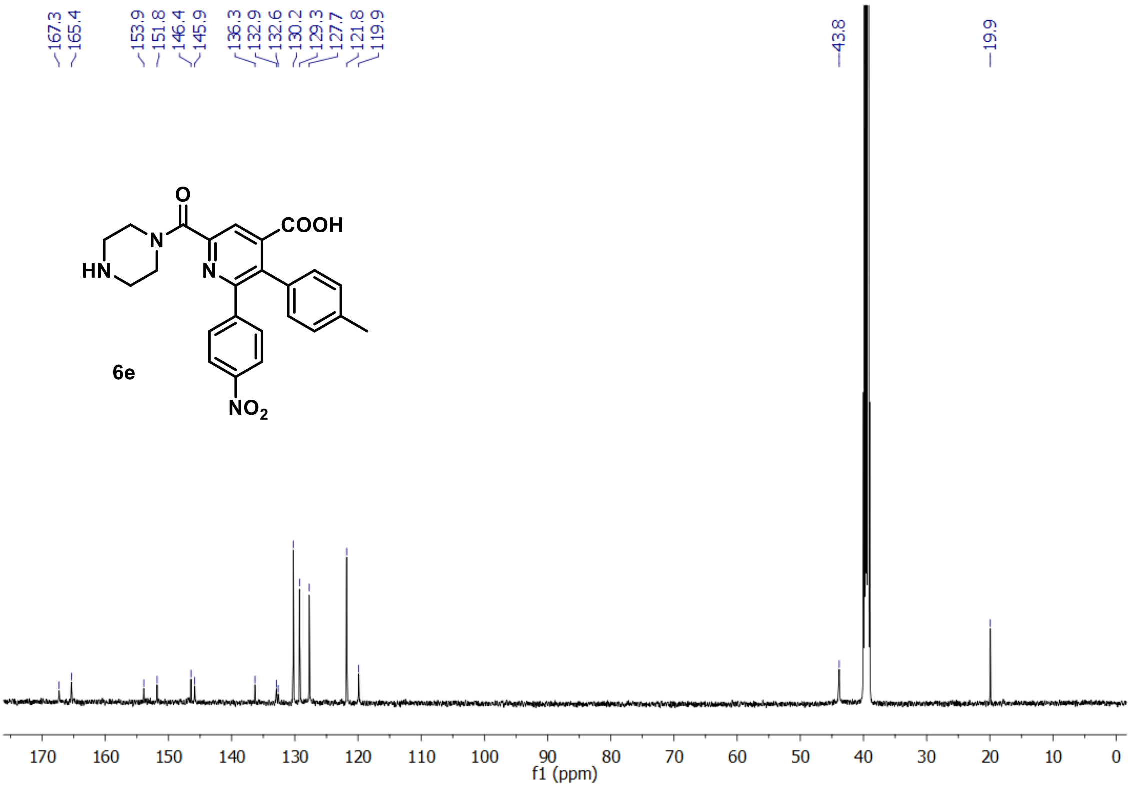

3-(3-methoxyphenyl)-2-(4-nitrophenyl)-6-(piperazine-1-carbonyl)isonicotinic acid (6f)

^{1}H NMR (500Mhz; DMSO-d_6) spectrum of **6f**

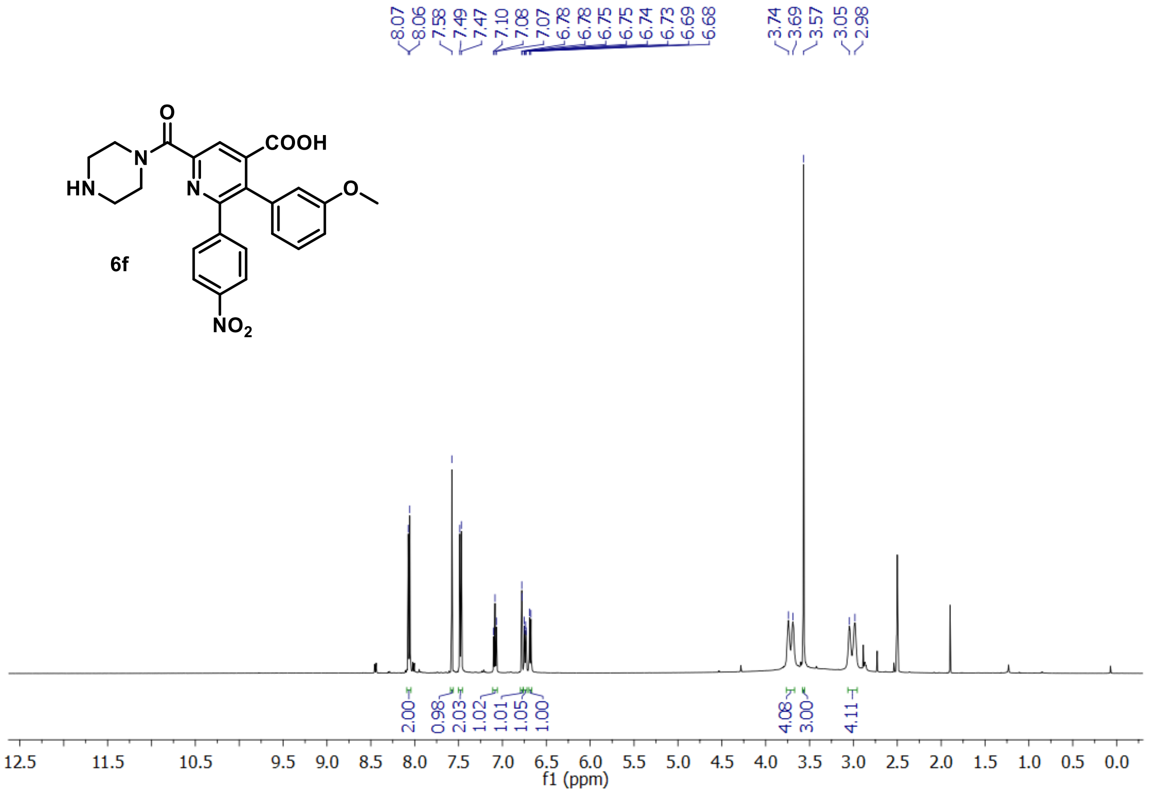

$^{13}C\{^{1}H\}$ NMR (126 MHz; DMSO-d_6) spectrum of **6f**

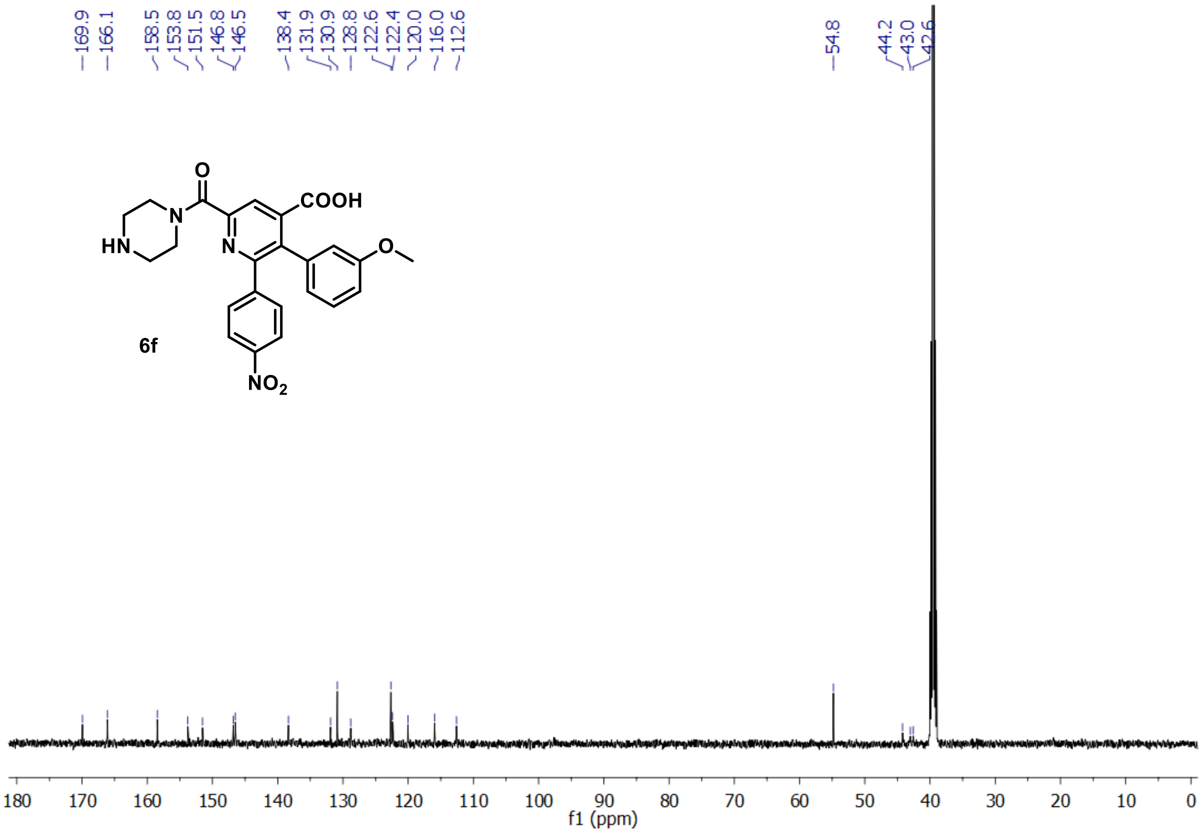

3-(4-amino-3,5-dichlorophenyl)-2-(4-nitrophenyl)-6-(piperazine-1-carbonyl)isonicotinic acid (6g)

^{1}H NMR (500Mhz; DMSO-d_6) spectrum of **6g**

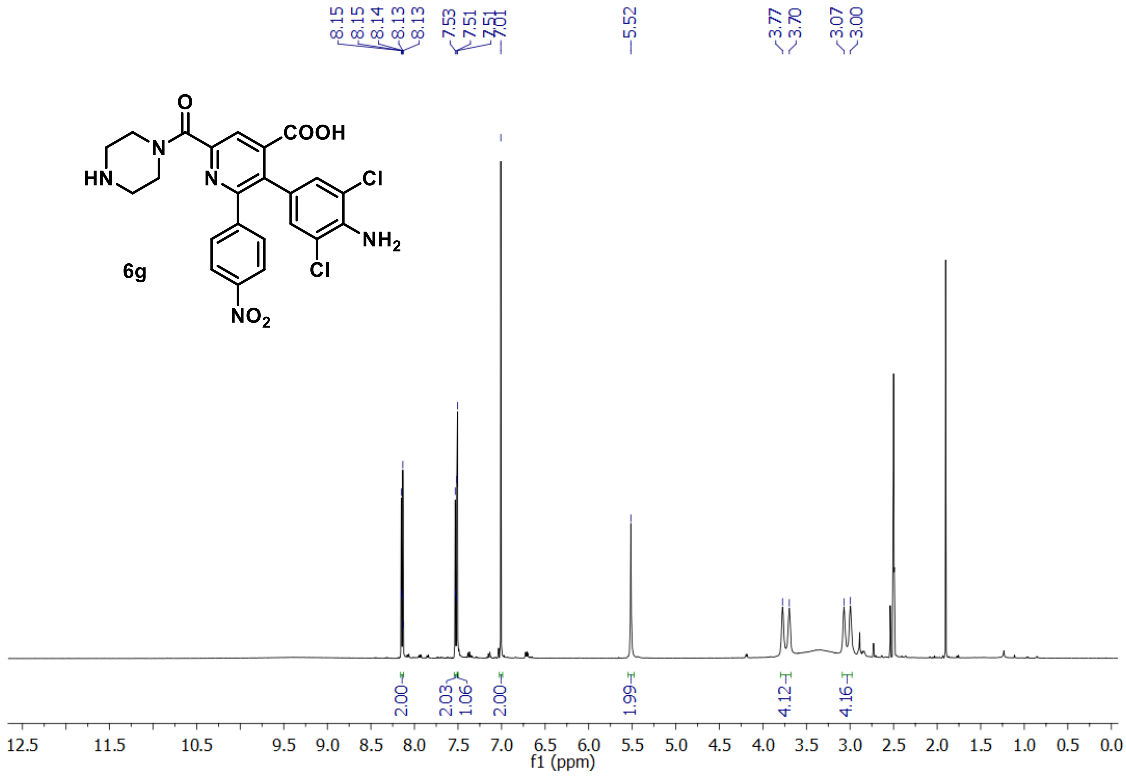

$^{13}C\{^{1}H\}$ NMR (126 MHz; DMSO-d_6) spectrum of **6g**

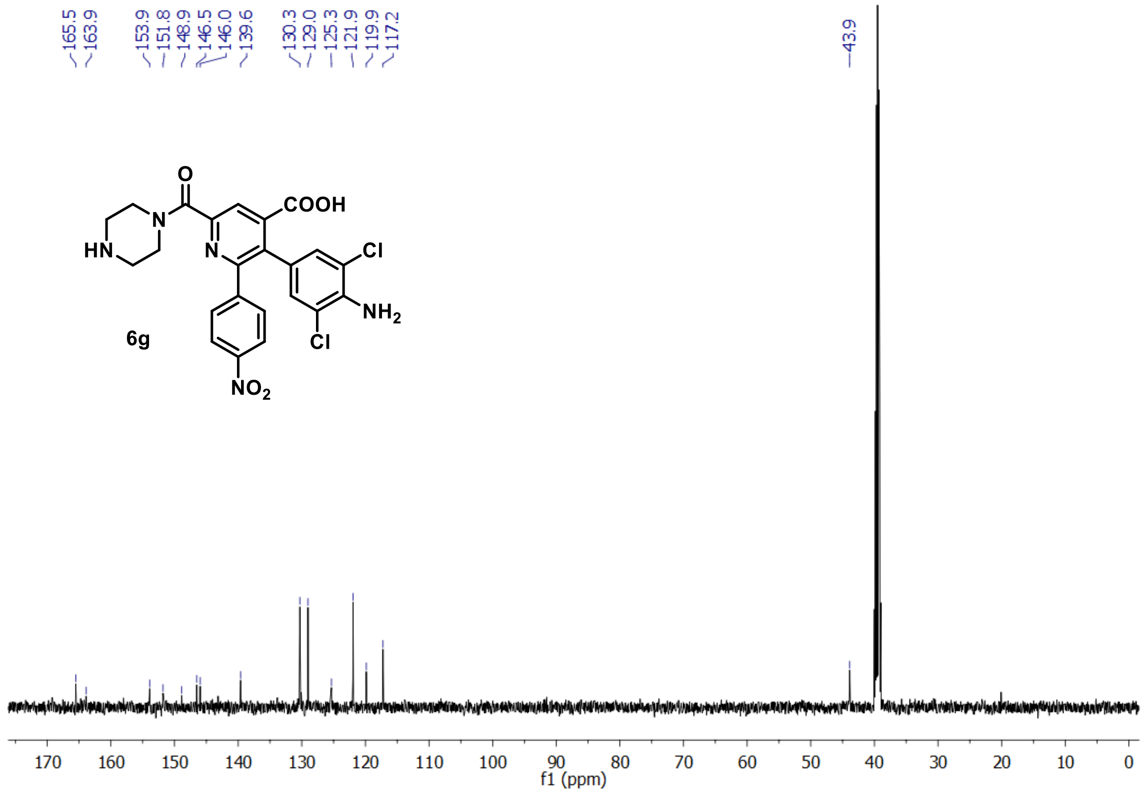

ethyl 3-(3-methoxyphenyl)-2-(4-nitrophenyl)-6-(piperazine-1-carbonyl)isonicotinate (6h)

^{1}H NMR (500Mhz; DMSO-d_6) spectrum of **6h**

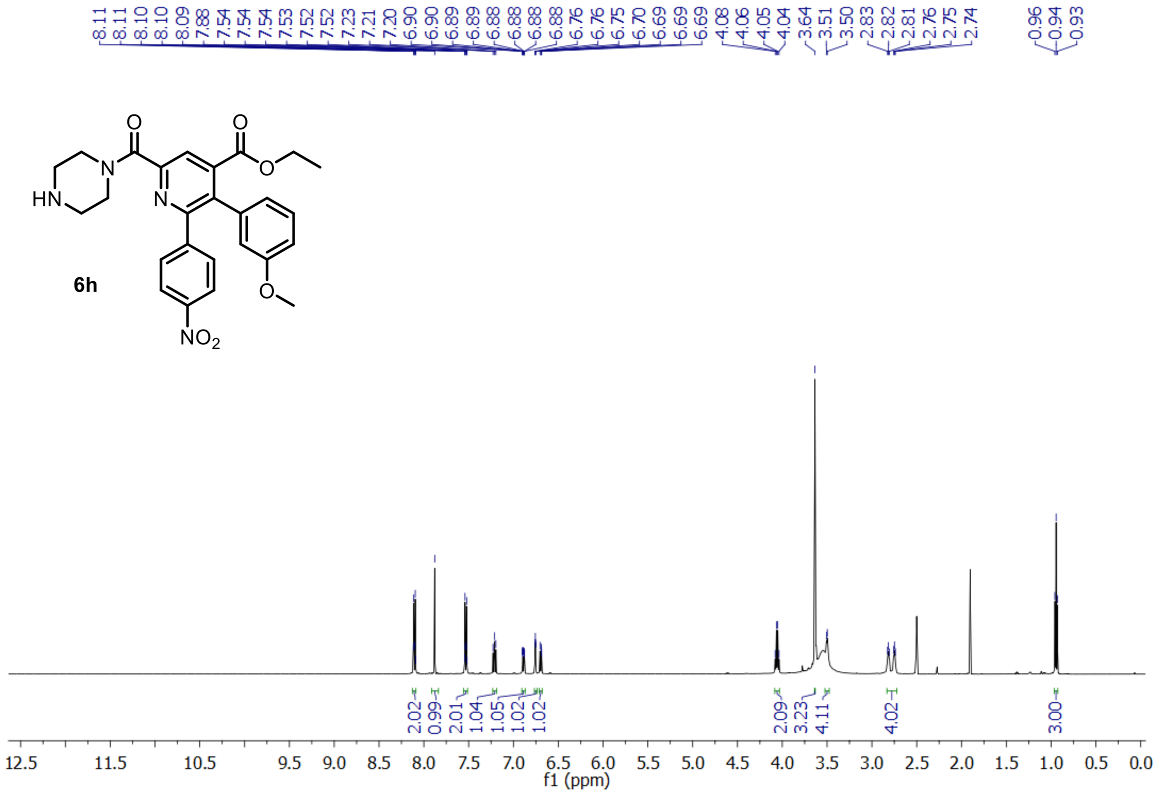

^{13}C{^{1}H} NMR (126 MHz; DMSO-d_6) spectrum of **6h**

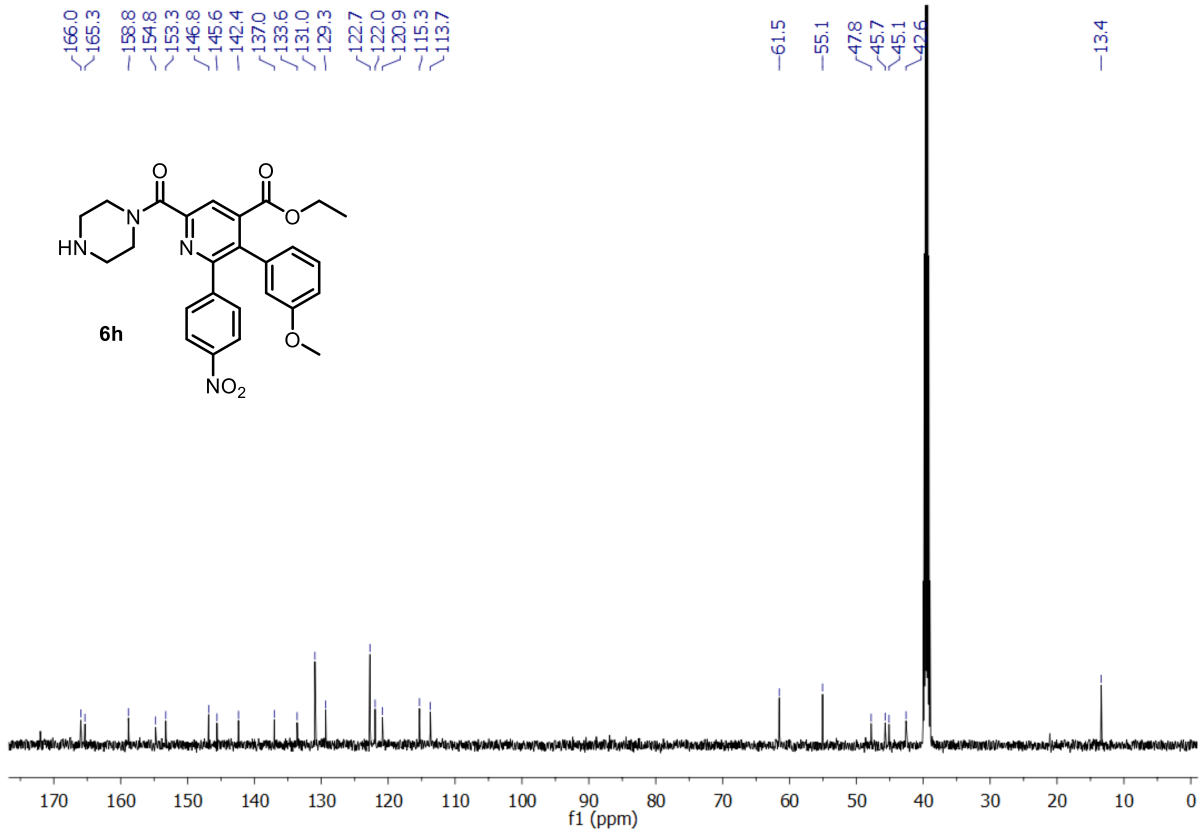

(3-(3-methoxyphenyl)-2-(4-nitrophenyl)-6-(piperazine-1-carbonyl)pyridin-4-yl)(piperidin-1-yl)methanone (6i)

^{1}H NMR (500Mhz; DMSO-d_6) spectrum of **6i**

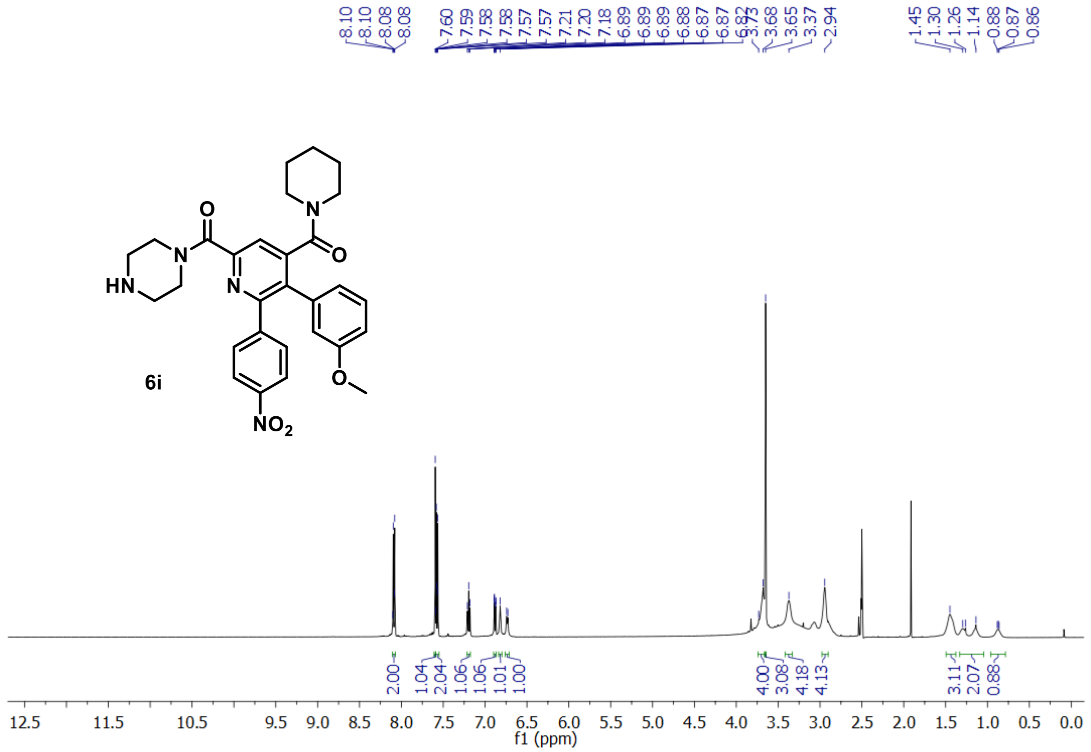

$^{13}C\{^{1}H\}$ NMR (126 MHz; DMSO-d_6) spectrum of **6i**

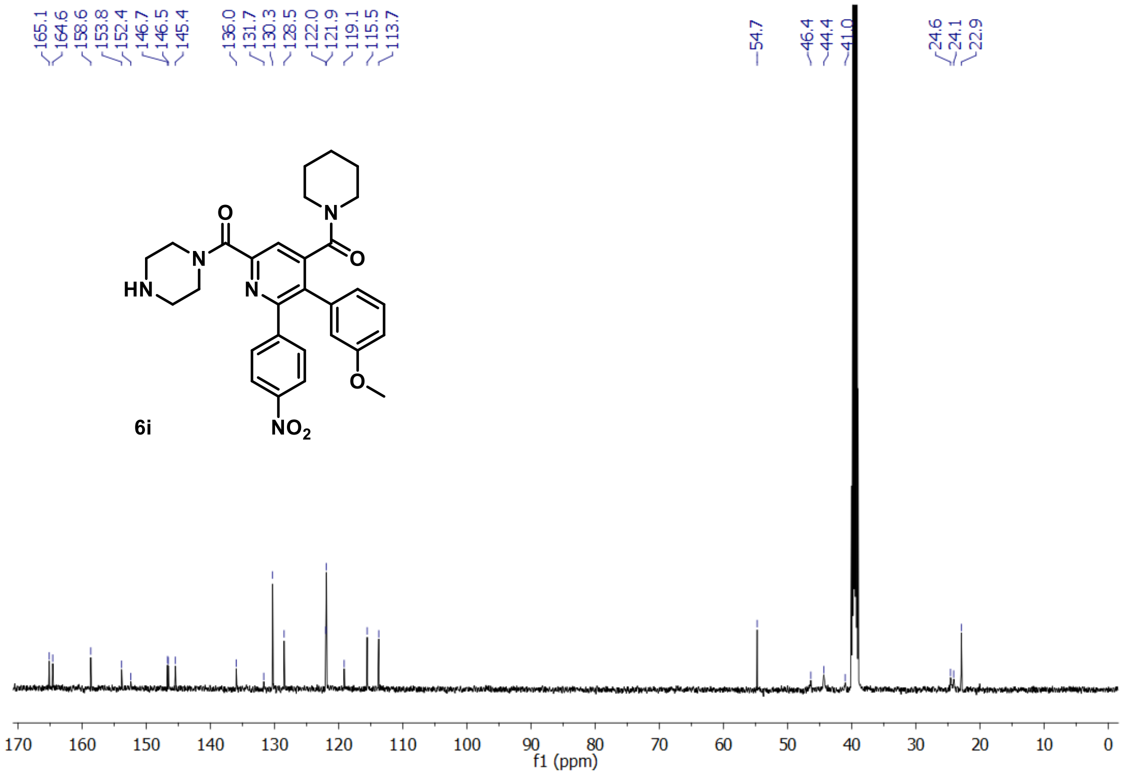

2-(4-nitrophenyl)-6-(piperazine-1-carbonyl)-3-(thiophen-3-yl)isonicotinic acid (6j)

^{1}H NMR (500Mhz; DMSO-d_6) spectrum of **6j**

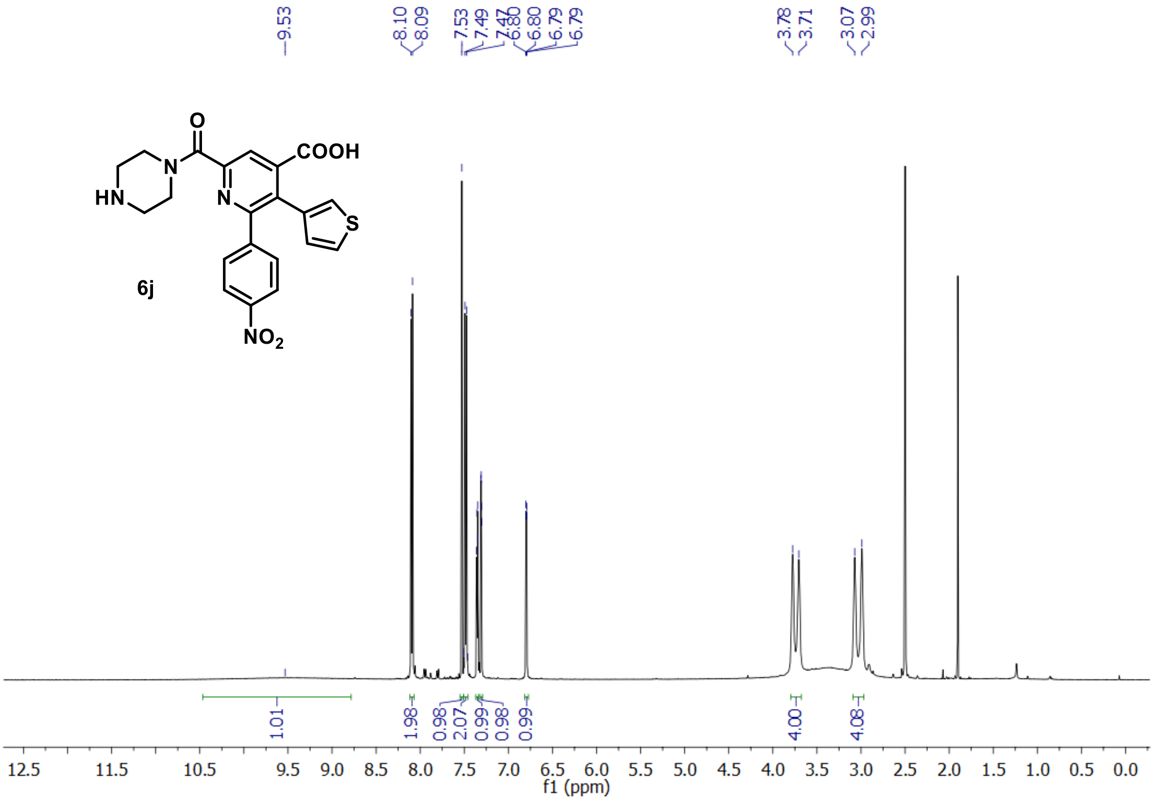

$^{13}C\{^{1}H\}$ NMR (126 MHz; DMSO-d_6) spectrum of **6j**

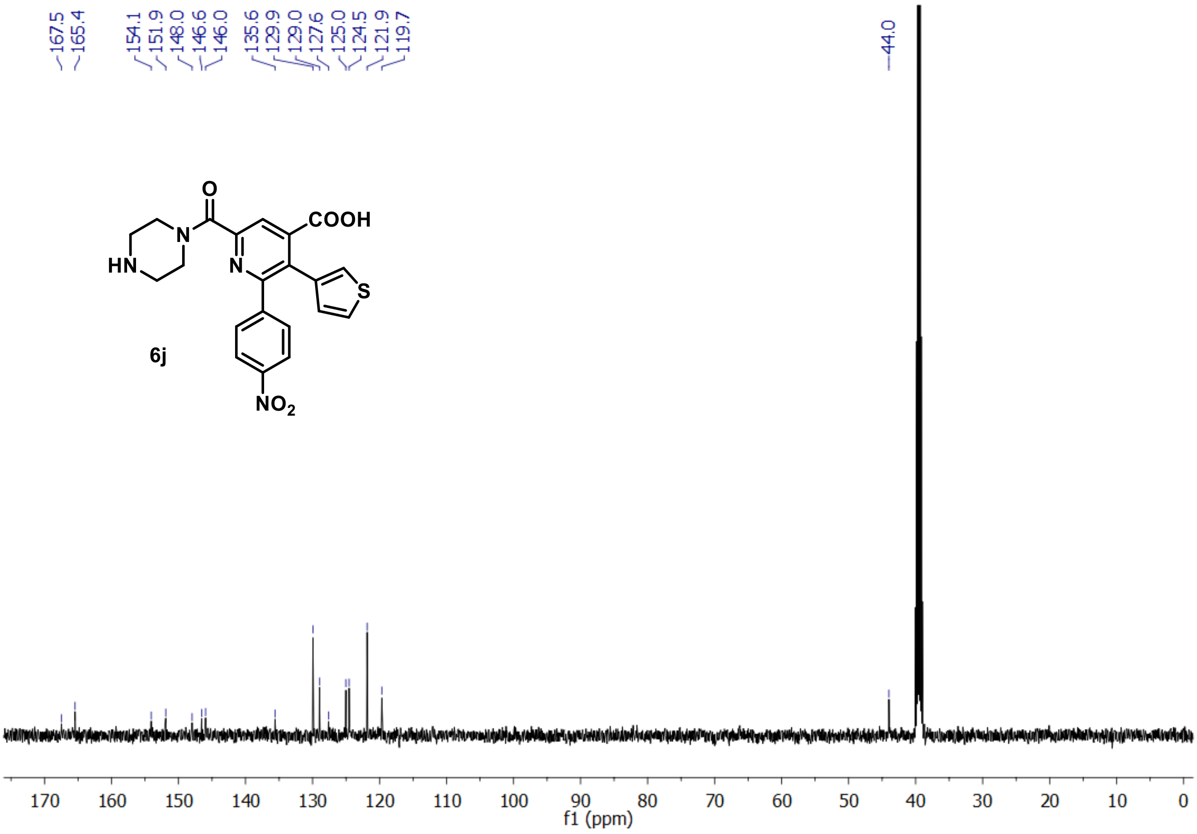

3-(3-methoxyphenyl)-2-(4-nitrophenyl)-6-(piperazine-1-carbonyl)-N-propylisonicotinamide (6k)

^{1}H NMR (500Mhz; DMSO-d_6) spectrum of **6k**

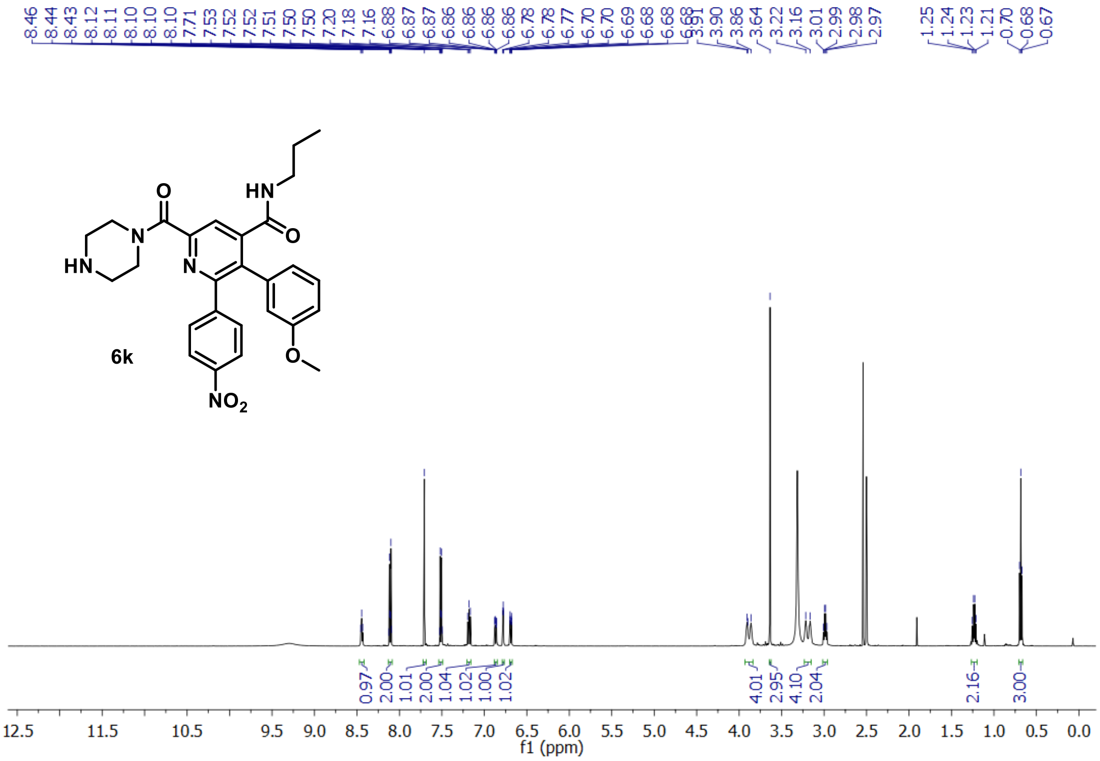

^{13}C{^{1}H} NMR (126 MHz; DMSO-d_6) spectrum of **6k**

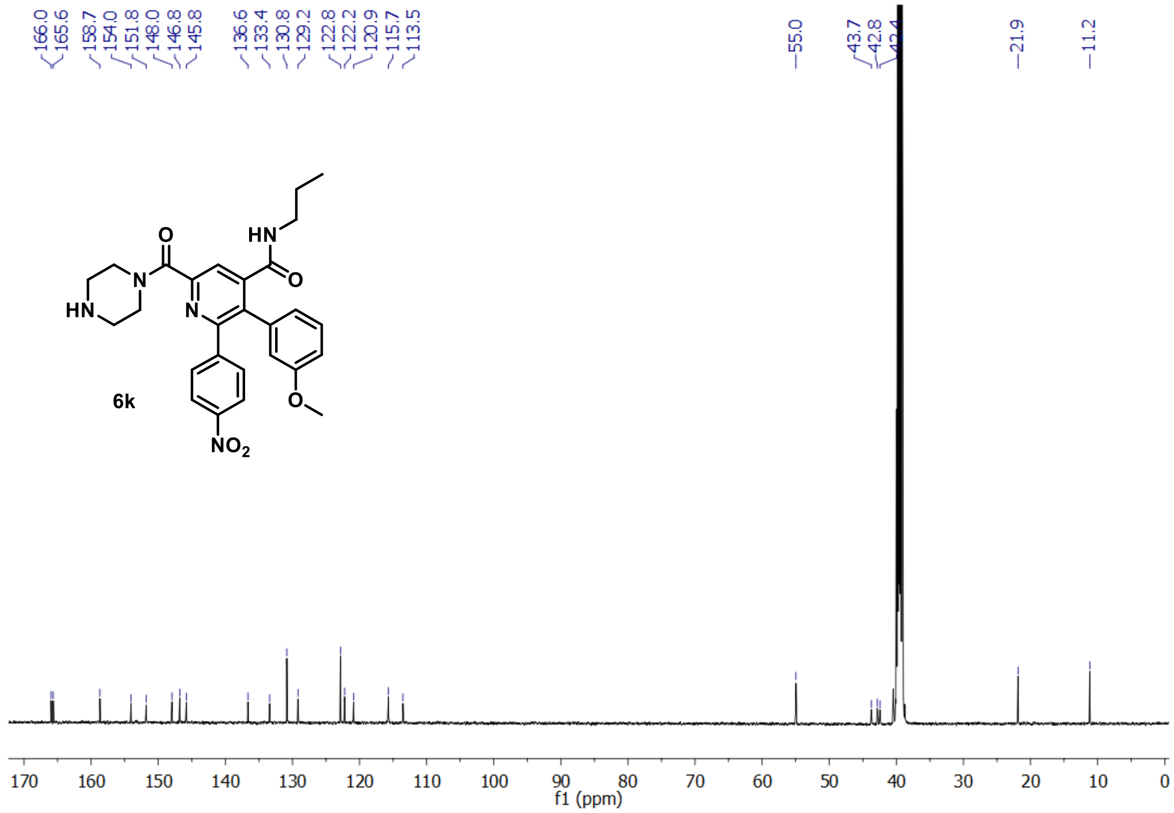

3-(4-methoxyphenyl)-2-(4-nitrophenyl)-6-(propylcarbamoyl)isonicotinic acid (6l)

^{1}H NMR (500Mhz; DMSO-d_6) spectrum of **6l**

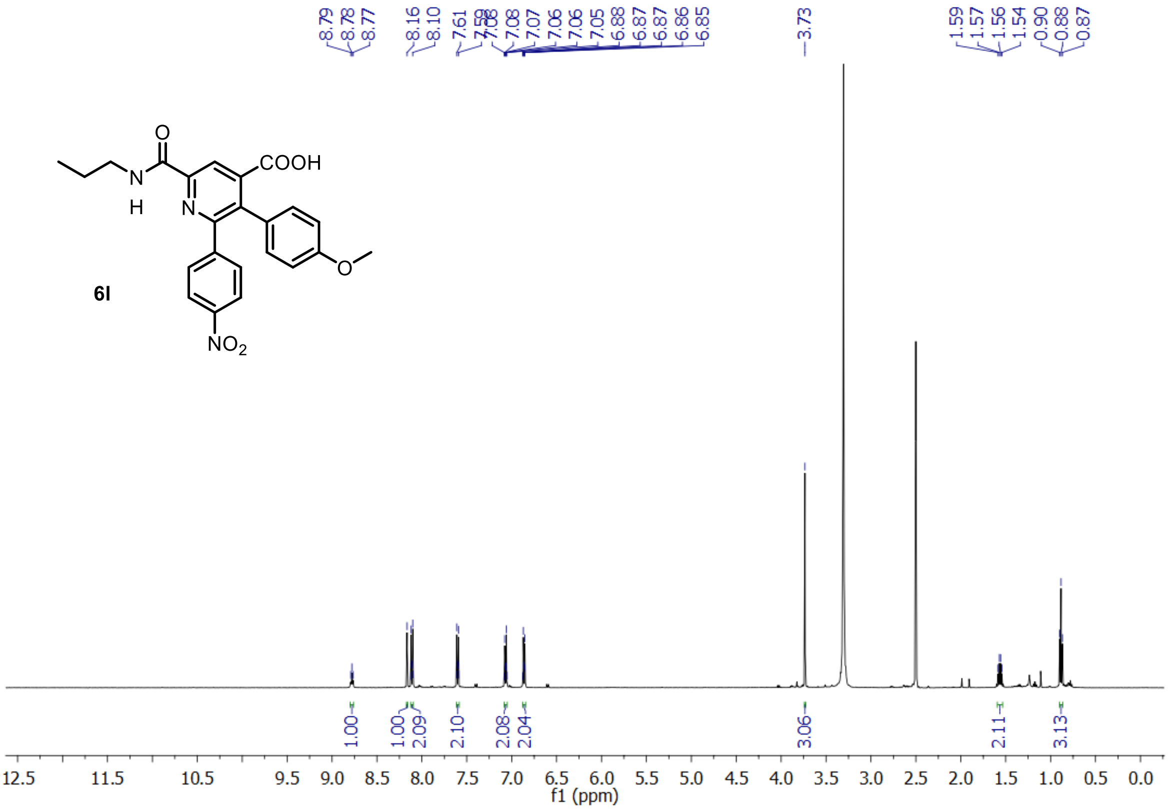

^{13}C{^{1}H} NMR (126 MHz; DMSO-d_6) spectrum of **6l**

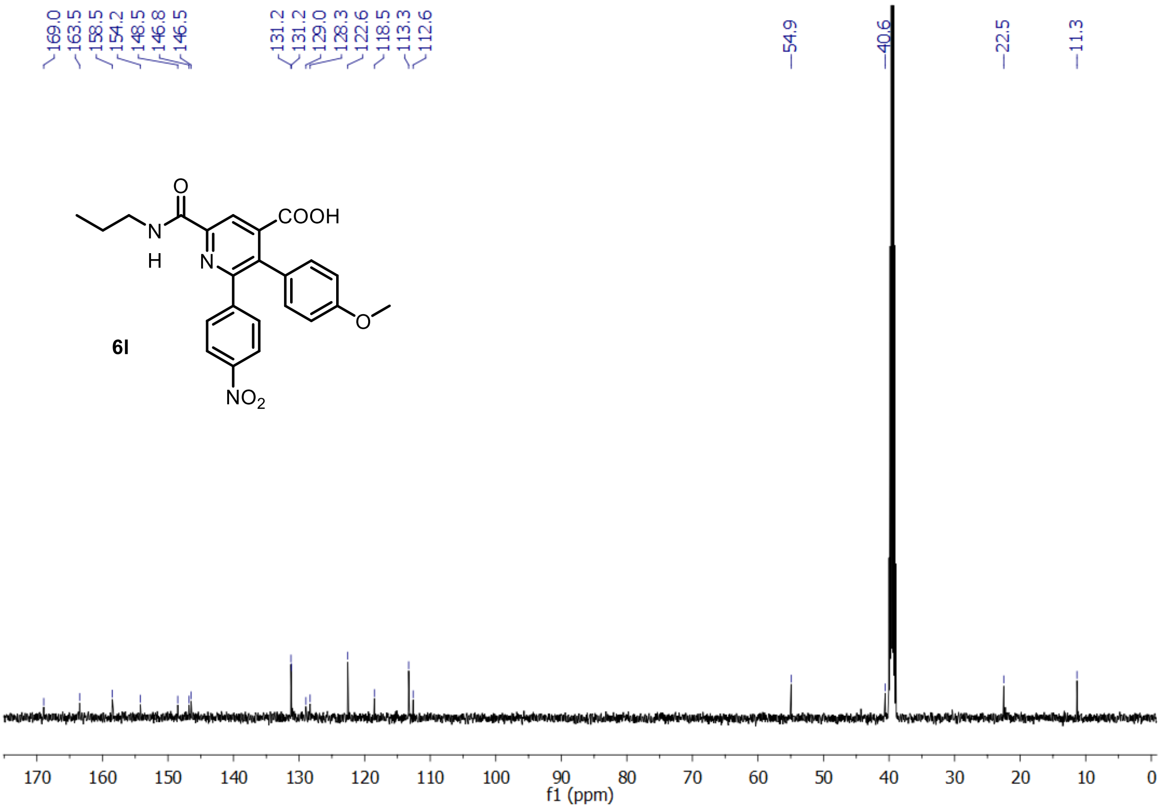

3-(6-(4-nitrophenyl)-3-oxo-5-(*p*-tolyl)-3,4-dihydropyrazin-2-yl)propanoic acid (7a)

^{1}H NMR (500Mhz; DMSO-d_6) spectrum of **7a**

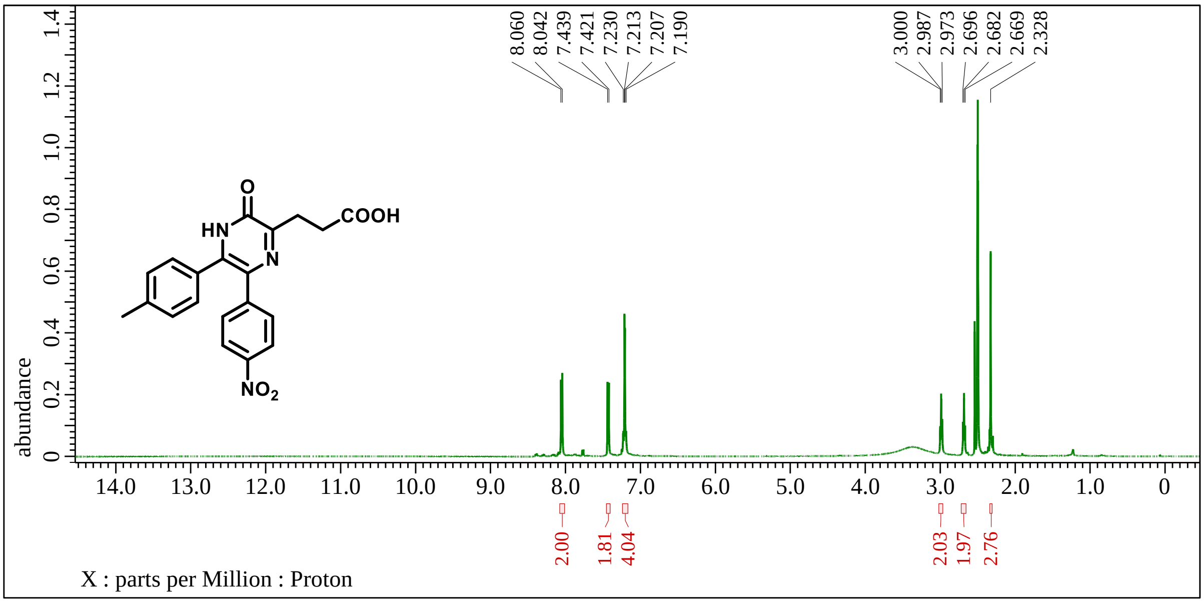

$^{13}C\{^{1}H\}$ NMR (126 MHz; DMSO-d_6) spectrum of **7a**

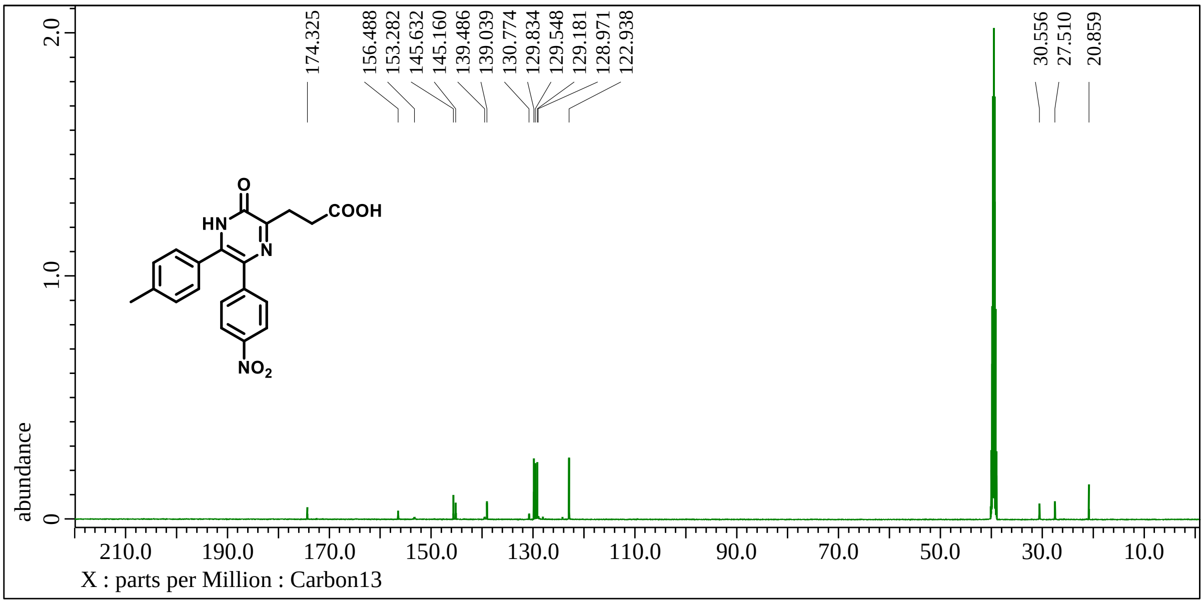

Methyl 3-(5-(4-methoxyphenyl)-6-(4-nitrophenyl)-3-oxo-3,4-dihydropyrazin-2-yl)propanoate (7b)

^{1}H NMR (500Mhz; CHLOROFORM-D) spectrum of **7b**

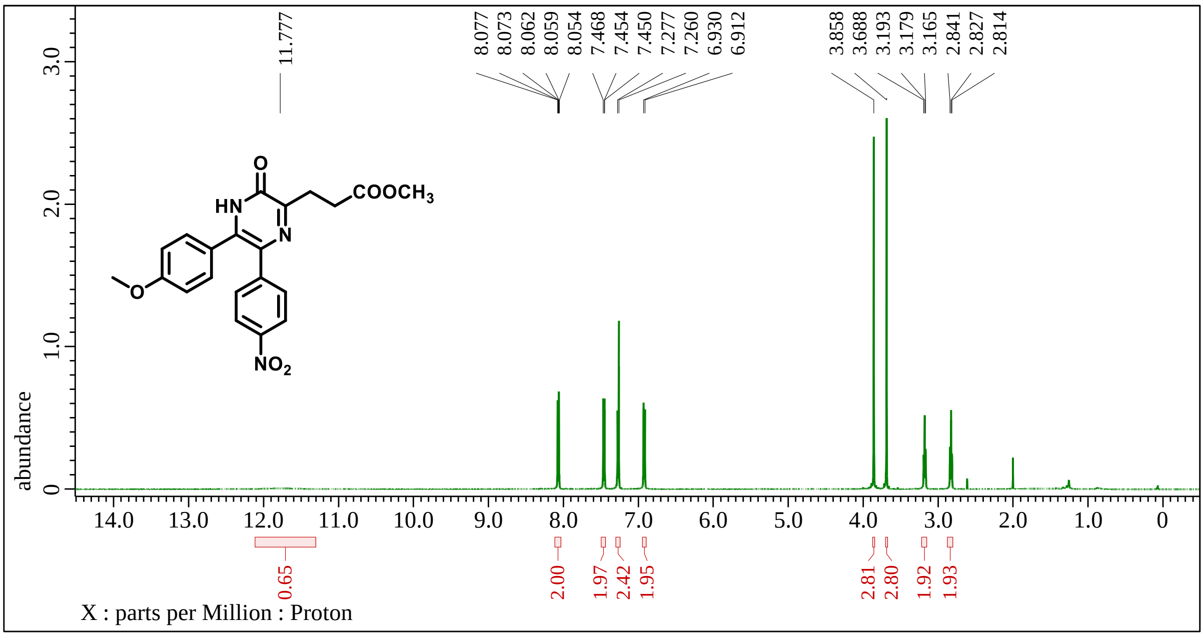

^{13}C{^{1}H} NMR (126 MHz; CHLOROFORM-D) spectrum of **7b**

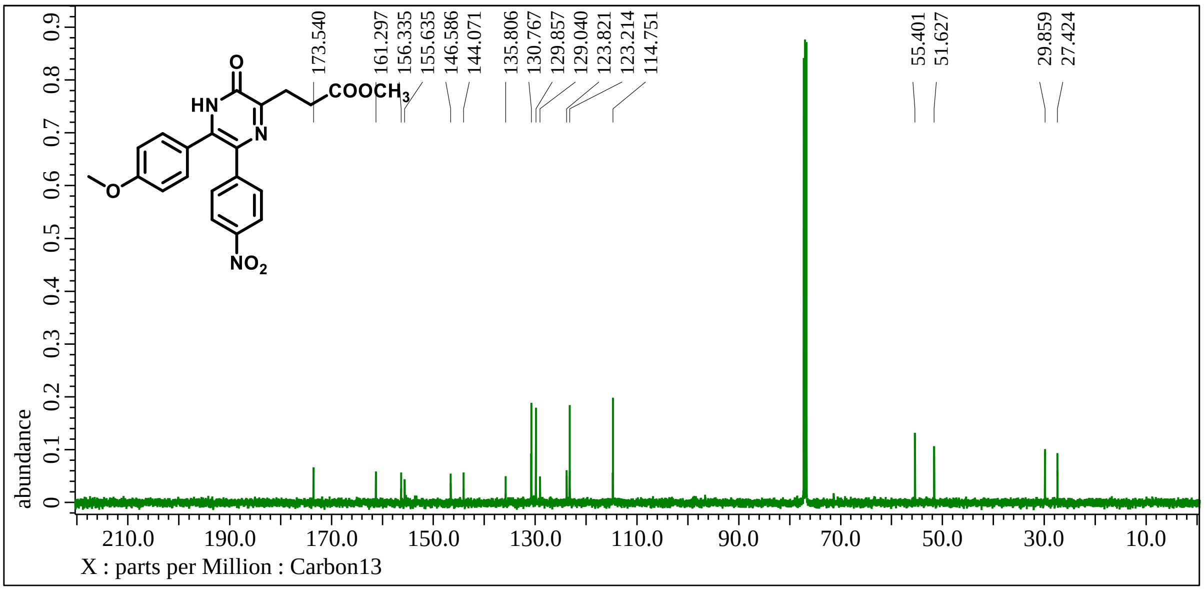

5-(4-nitrophenyl)-6-(*p*-tolyl)pyrazin-2(1*H*)-one (7c)

^{1}H NMR (500Mhz; DMSO-d_6) spectrum of **7c**

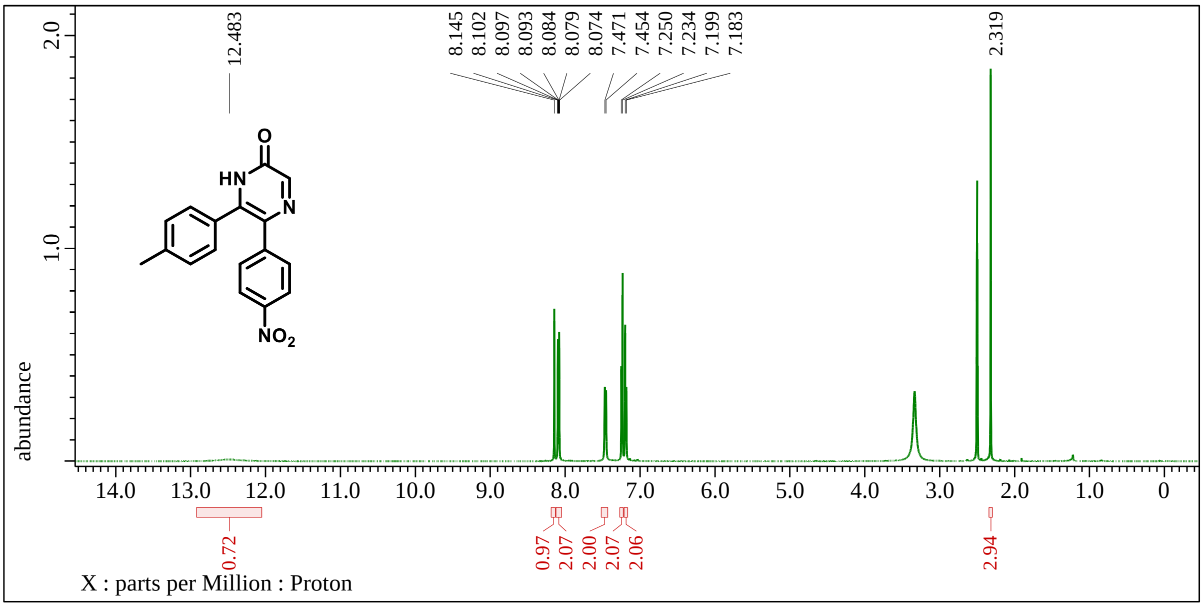

^{13}C{^{1}H} NMR (126 MHz; DMSO-d_6) spectrum of **7c**

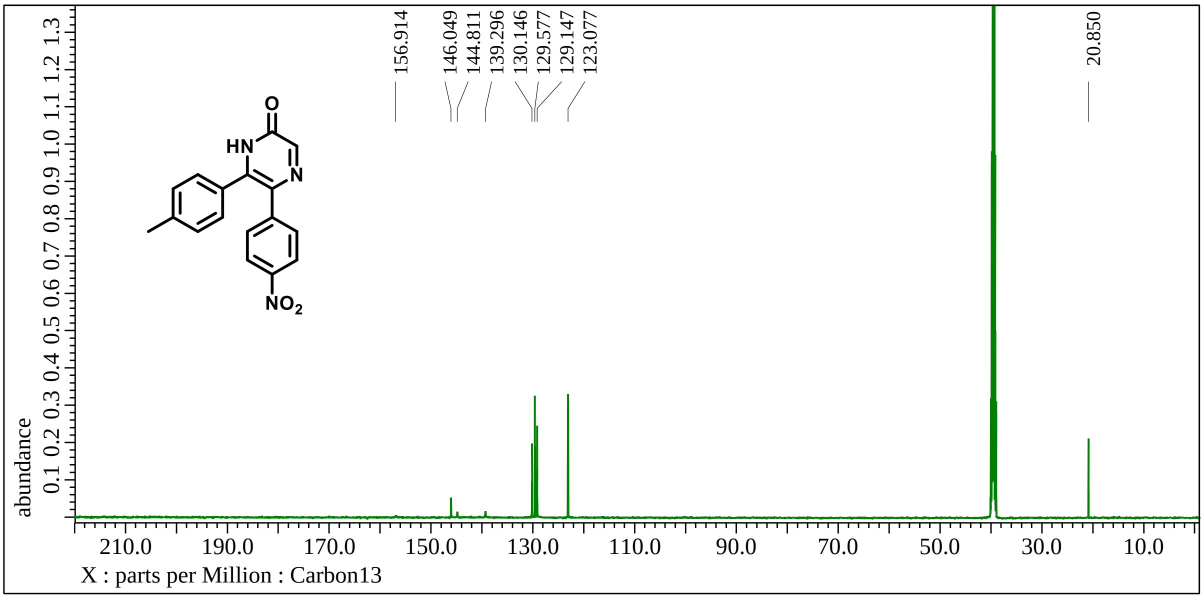

3-methyl-5-(4-nitrophenyl)-6-(*p*-tolyl)pyrazin-2(1*H*)-one (7d)

^{1}H NMR (500Mhz; CHLOROFORM-D) spectrum of **7d**

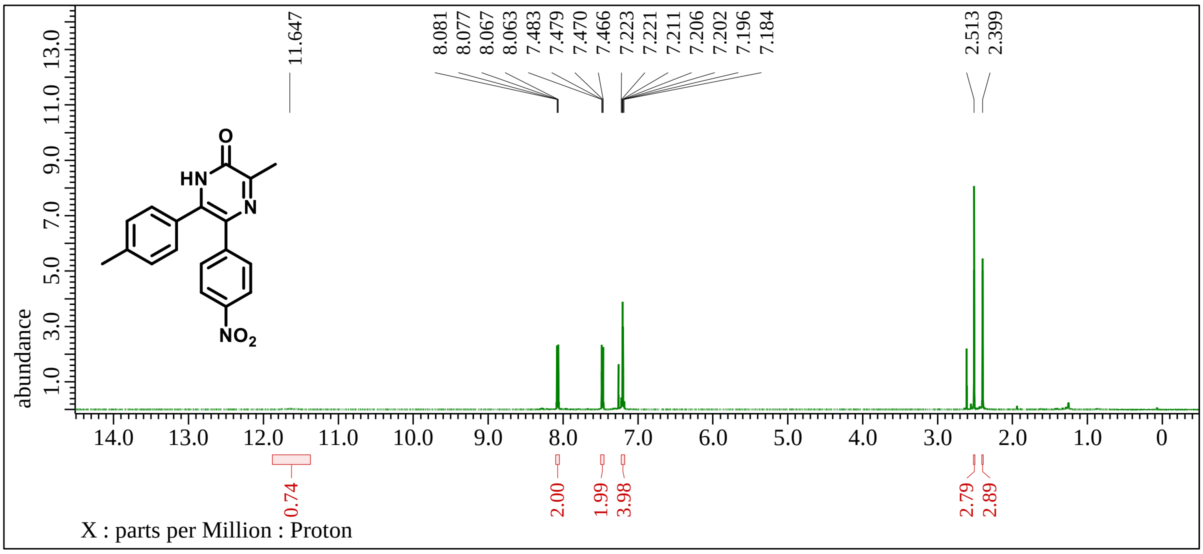

^{13}C{^{1}H} NMR (126 MHz; CHLOROFORM-D) spectrum of **7d**

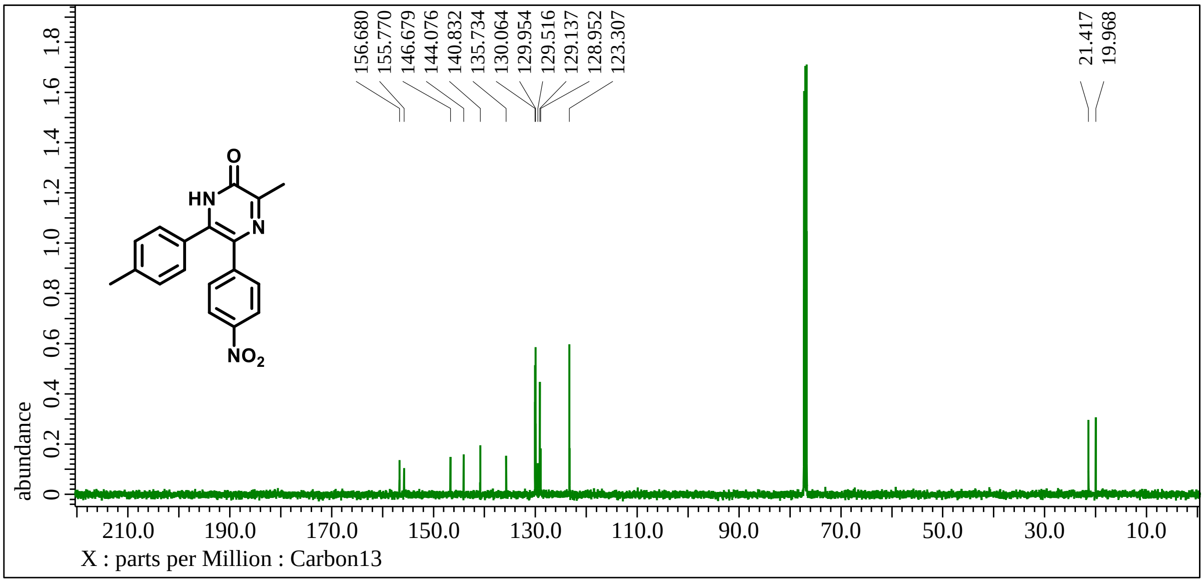

3-isobutyl-5-(4-nitrophenyl)-6-(*p*-tolyl)pyrazin-2(1*H*)-one (7e)

^{1}H NMR (500Mhz; CHLOROFORM-D) spectrum of **7e**

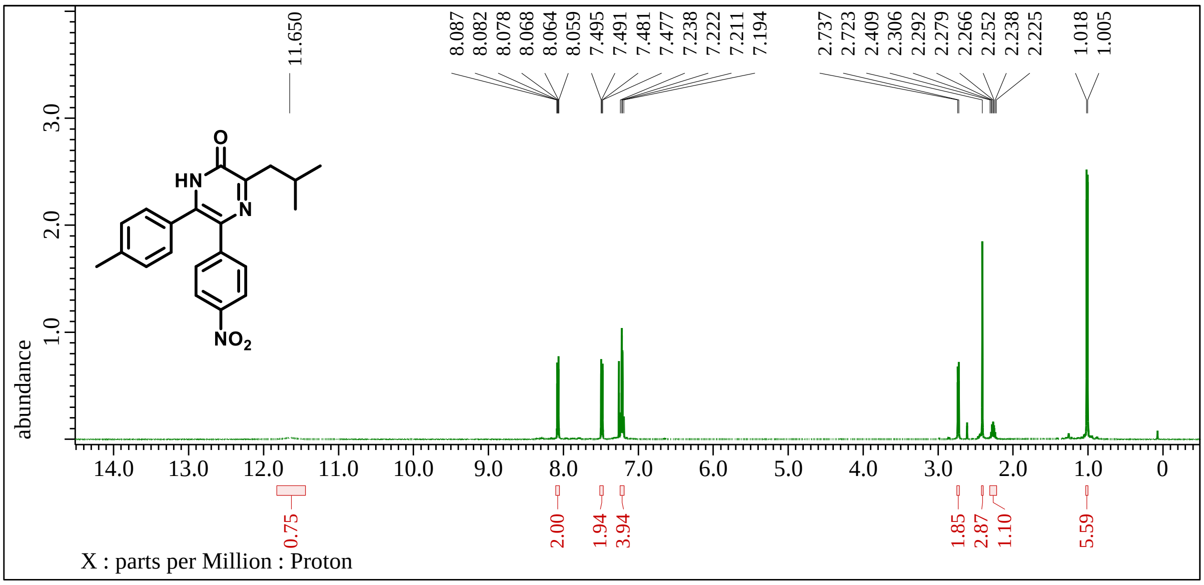

^{13}C{^{1}H} NMR (126 MHz; CHLOROFORM-D) spectrum of **7e**

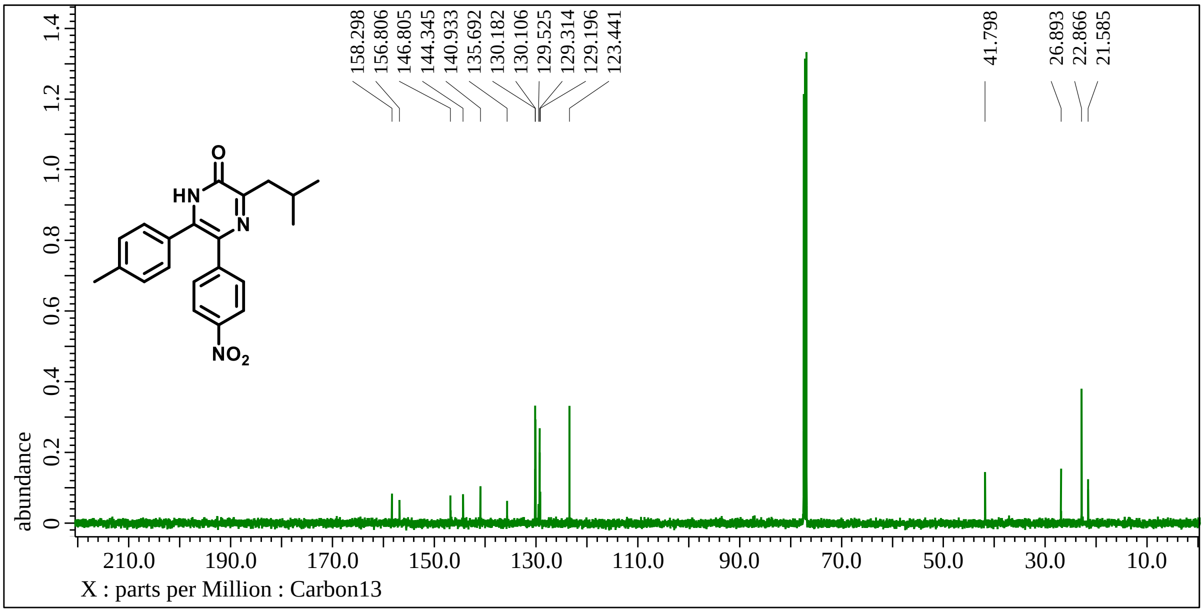

3-benzyl-5-(4-nitrophenyl)-6-(*p*-tolyl)pyrazin-2(1*H*)-one (7f)

^{1}H NMR (500Mhz; CHLOROFORM-D) spectrum of **7f**

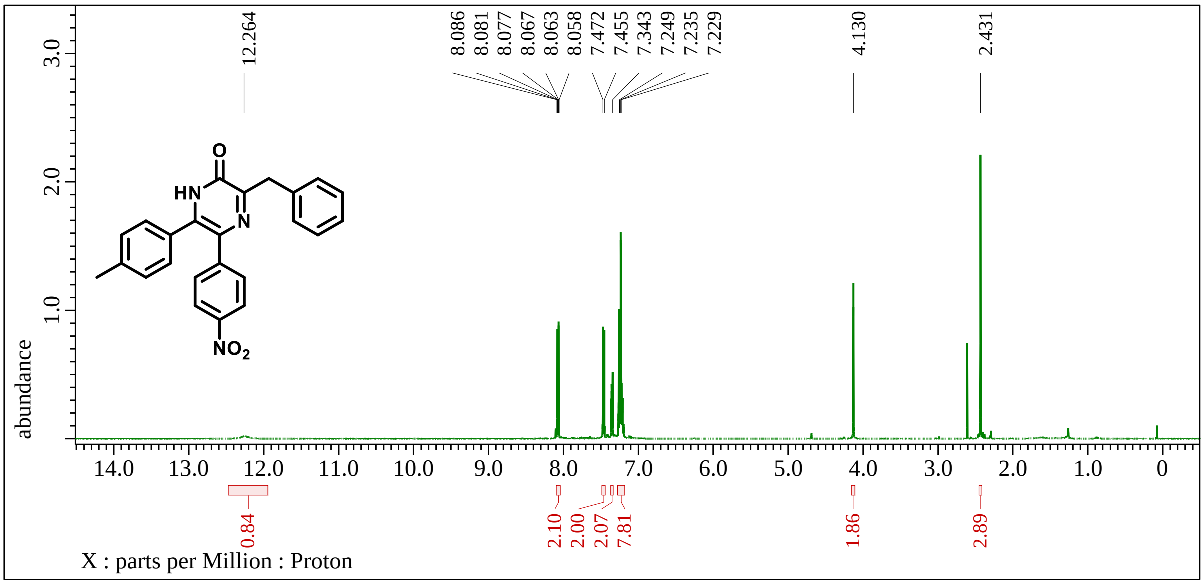

$^{13}C\{^{1}H\}$ NMR (126 MHz; CHLOROFORM-D) spectrum of **7f**

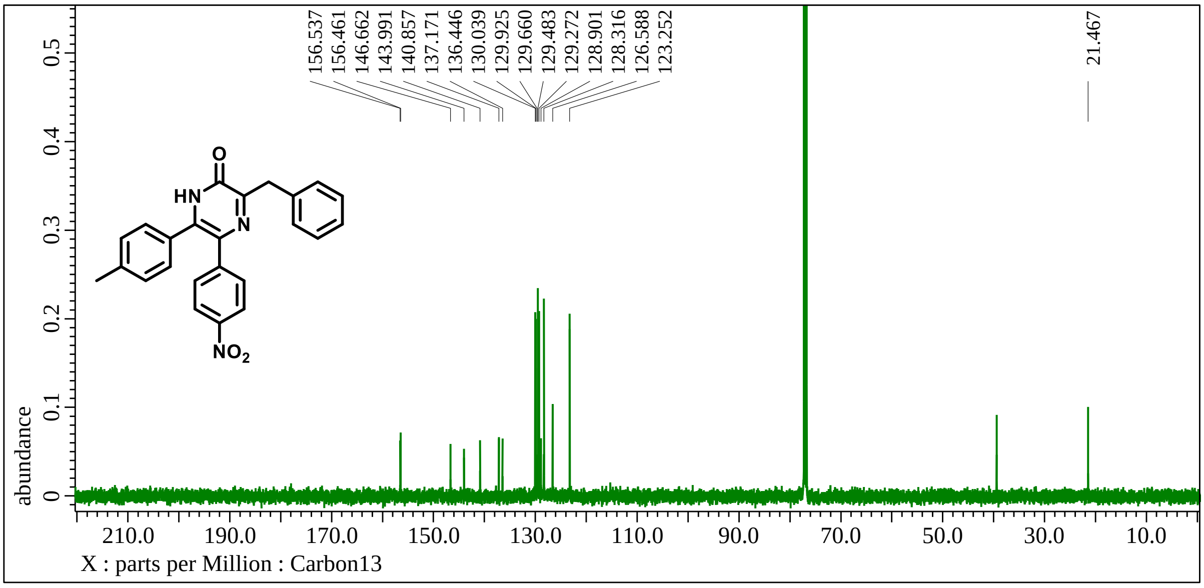

3-(4-hydroxybenzyl)-5-(4-nitrophenyl)-6-(*p*-tolyl)pyrazin-2(1*H*)-one (7g)

^1H NMR (500Mhz; DMSO-*d*$_6$) spectrum of **7g**

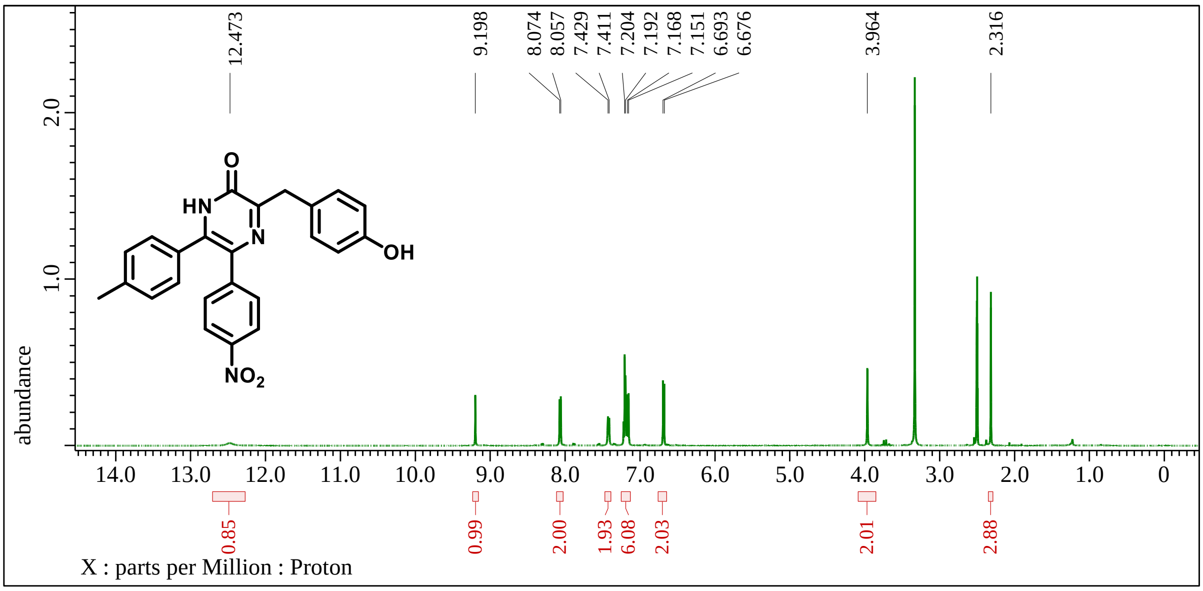

$^{13}C\{^1H\}$ NMR (126 MHz; DMSO-*d*$_6$) spectrum of **7g**

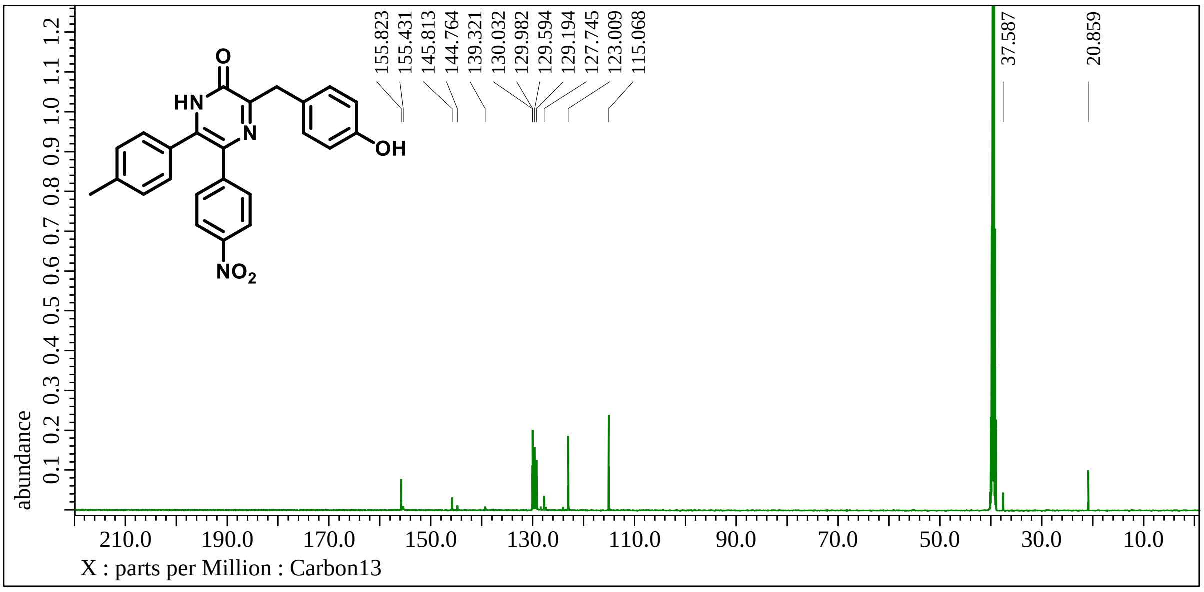

3-(2-(methylthio)ethyl)-5-(4-nitrophenyl)-6-(*p*-tolyl)pyrazin-2(1*H*)-one (7h)

^{1}H NMR (500Mhz; DMSO-d_6) spectrum of **7h**

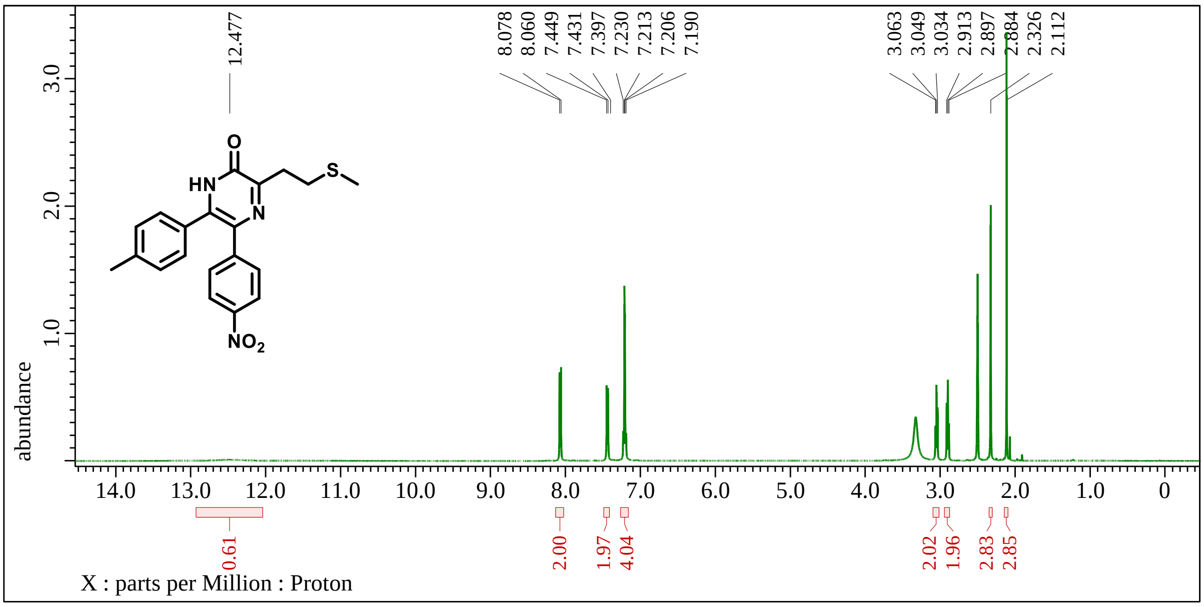

$^{13}C\{^{1}H\}$ NMR (126 MHz; DMSO-d_6) spectrum of **7h**

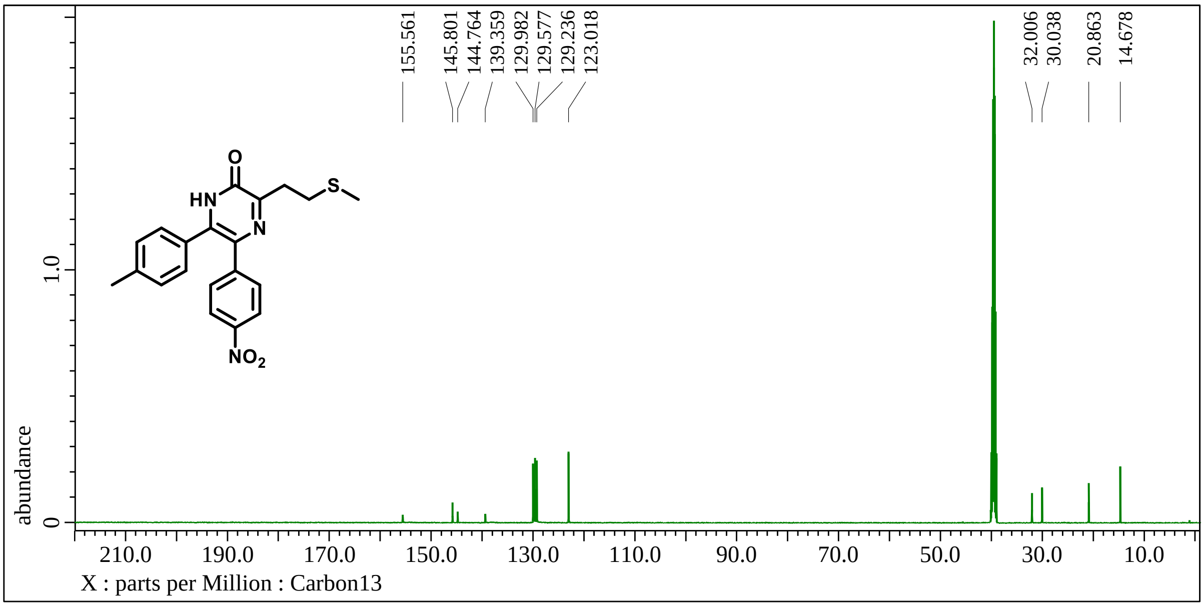

3-(6-(4-nitrophenyl)-3-oxo-5-(*p*-tolyl)-3,4-dihydropyrazin-2-yl)propanamide (7i)

^{1}H NMR (500Mhz; DMSO-*d₆*) spectrum of **7i**

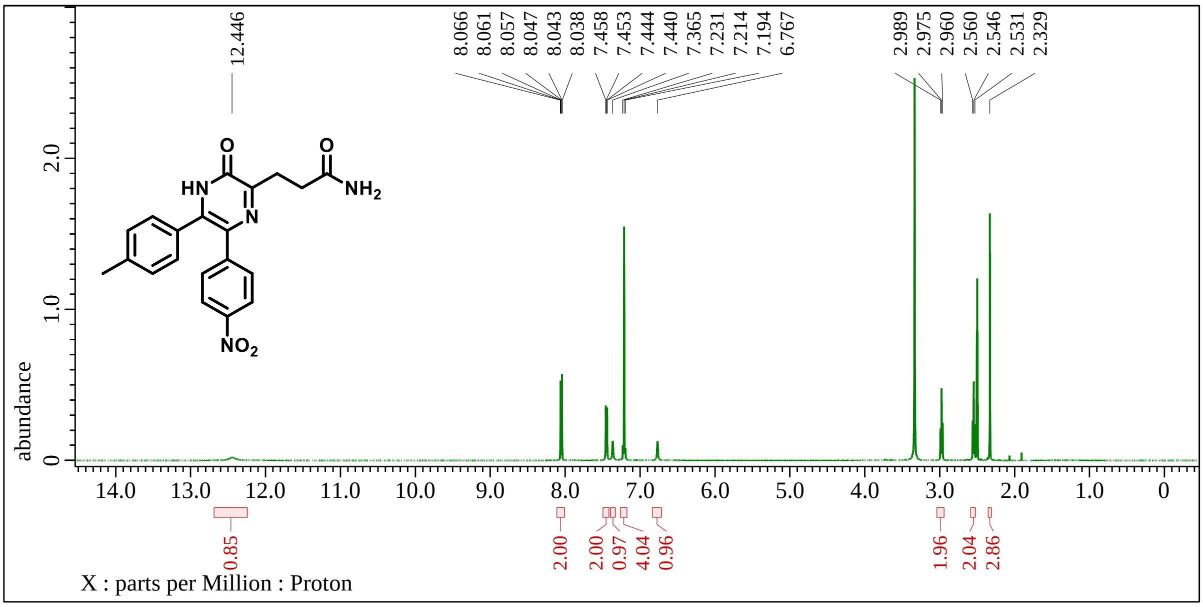

^{13}C{^{1}H} NMR (126 MHz; DMSO-*d₆*) spectrum of **7i**

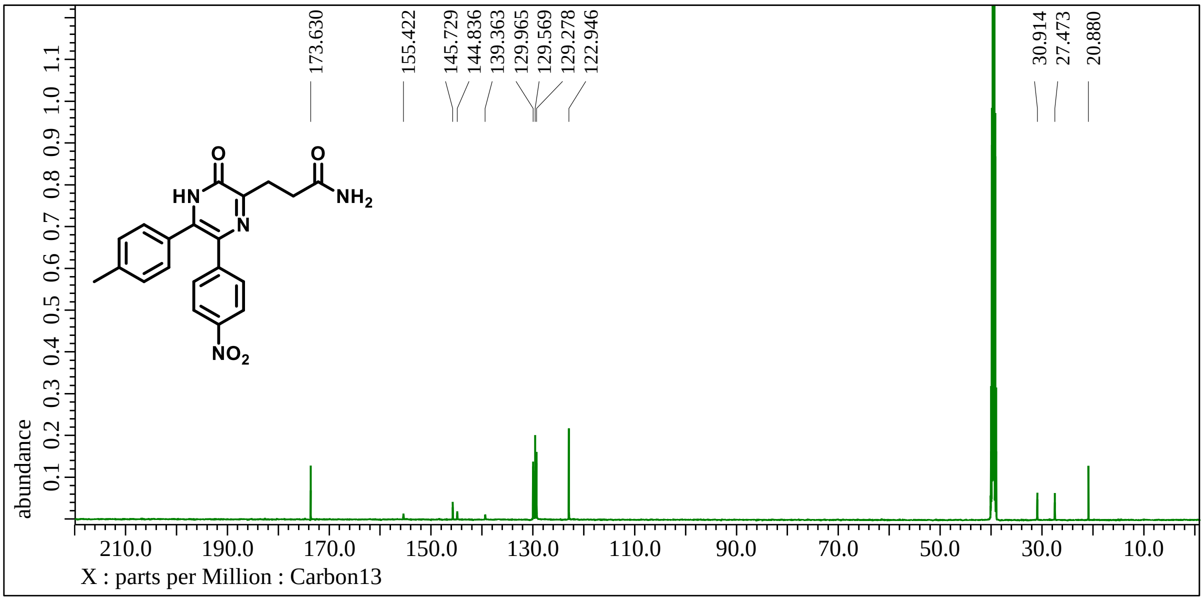

1-(3-(6-(4-nitrophenyl)-3-oxo-5-(*p*-tolyl)-3,4-dihydropyrazin-2-yl)propyl)guanidine (7j)

^{1}H NMR (500Mhz; DMSO-d_6) spectrum of **7j**

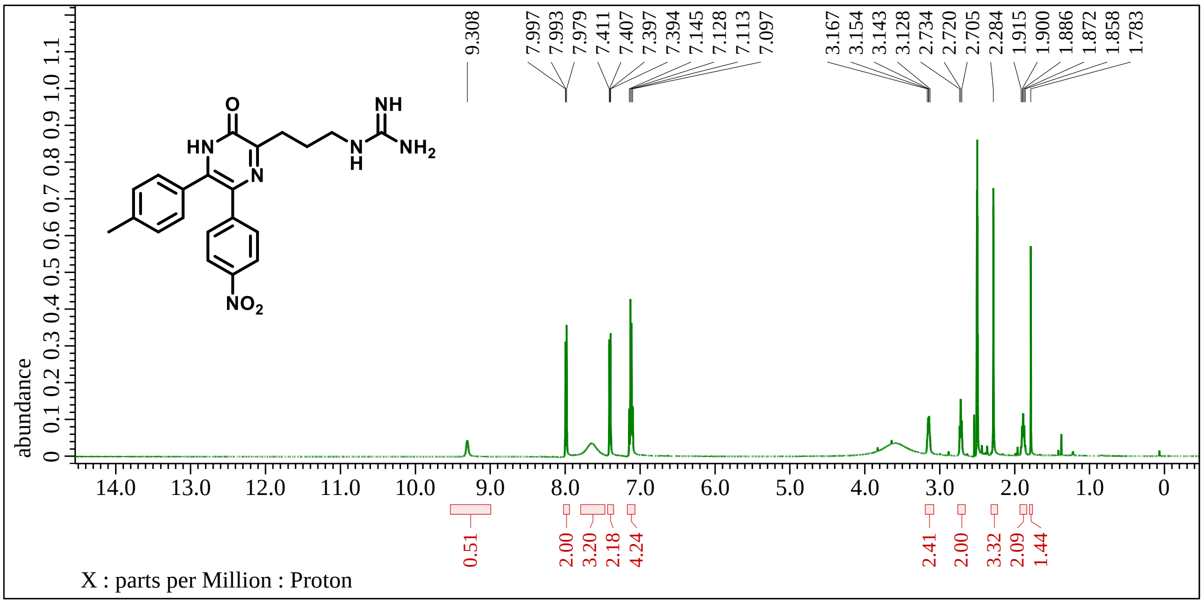

$^{13}C\{^{1}H\}$ NMR (126 MHz; DMSO-d_6) spectrum of **7j**

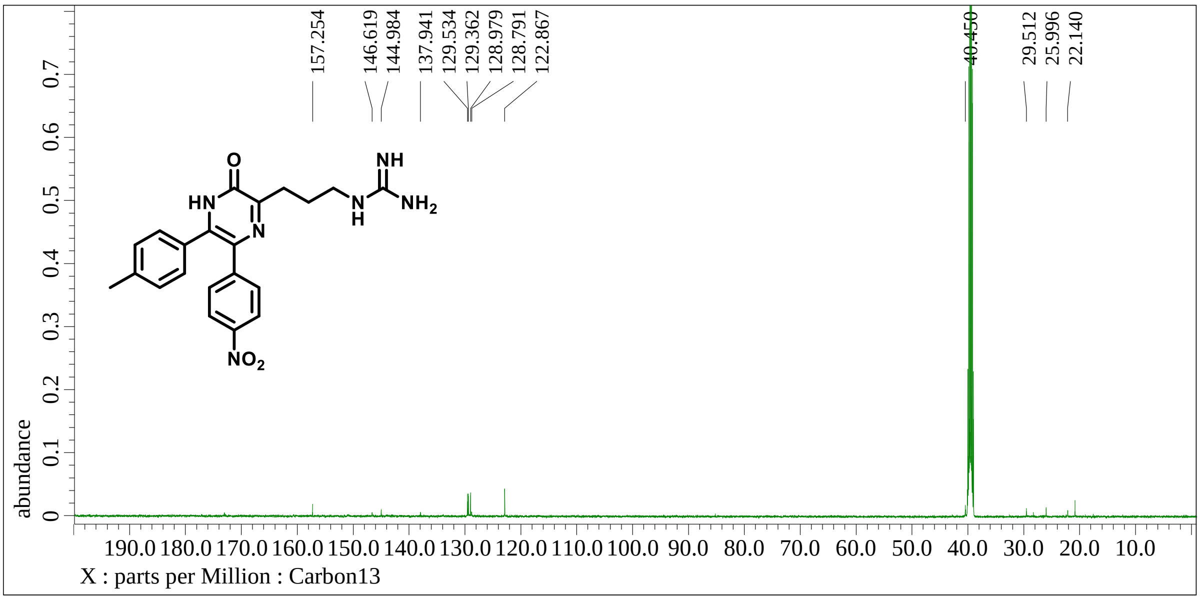

6-(4-amino-3,5-dichlorophenyl)-3-methyl-5-(4-nitrophenyl)pyrazin-2(1*H*)-one (7k)

^{1}H NMR (500Mhz; CHLOROFORM) spectrum of **7k**

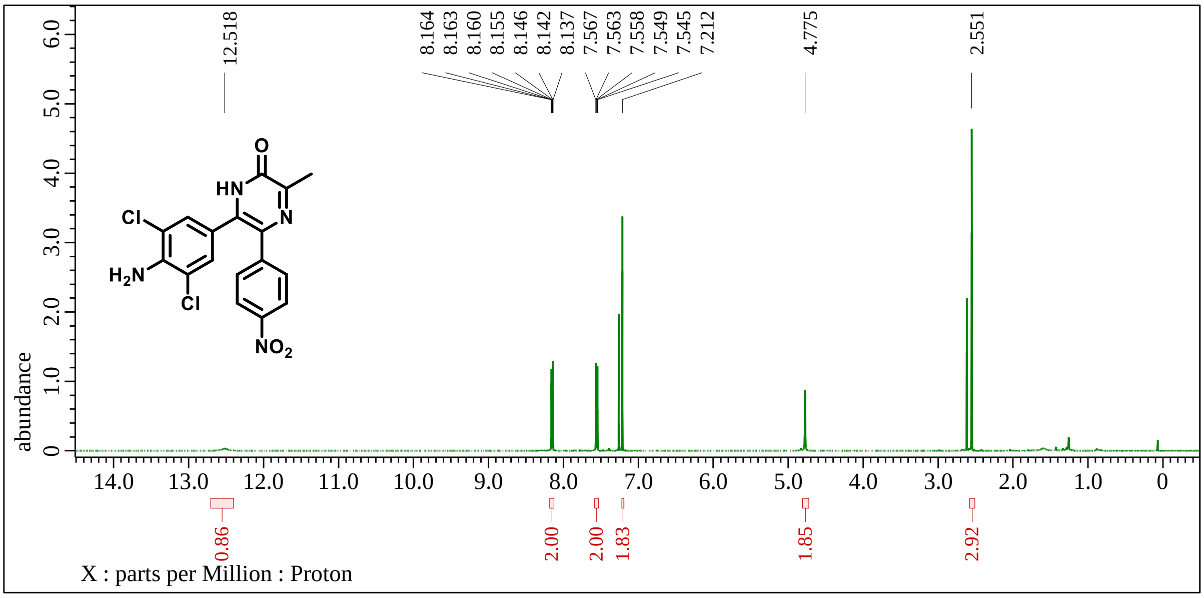

^{13}C{^{1}H} NMR (126 MHz; CHLOROFORM) spectrum of **7k**

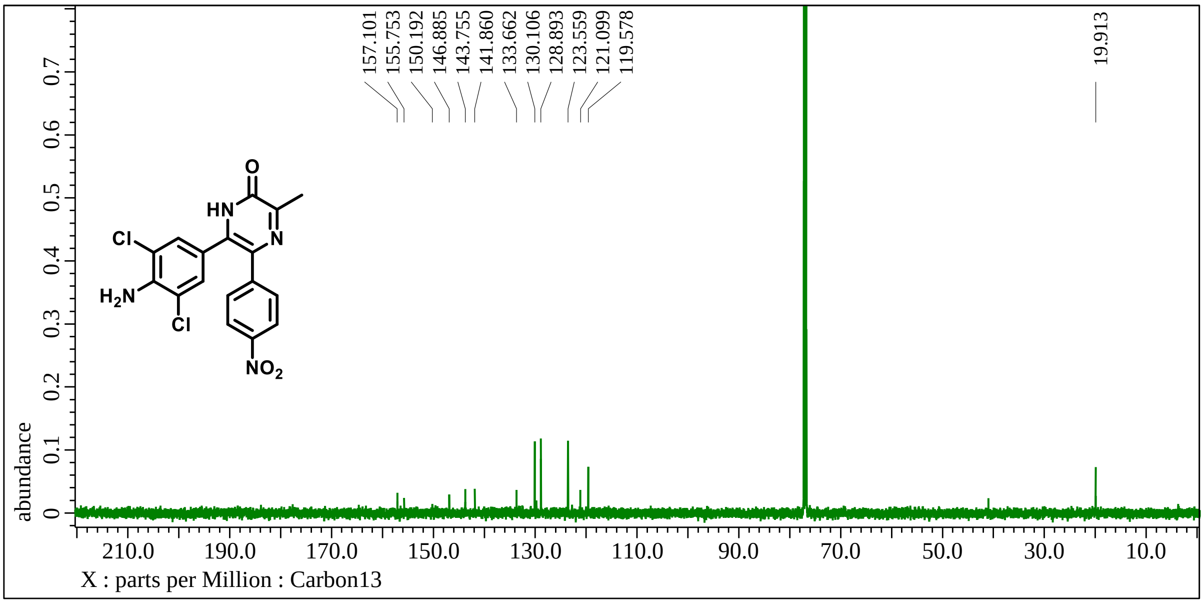

3-((1*H*-indol-3-yl)methyl)-6-(4-methoxyphenyl)-5-(4-nitrophenyl)pyrazin-2(1*H*)-one (7l)

^{1}H NMR (500Mhz; DMSO-d_6) spectrum of **7l**

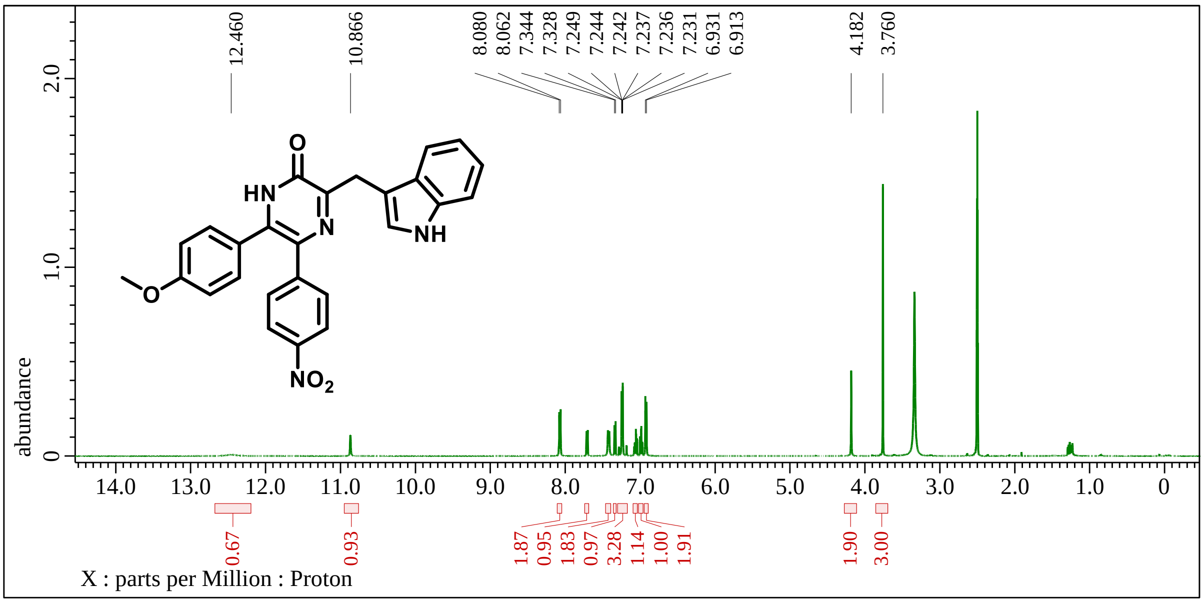

^{13}C{^{1}H} NMR (126 MHz; DMSO-d_6) spectrum of **7l**

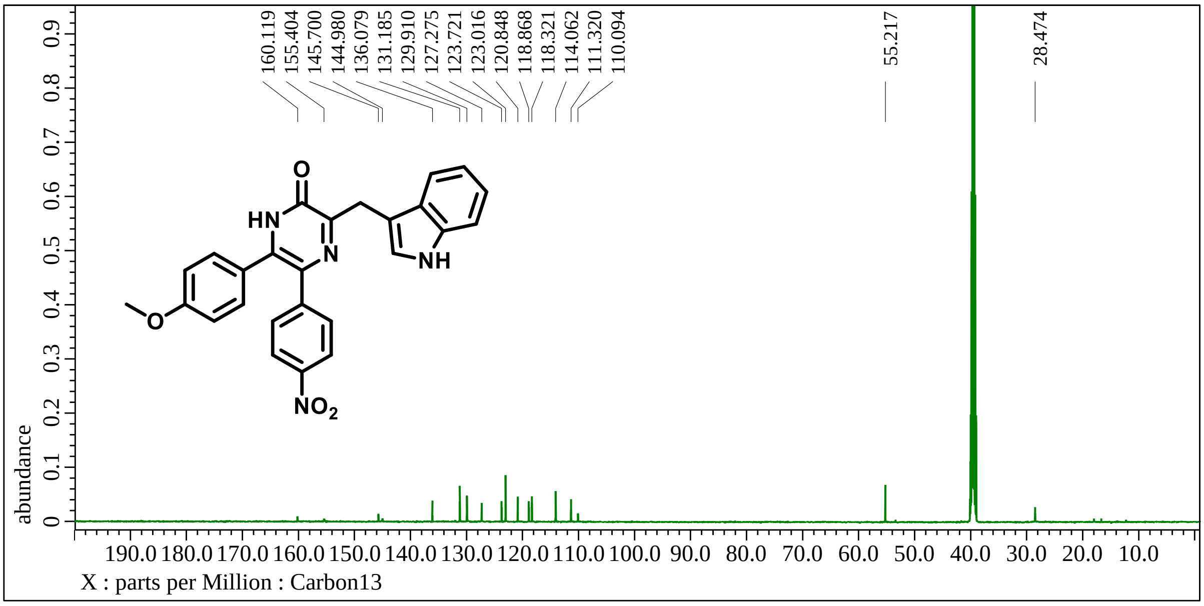

High-resolution mass spectra (HRMS) for the final compounds

6-carbamoyl-2-(4-nitrophenyl)-3-(*p*-tolyl)isonicotinic acid (6a)

ESI+

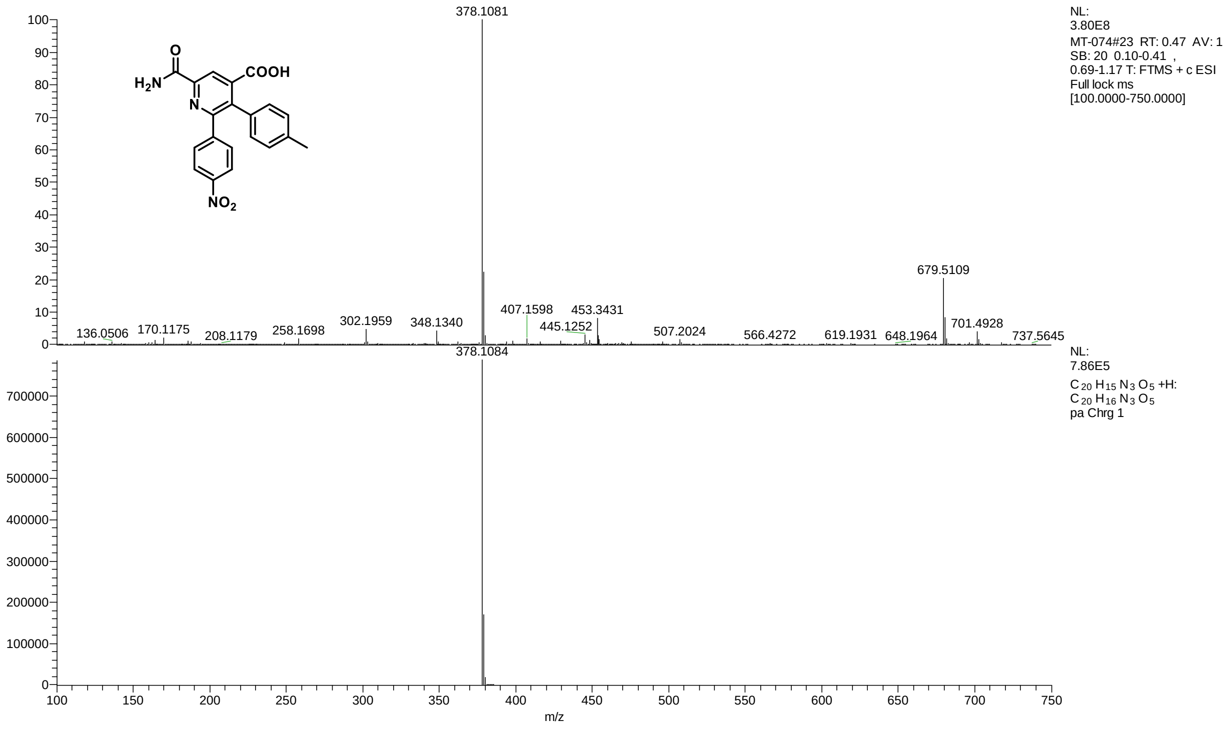

ESI-

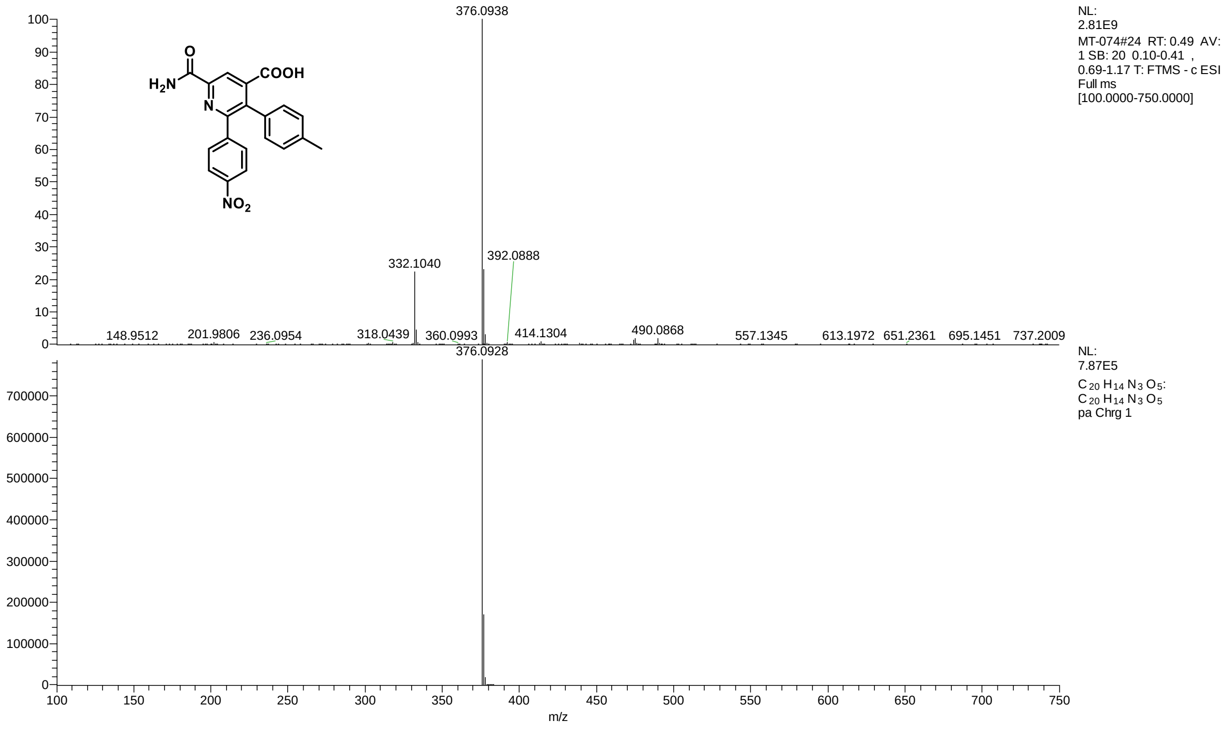

6-carbamoyl-3-(4-methoxyphenyl)-2-(4-nitrophenyl)isonicotinic acid (6b)

ESI+

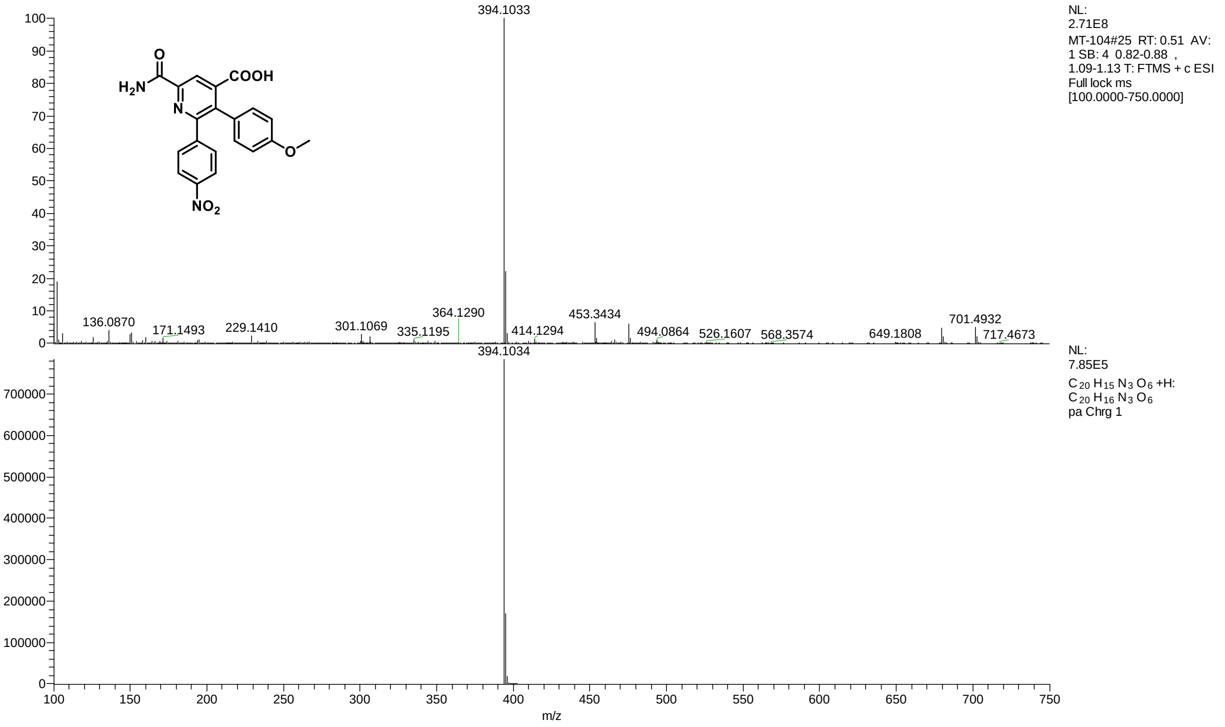

ESI-

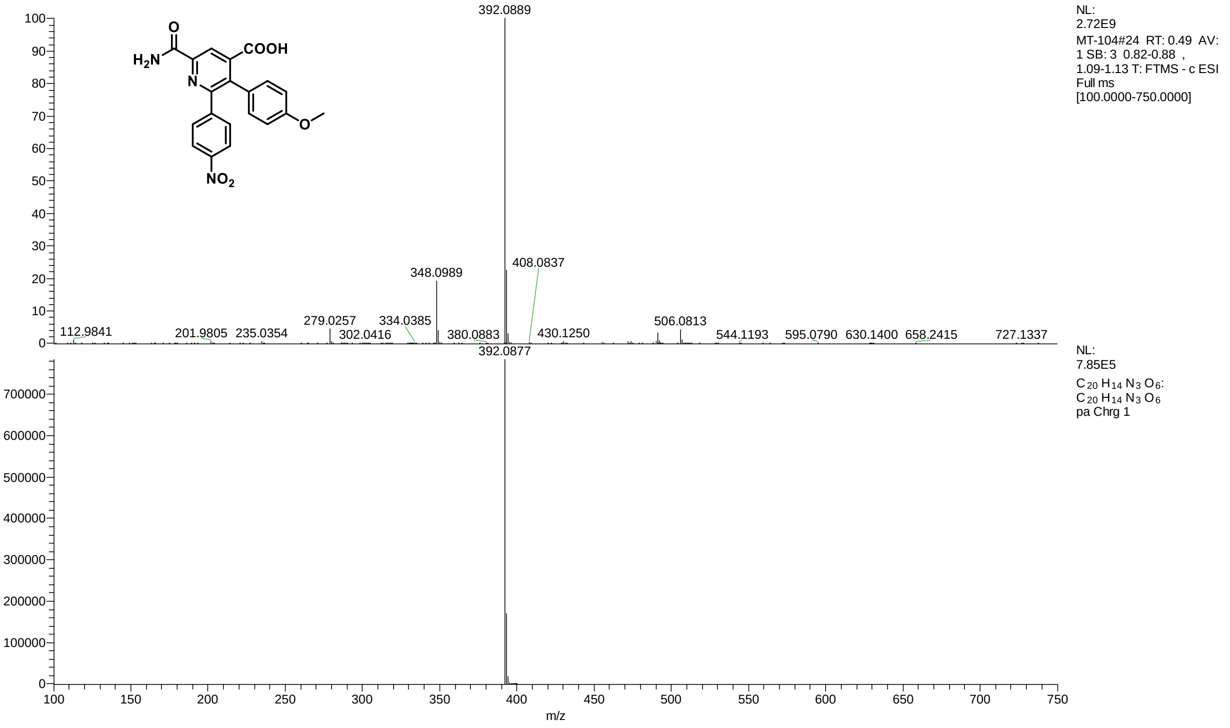

6-(3-carbamoylpyrrolidine-1-carbonyl)-2-(4-nitrophenyl)-3-(p-tolyl)isonicotinic acid (6c)

ESI+

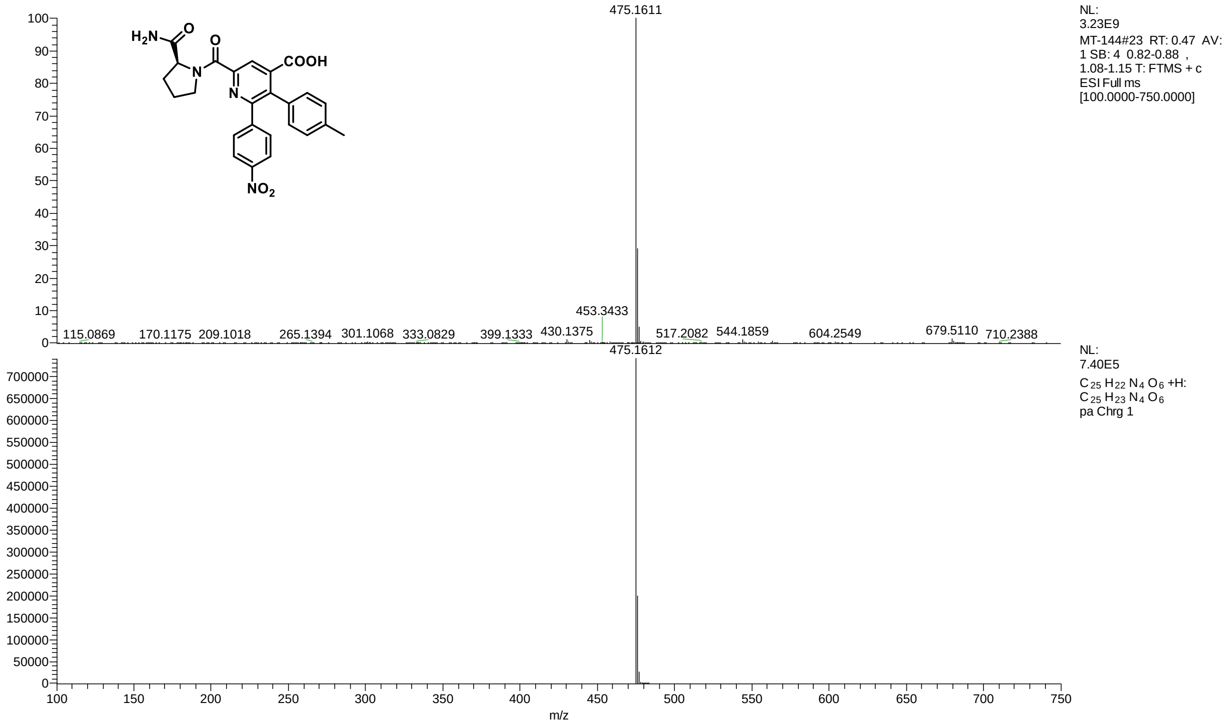

ESI-

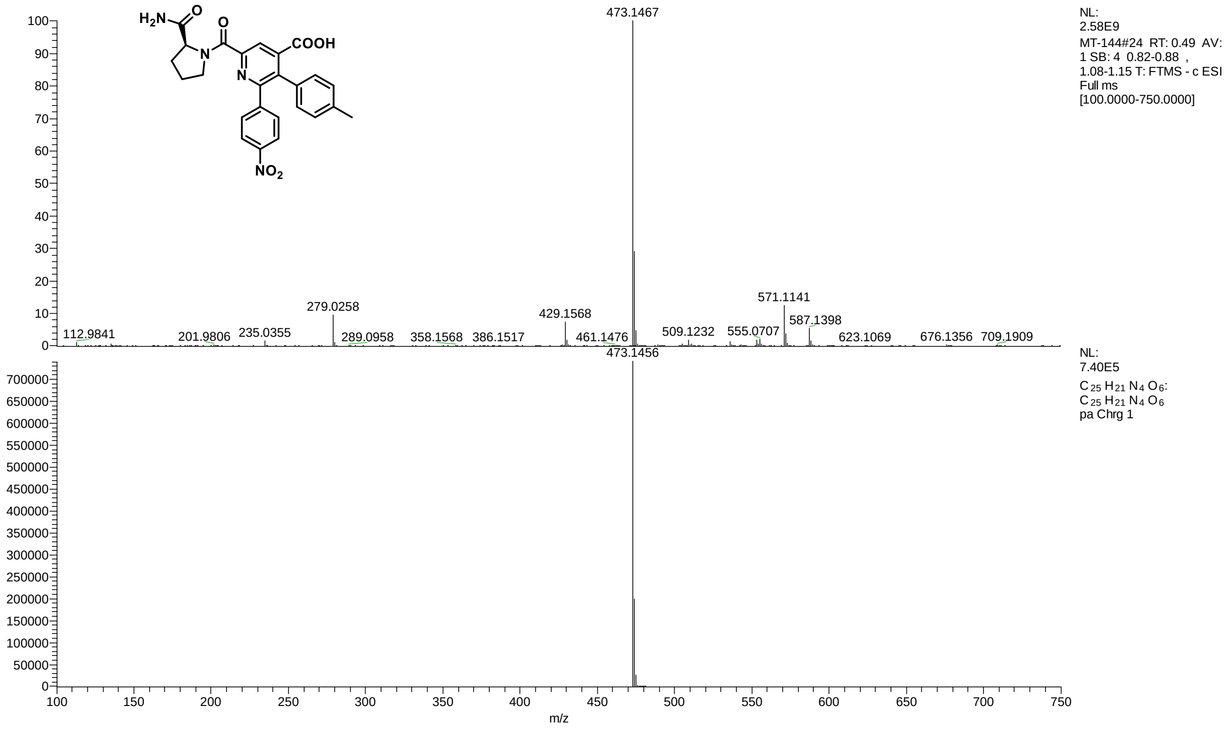

3-(4-fluorophenyl)-2-(4-nitrophenyl)-6-(piperazine-1-carbonyl)isonicotinic acid (6d)

ESI+

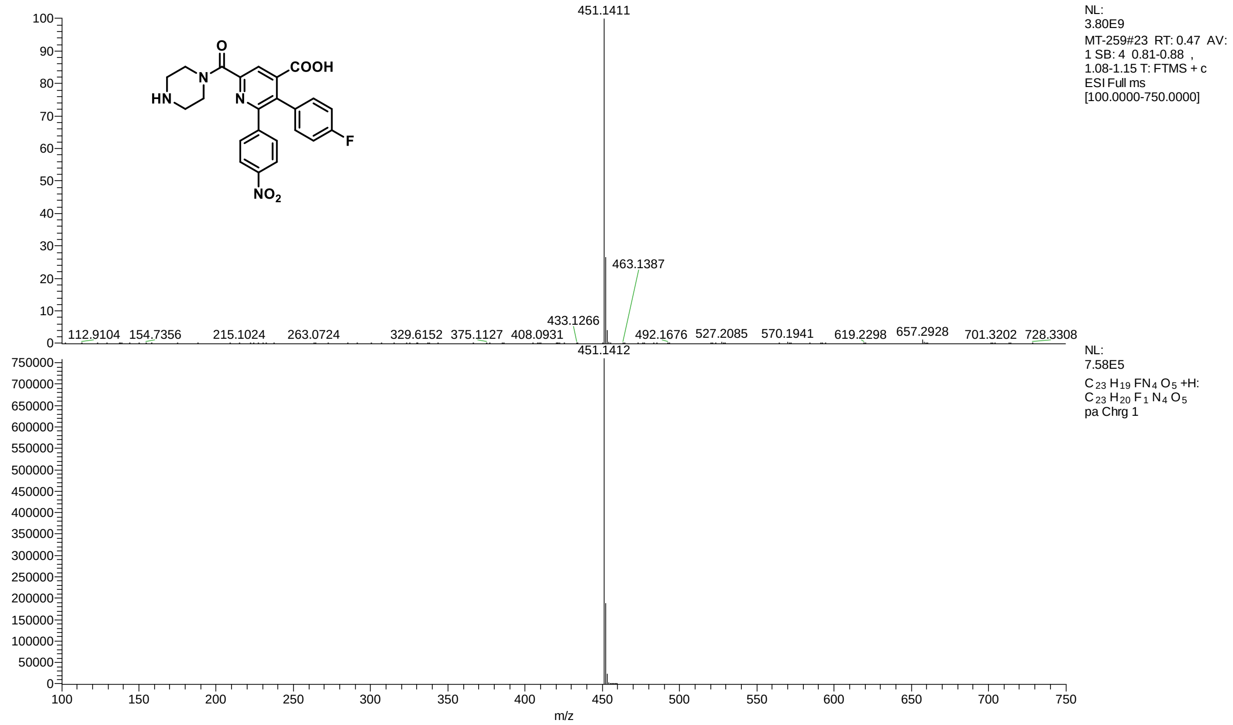

ESI-

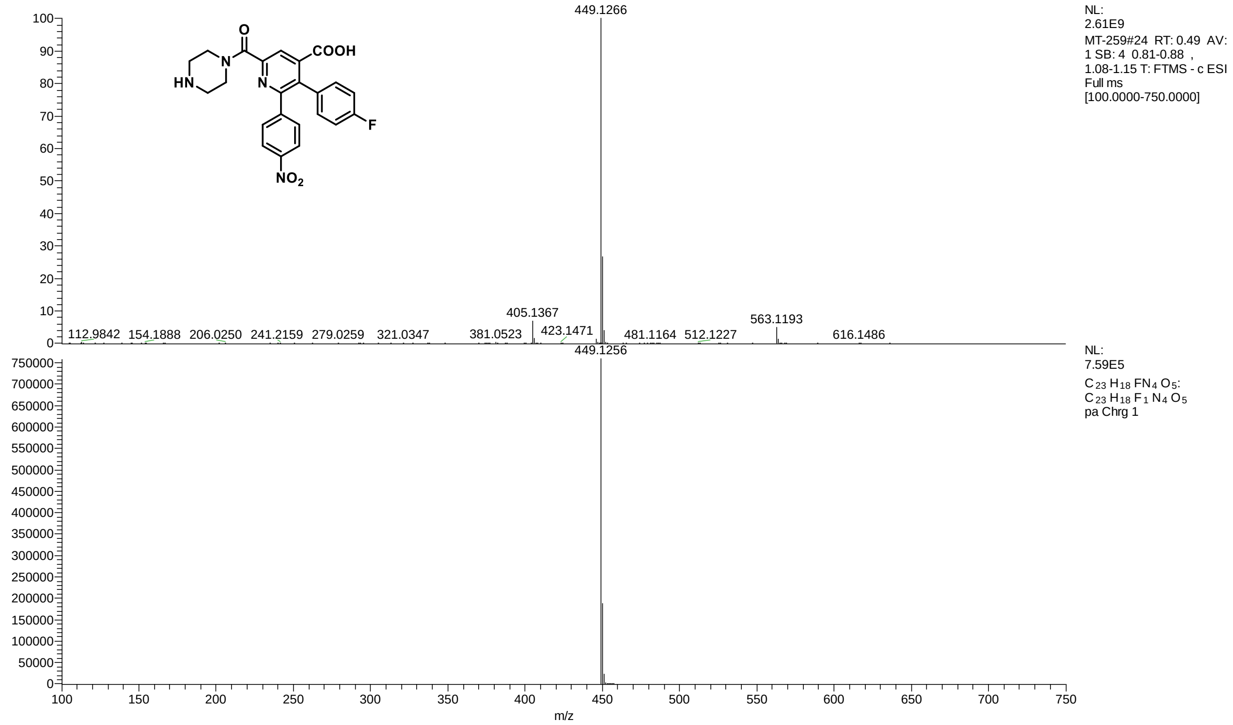

2-(4-nitrophenyl)-6-(piperazine-1-carbonyl)-3-(*p*-tolyl)isonicotinic acid (6e)

ESI+

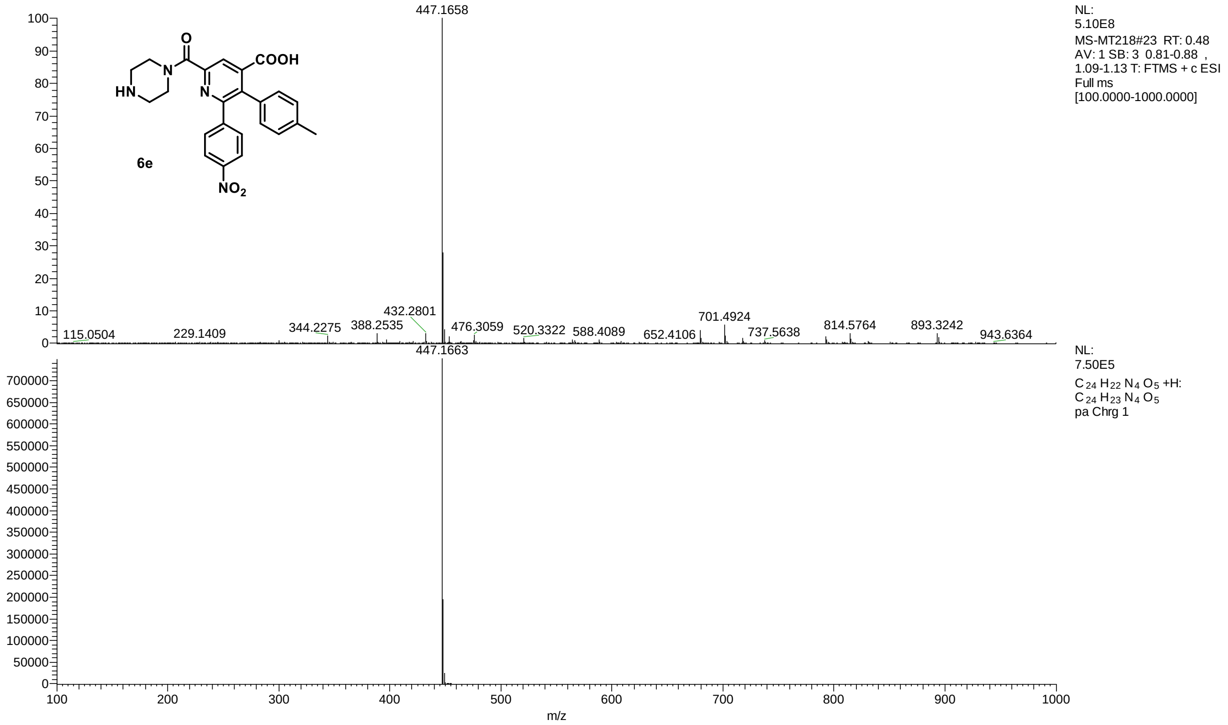

ESI-

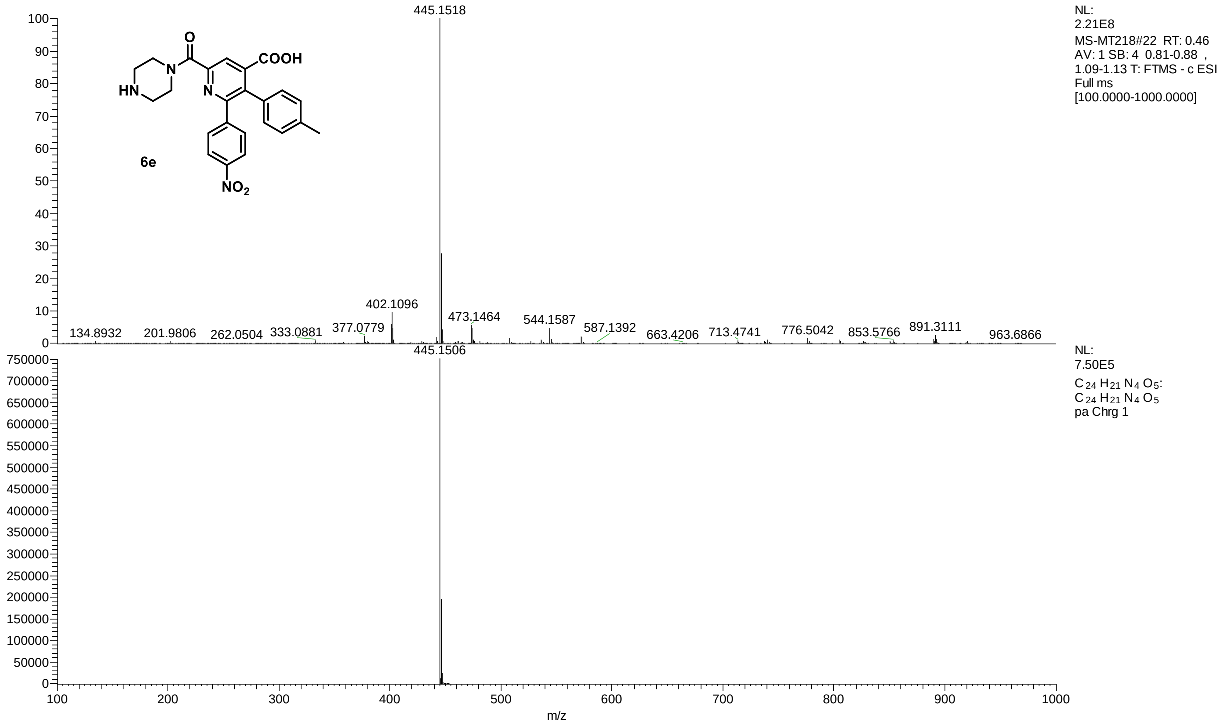

3-(3-methoxyphenyl)-2-(4-nitrophenyl)-6-(piperazine-1-carbonyl)isonicotinic acid (6f)

ESI+

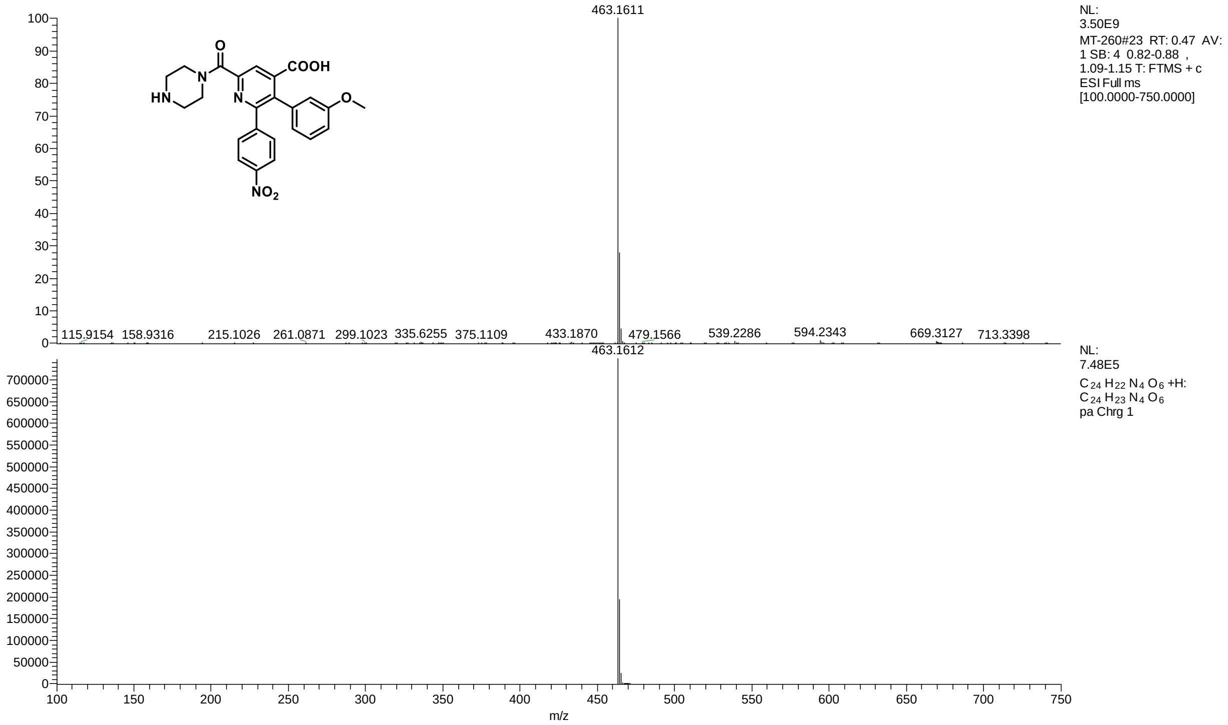

ESI-

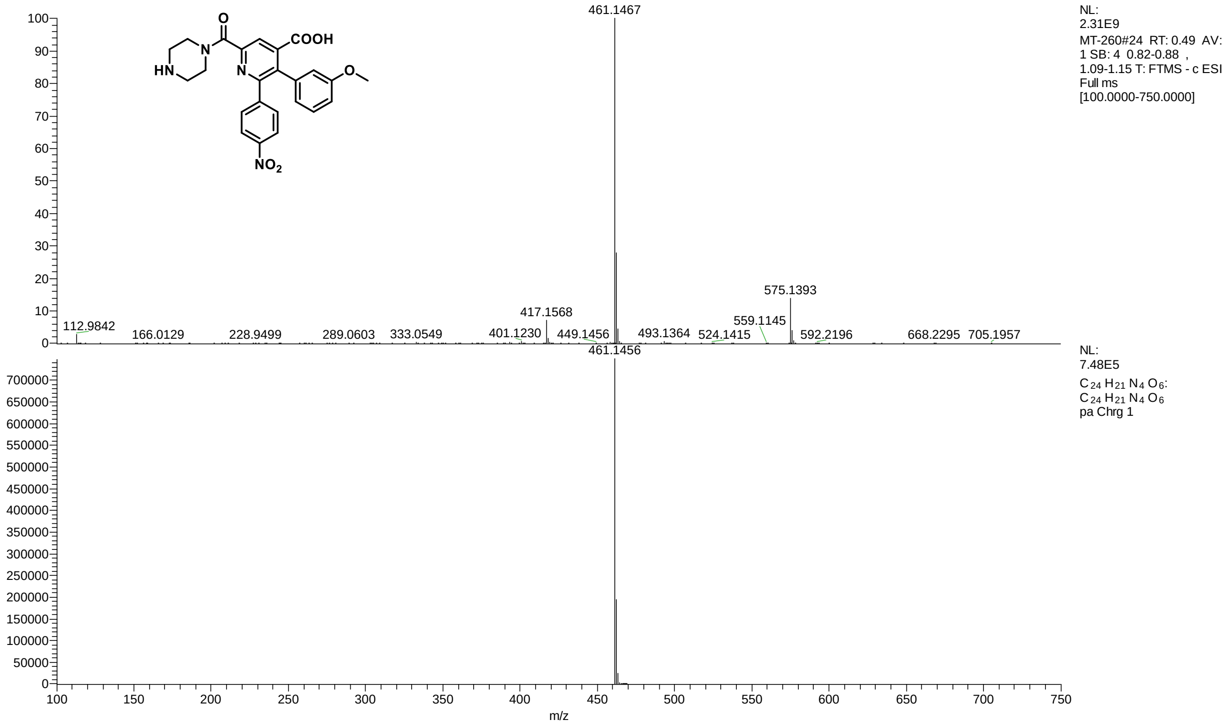

3-(4-amino-3,5-dichlorophenyl)-2-(4-nitrophenyl)-6-(piperazine-1-carbonyl)isonicotinic acid (6g)

ESI+

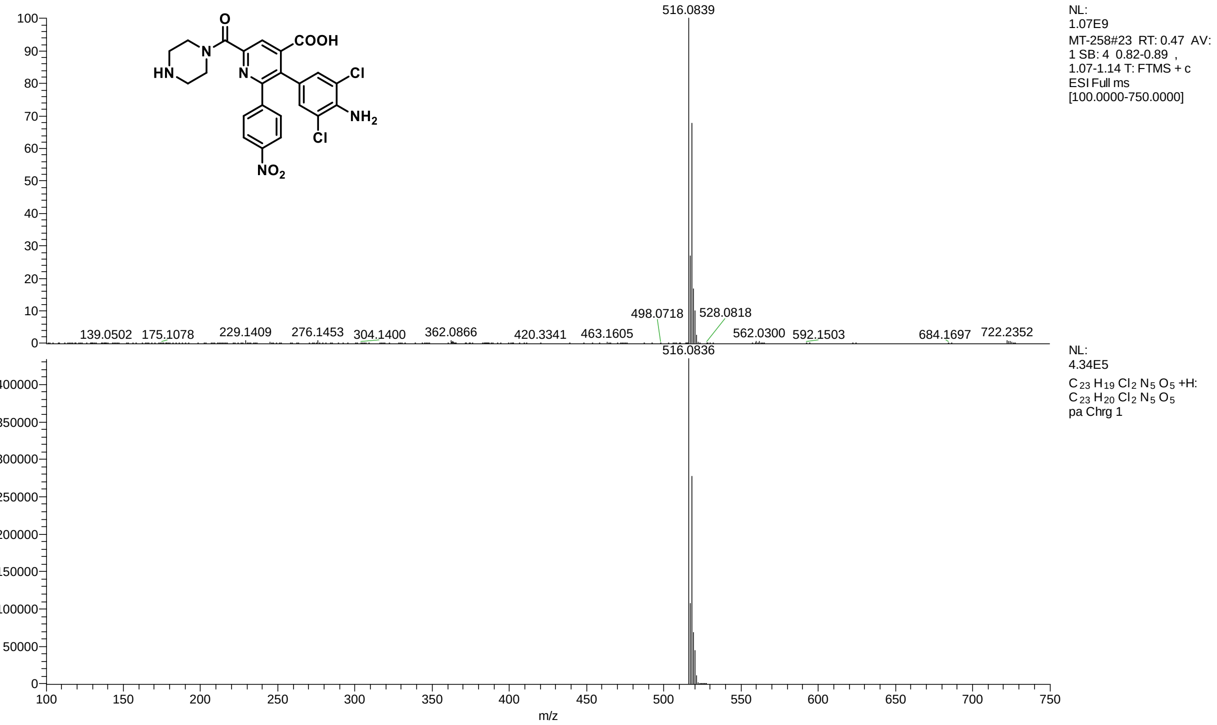

Zoom

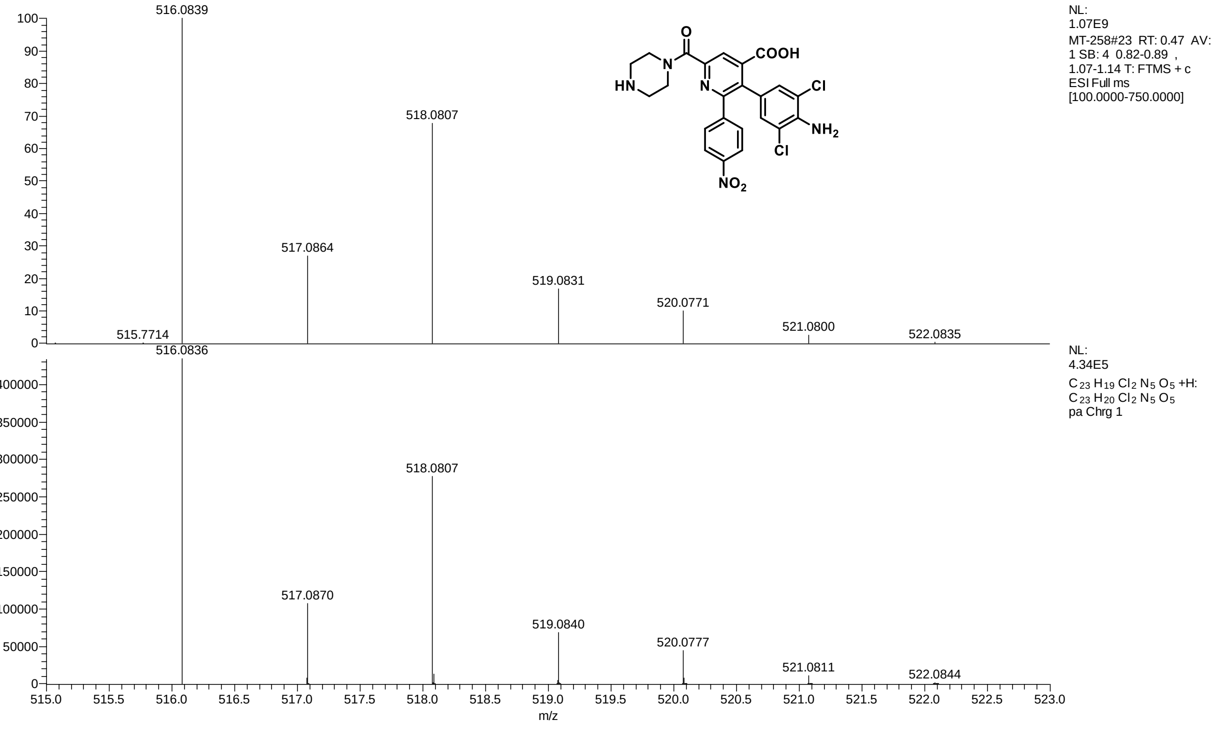

ESI-

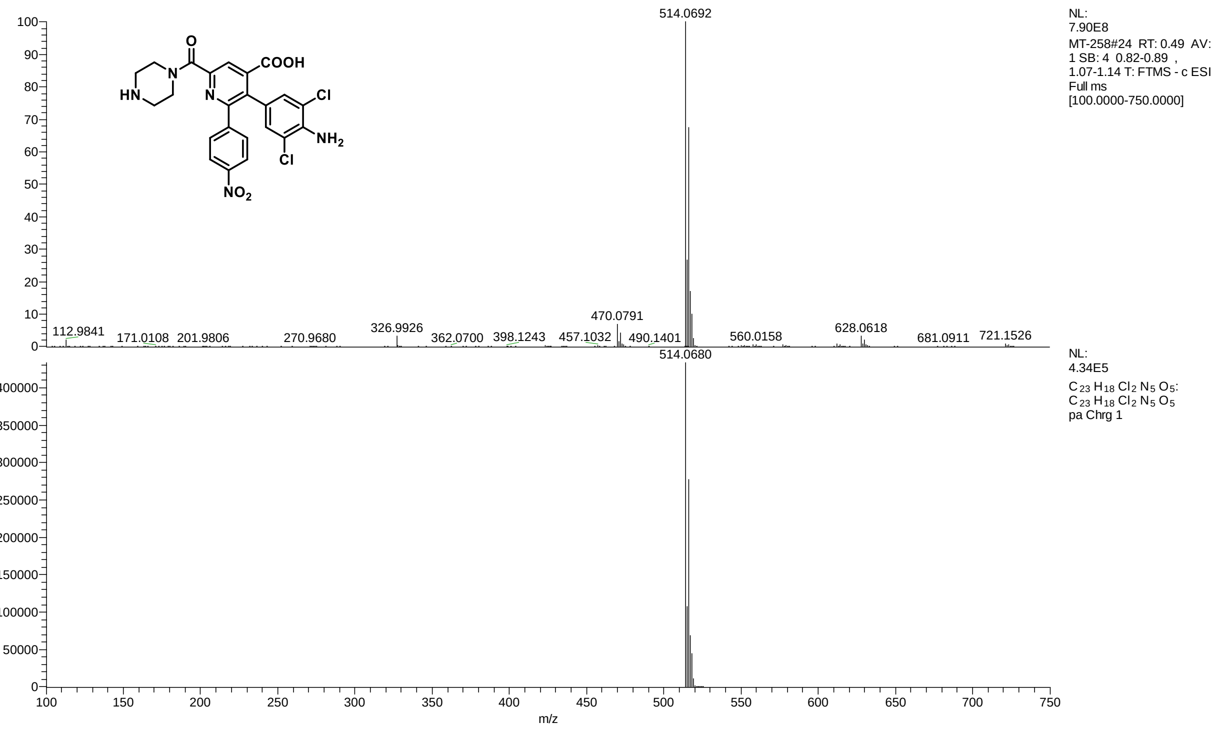

514.0692
NL:
7.90E8
MT-258#24 RT: 0.49 AV:
1 SB: 4 0.82-0.89 ,
1.07-1.14 T: FTMS - c ESI
Full ms
[100.0000-750.0000]
COOH
HN
N
O
Cl
NH2
NO2
112.9841
171.0108
201.9806
270.9680
326.9926
362.0700
398.1243
457.1032
470.0791
490.1401
560.0158
628.0618
681.0911
721.1526
514.0680
NL:
4.34E5
C 23 H18 Cl2 N5 O5:
C 23 H18 Cl2 N5 O5
pa Chrg 1
m/z

Zoom

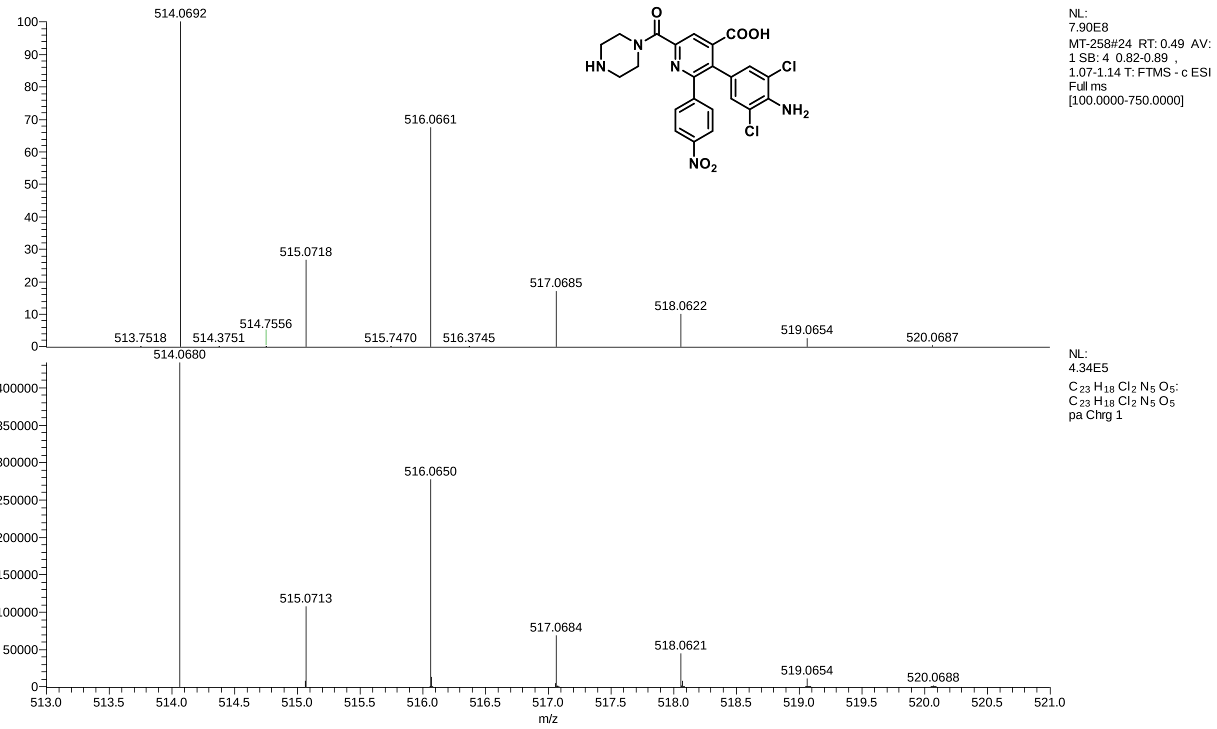

514.0692
NL:
7.90E8
MT-258#24 RT: 0.49 AV:
1 SB: 4 0.82-0.89 ,
1.07-1.14 T: FTMS - c ESI
Full ms
[100.0000-750.0000]
516.0661
515.0718
517.0685
518.0622
514.7556
513.7518
514.3751
515.7470
516.3745
519.0654
520.0687
514.0680
NL:
4.34E5
C 23 H18 Cl2 N5 O5:
C 23 H18 Cl2 N5 O5
pa Chrg 1
516.0650
515.0713
517.0684
518.0621
519.0654
520.0688
m/z

ethyl 3-(3-methoxyphenyl)-2-(4-nitrophenyl)-6-(piperazine-1-carbonyl)isonicotinate (6h)

ESI+

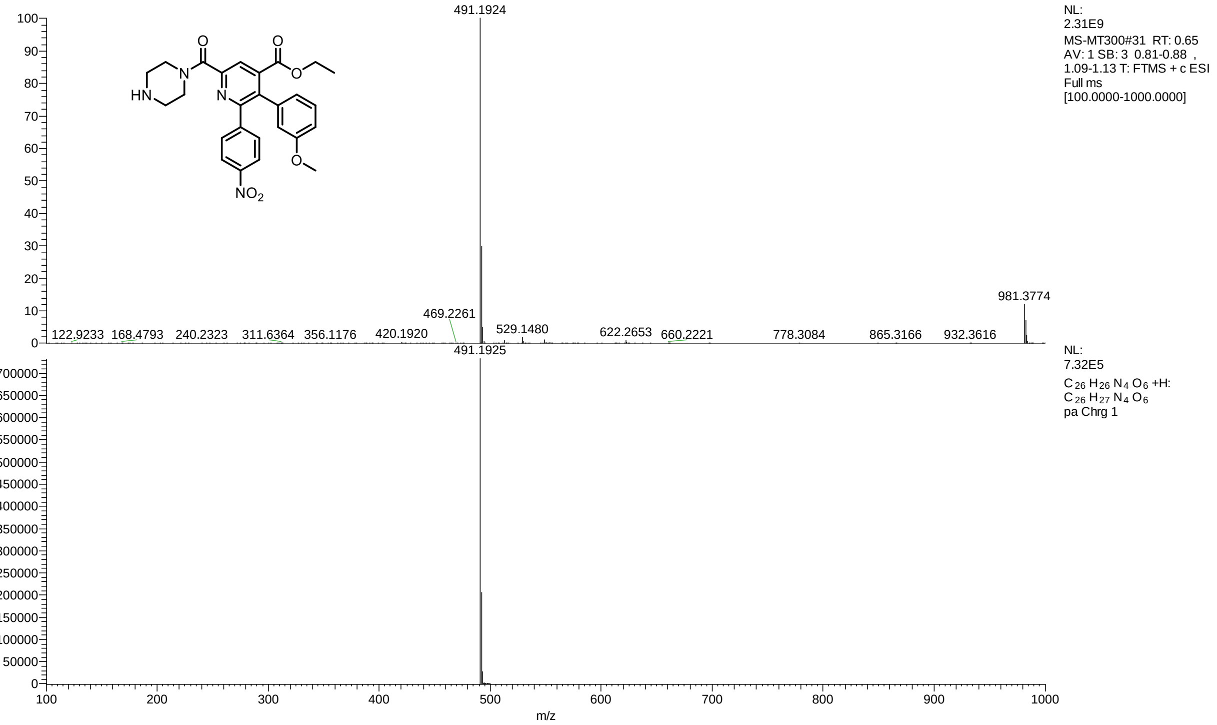

(3-(3-methoxyphenyl)-2-(4-nitrophenyl)-6-(piperazine-1-carbonyl)pyridin-4-yl)(piperidin-1-yl)methanone (6i)

ESI+

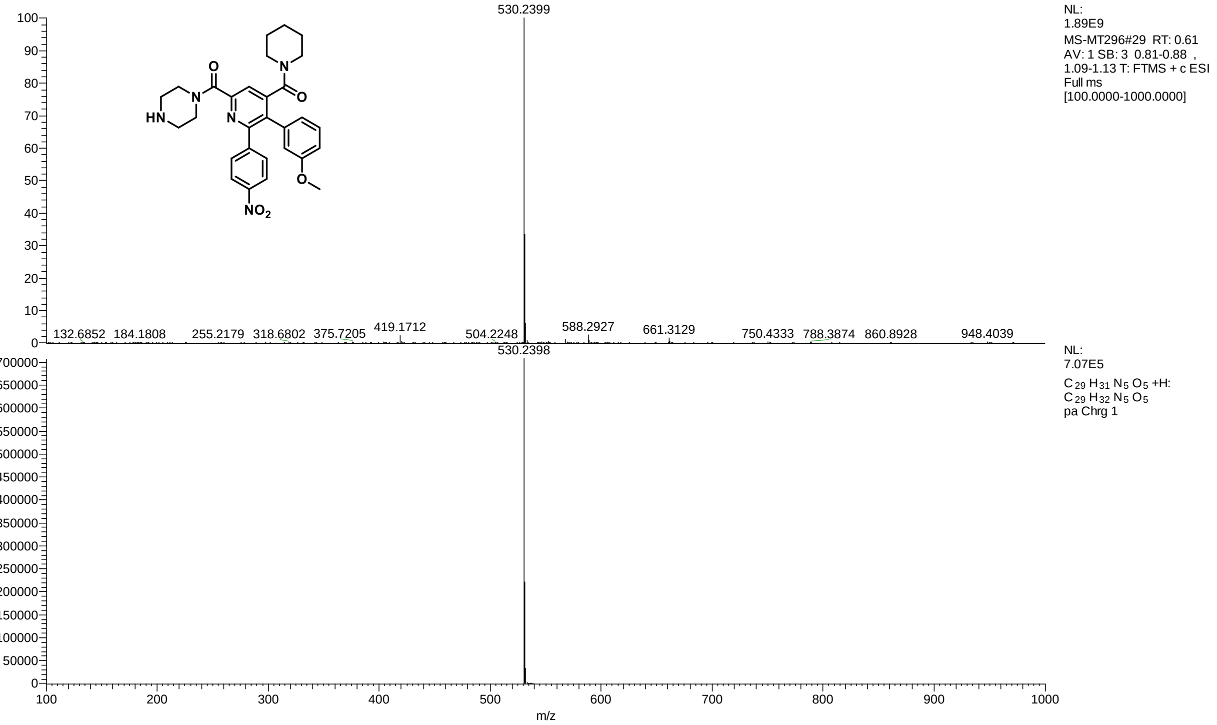

2-(4-nitrophenyl)-6-(piperazine-1-carbonyl)-3-(thiophen-3-yl)isonicotinic acid (6j)

ESI+

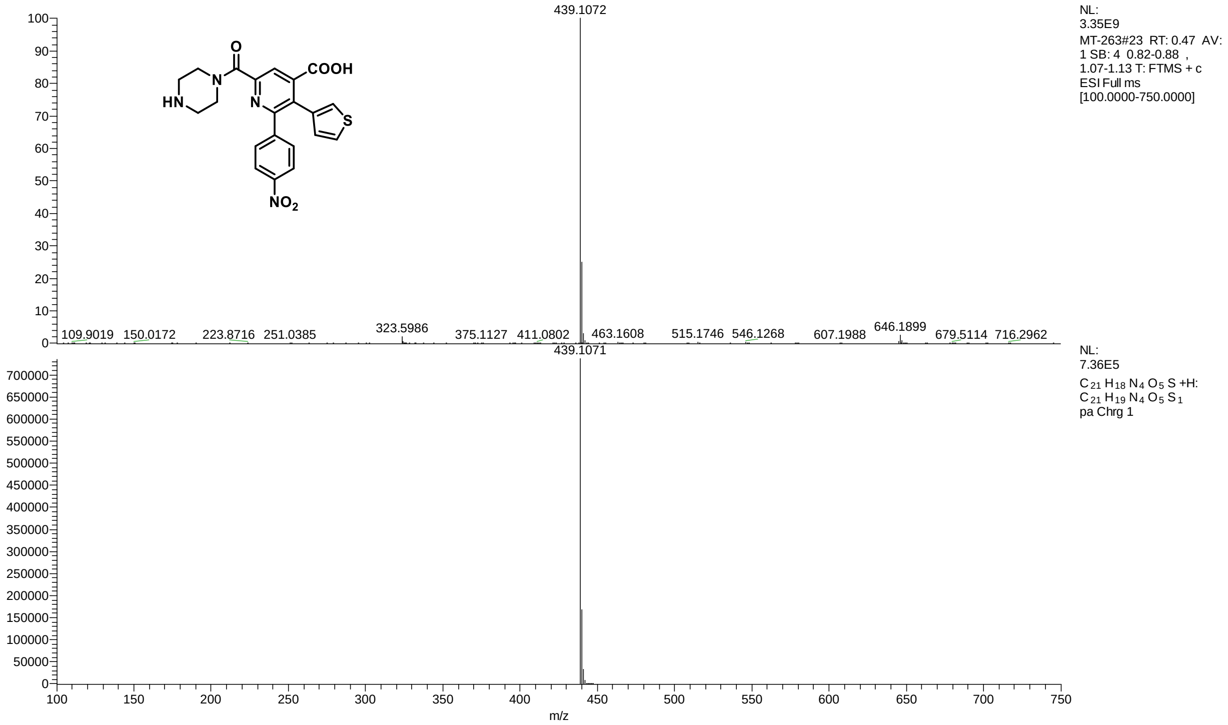

ESI-

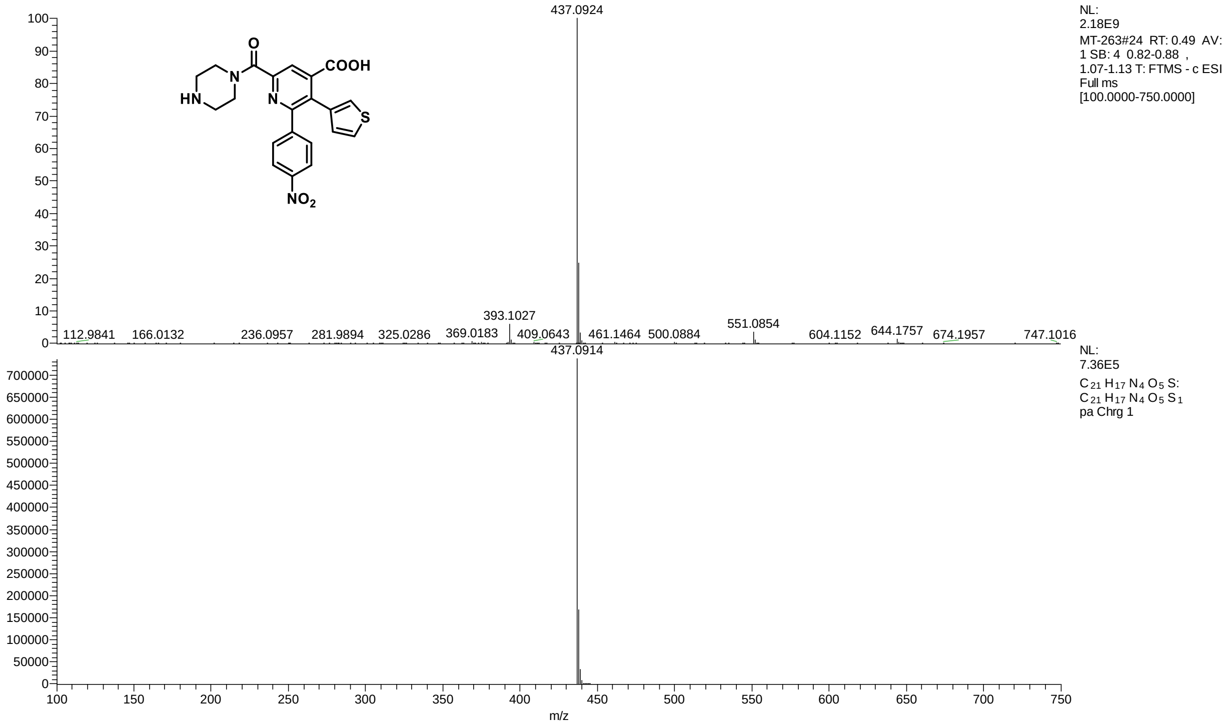

3-(3-methoxyphenyl)-2-(4-nitrophenyl)-6-(piperazine-1-carbonyl)-N-propylisonicotinamide (6k)

ESI+

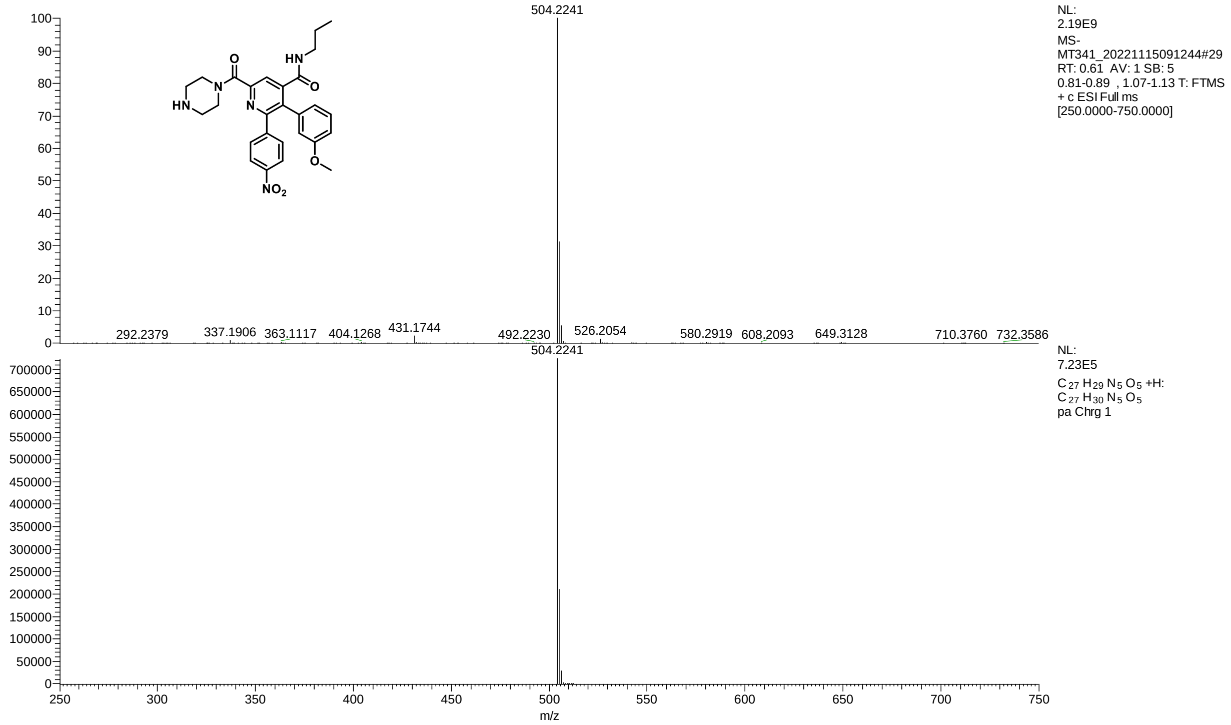

ESI-

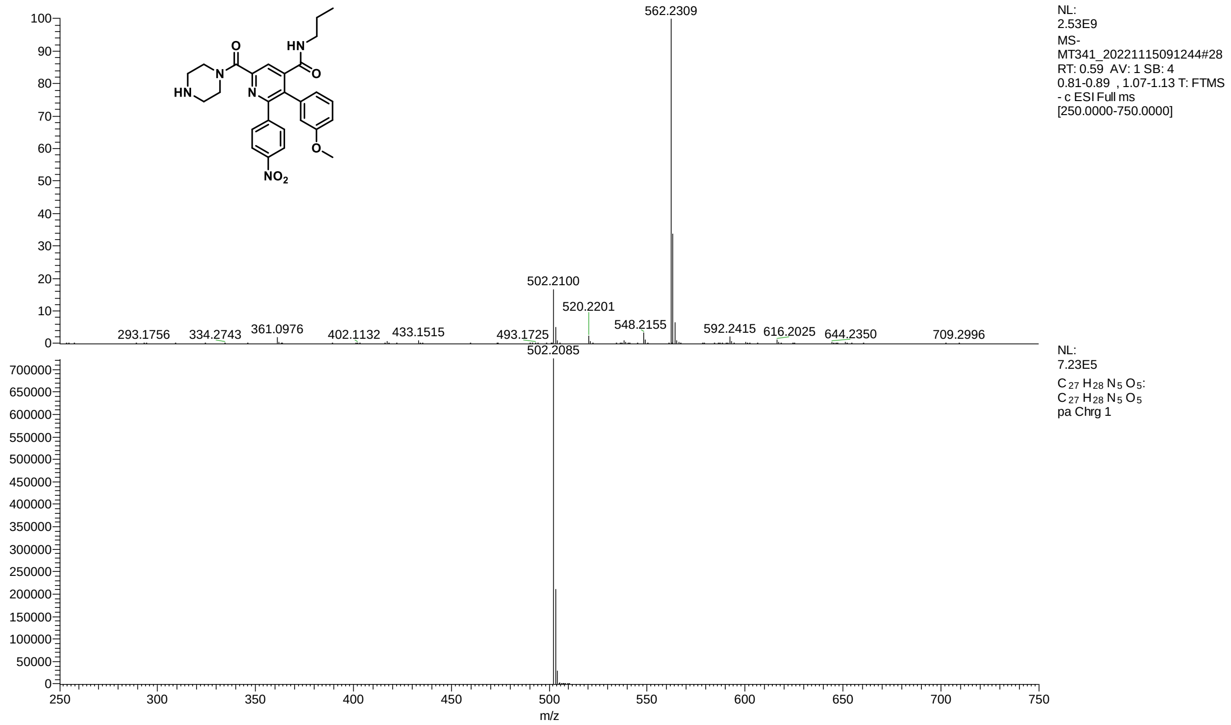

3-(4-methoxyphenyl)-2-(4-nitrophenyl)-6-(propylcarbamoyl)isonicotinic acid (6l)

ESI+

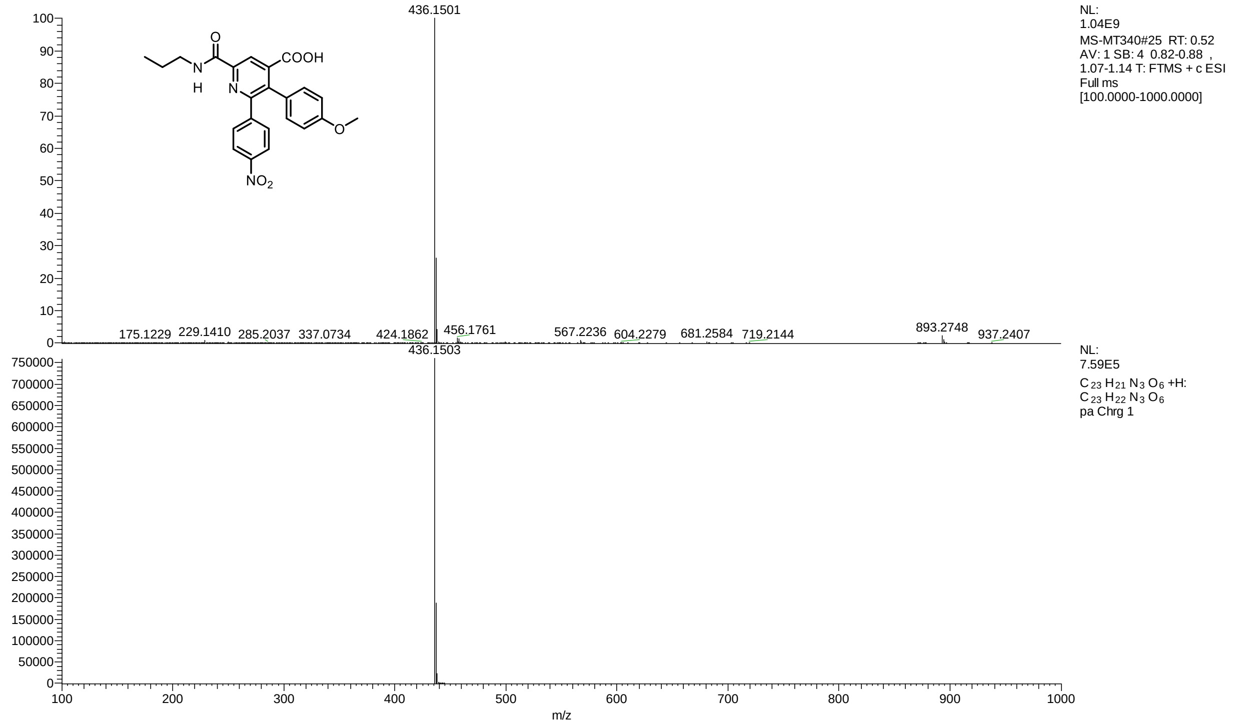

ESI-

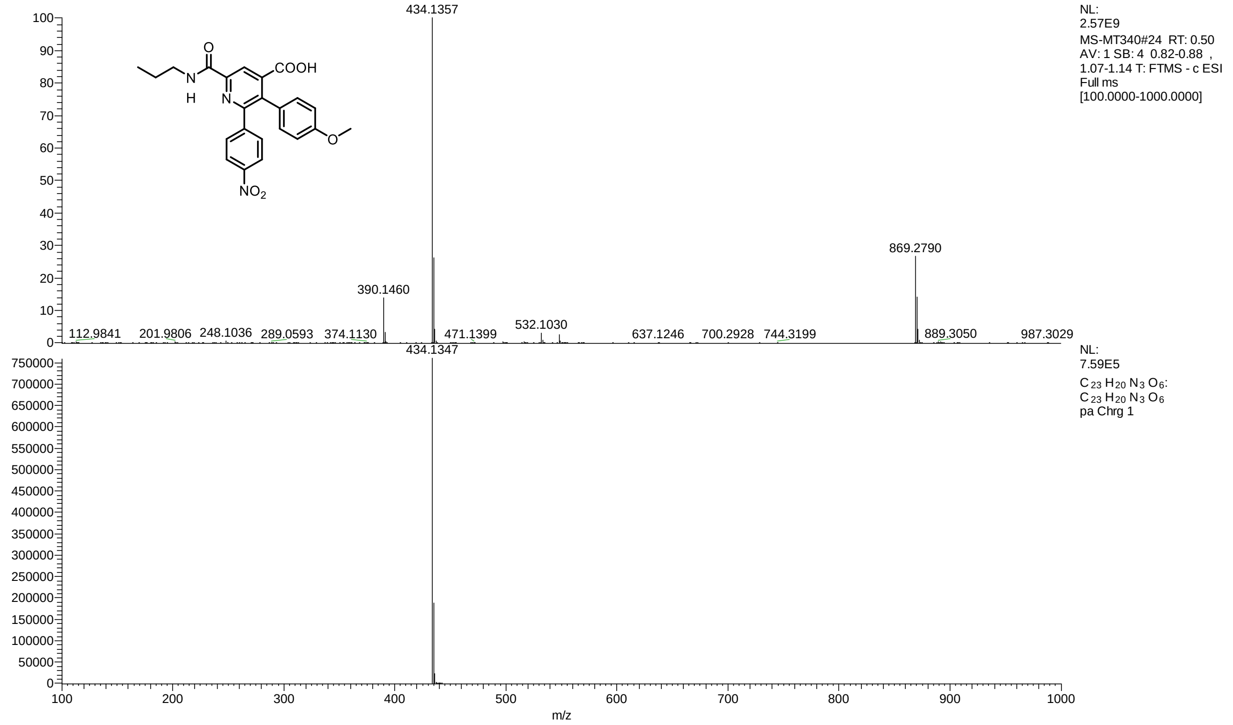

3-(6-(4-nitrophenyl)-3-oxo-5-(*p*-tolyl)-3,4-dihydropyrazin-2-yl)propanoic acid (7a)

ESI+

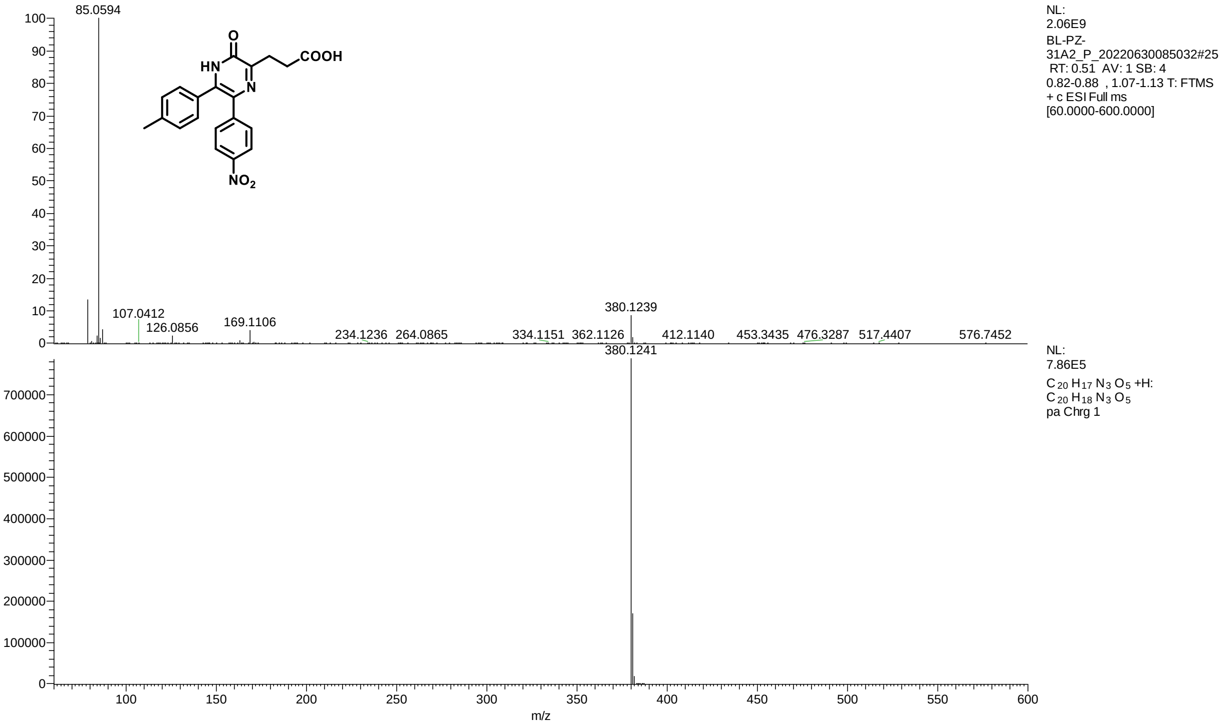

ESI-

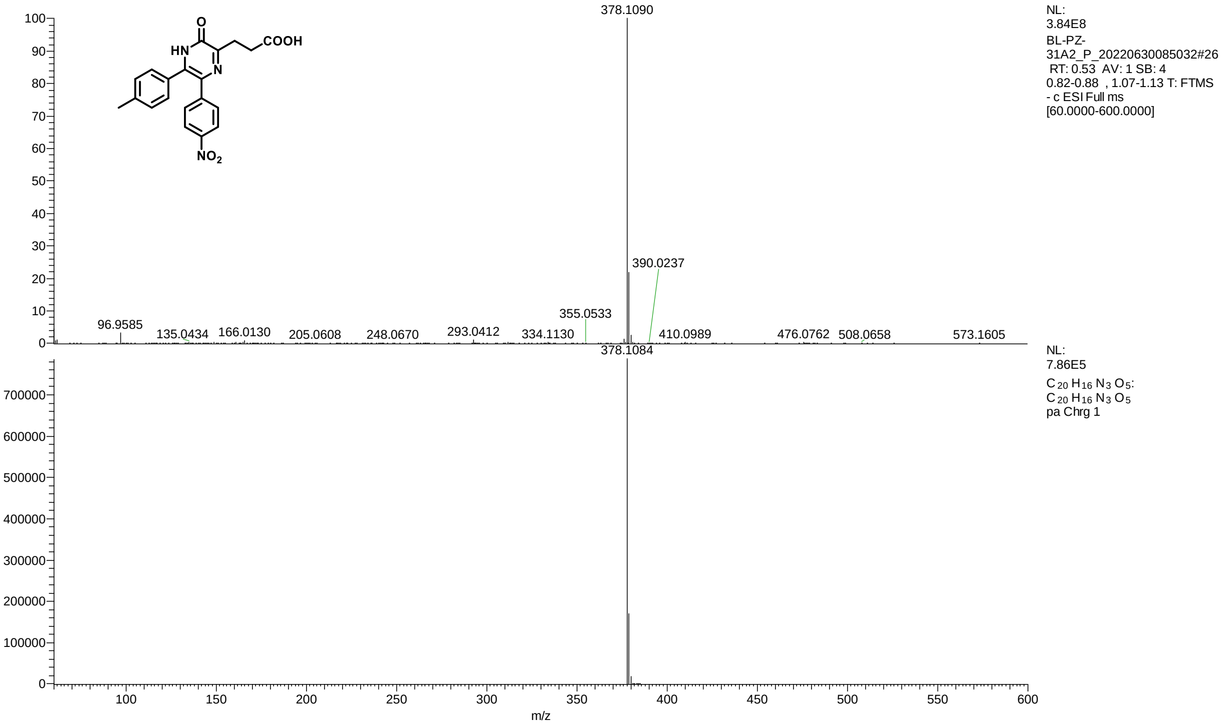

Methyl 3-(5-(4-methoxyphenyl)-6-(4-nitrophenyl)-3-oxo-3,4-dihydropyrazin-2-yl)propanoate (7b)

ESI+

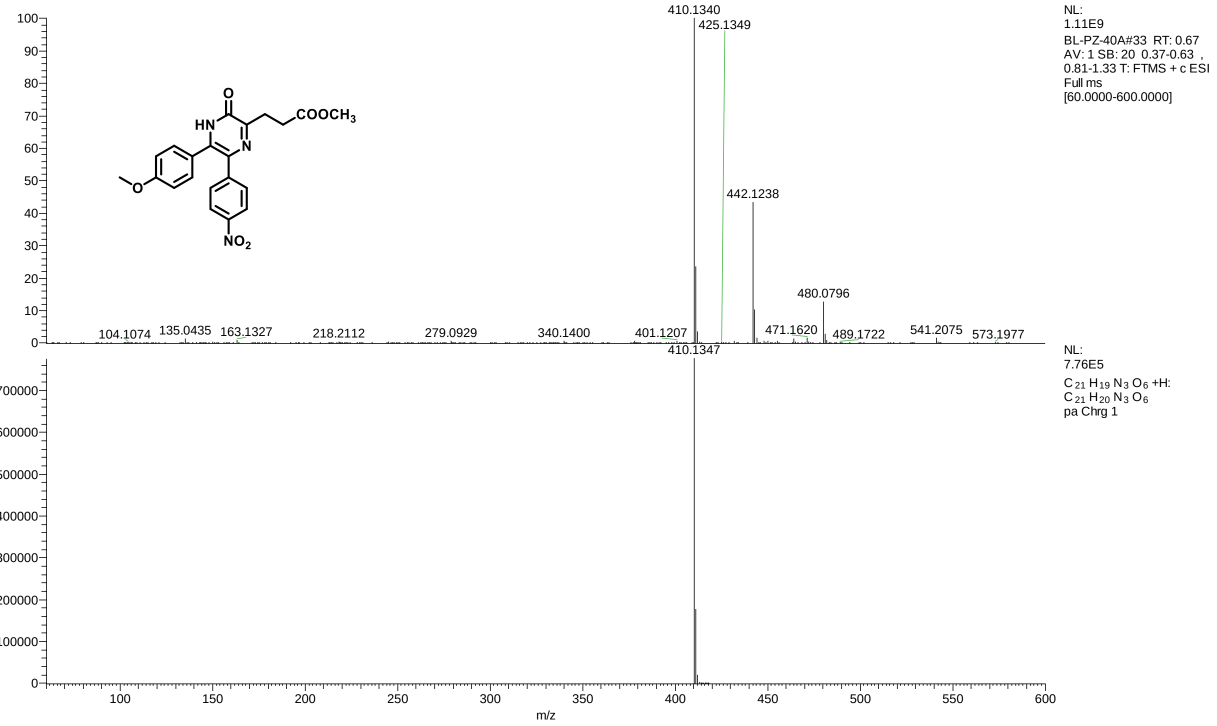

ESI-

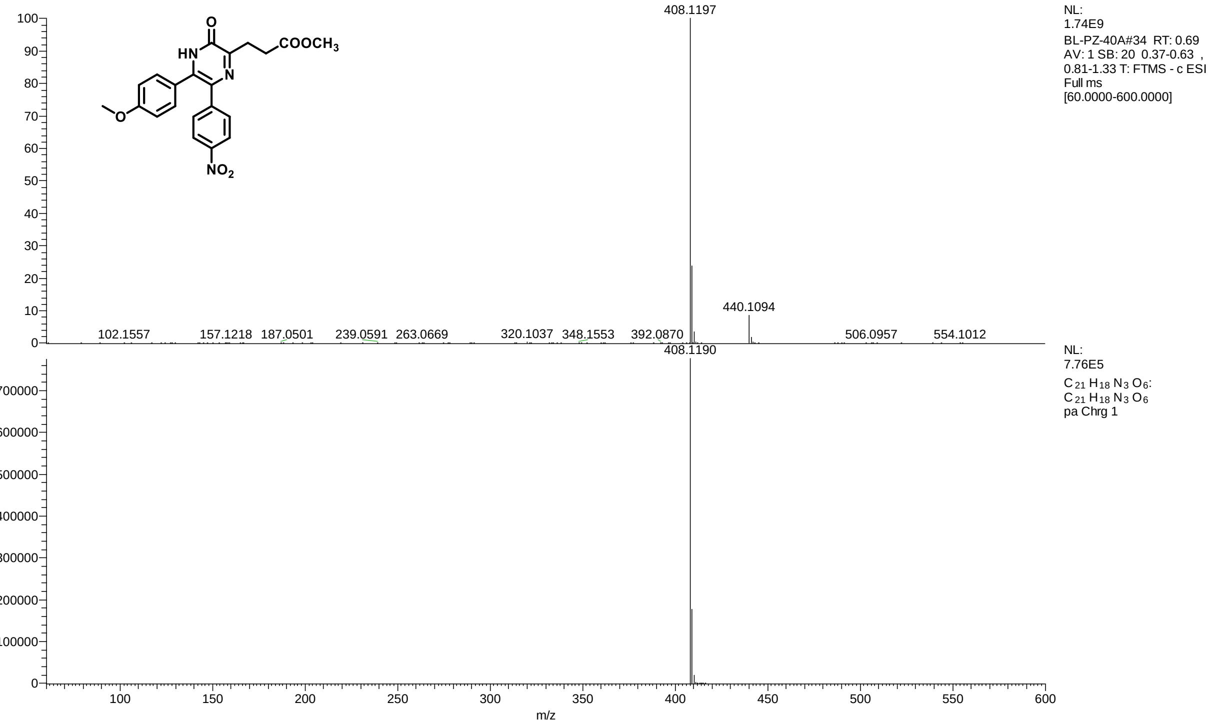

5-(4-nitrophenyl)-6-(*p*-tolyl)pyrazin-2(1*H*)-one (7c)

ESI+

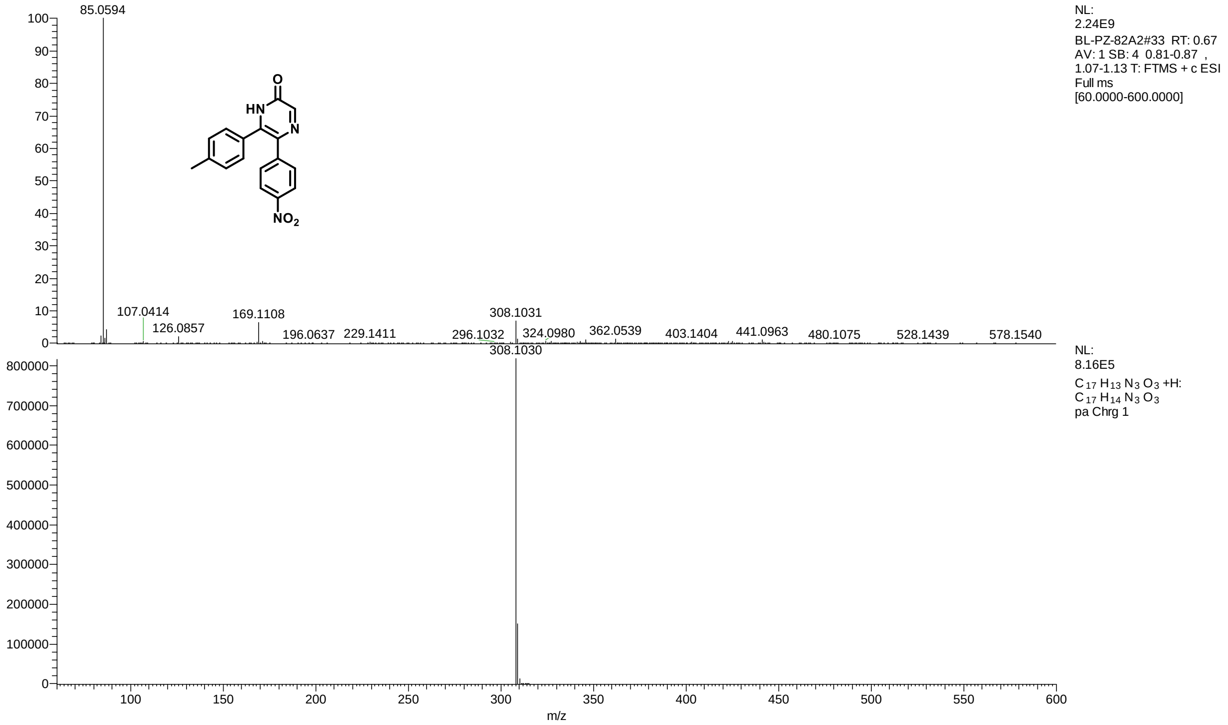

ESI-

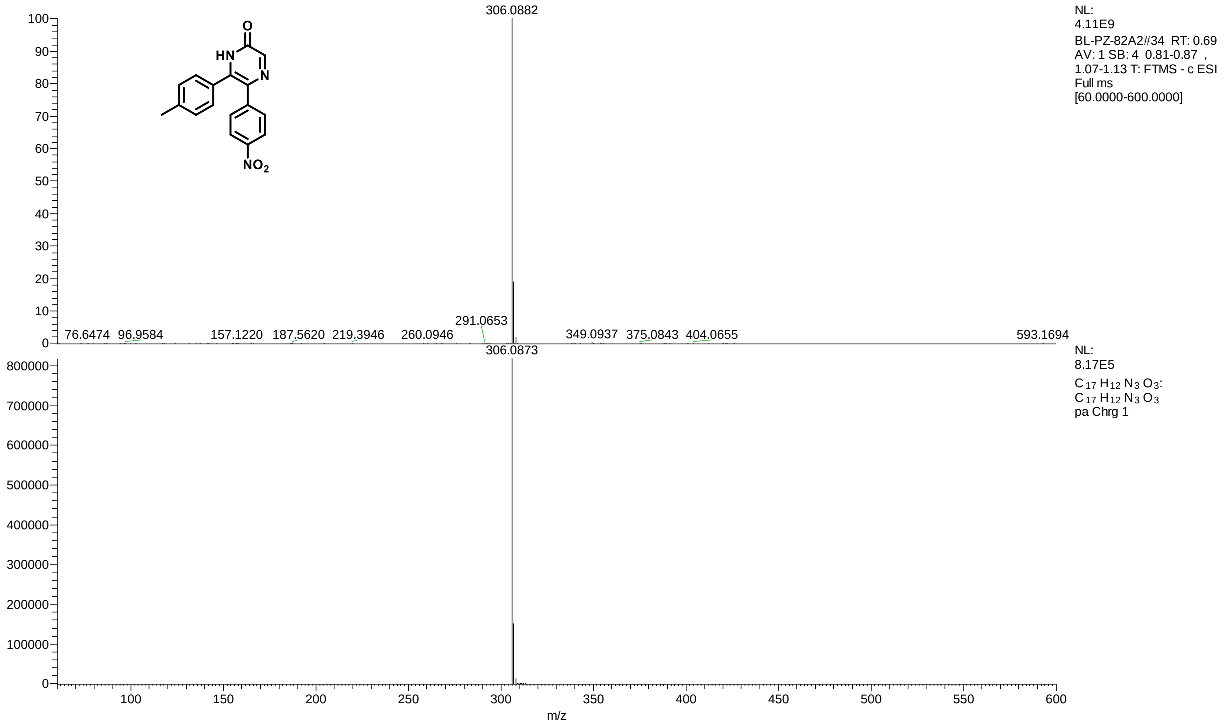

3-methyl-5-(4-nitrophenyl)-6-(*p*-tolyl)pyrazin-2(1*H*)-one (7d)

ESI+

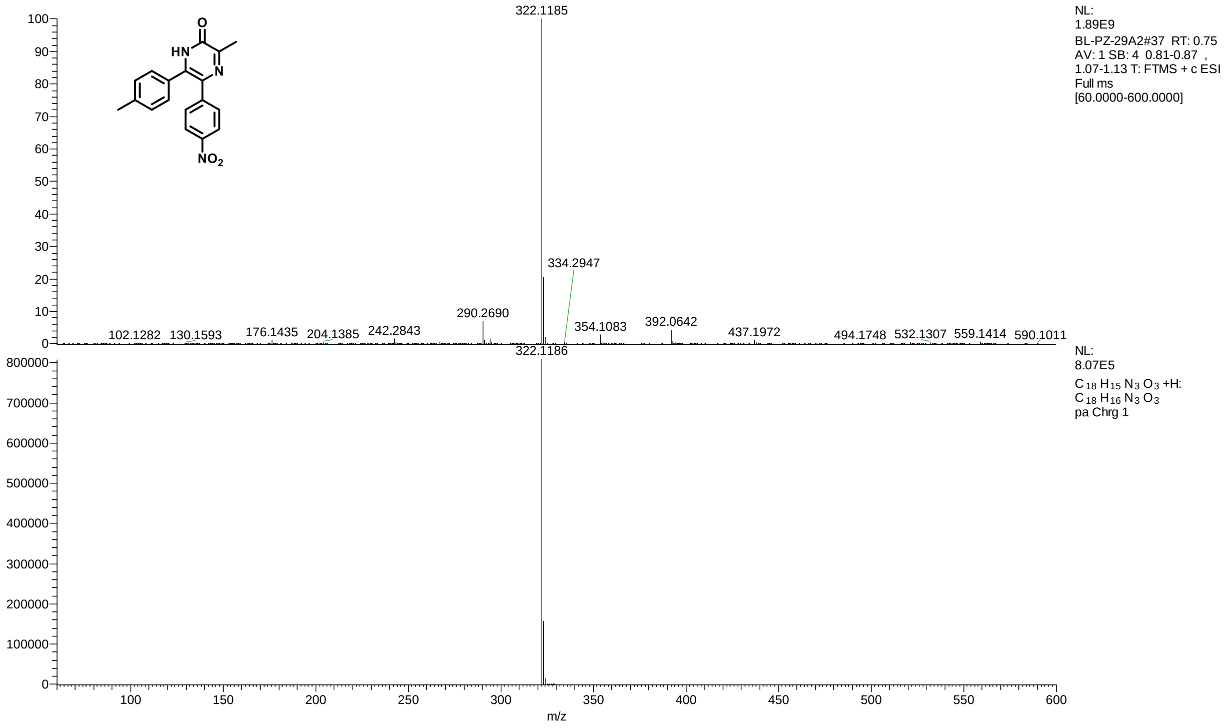

322.1185
334.2947
290.2690
102.1282 130.1593 176.1435 204.1385 242.2843 354.1083 392.0642 437.1972 494.1748 532.1307 559.1414 590.1011
NL: 1.89E9 BL-PZ-29A2#37 RT: 0.75 AV: 1 SB: 4 0.81-0.87 , 1.07-1.13 T: FTMS + c ESI Full ms [60.0000-600.0000]
322.1186
NL: 8.07E5
C 18 H 15 N 3 O 3 +H: C 18 H 16 N 3 O 3 pa Chrg 1
m/z

ESI-

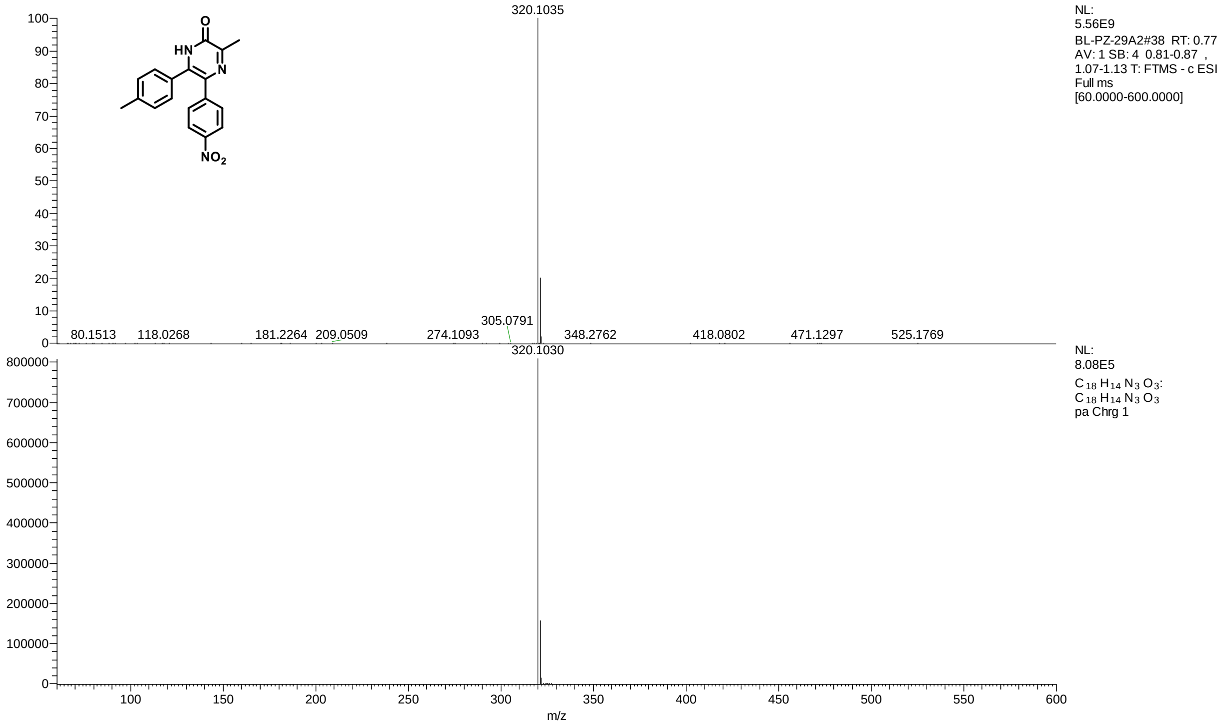

320.1035
305.0791
80.1513 118.0268 181.2264 209.0509 274.1093 348.2762 418.0802 471.1297 525.1769
NL: 5.56E9 BL-PZ-29A2#38 RT: 0.77 AV: 1 SB: 4 0.81-0.87 , 1.07-1.13 T: FTMS - c ESI Full ms [60.0000-600.0000]
320.1030
NL: 8.08E5
C 18 H 14 N 3 O 3: C 18 H 14 N 3 O 3 pa Chrg 1
m/z

3-isobutyl-5-(4-nitrophenyl)-6-(*p*-tolyl)pyrazin-2(1*H*)-one (7e)

ESI+

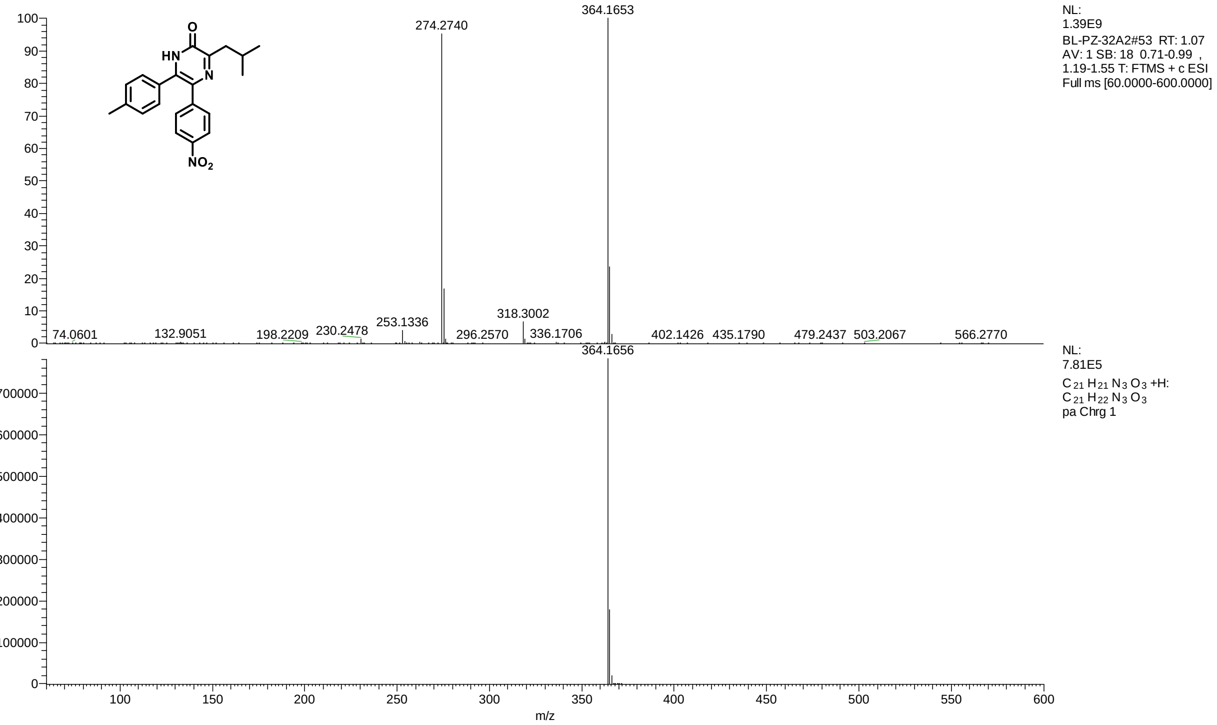

ESI-

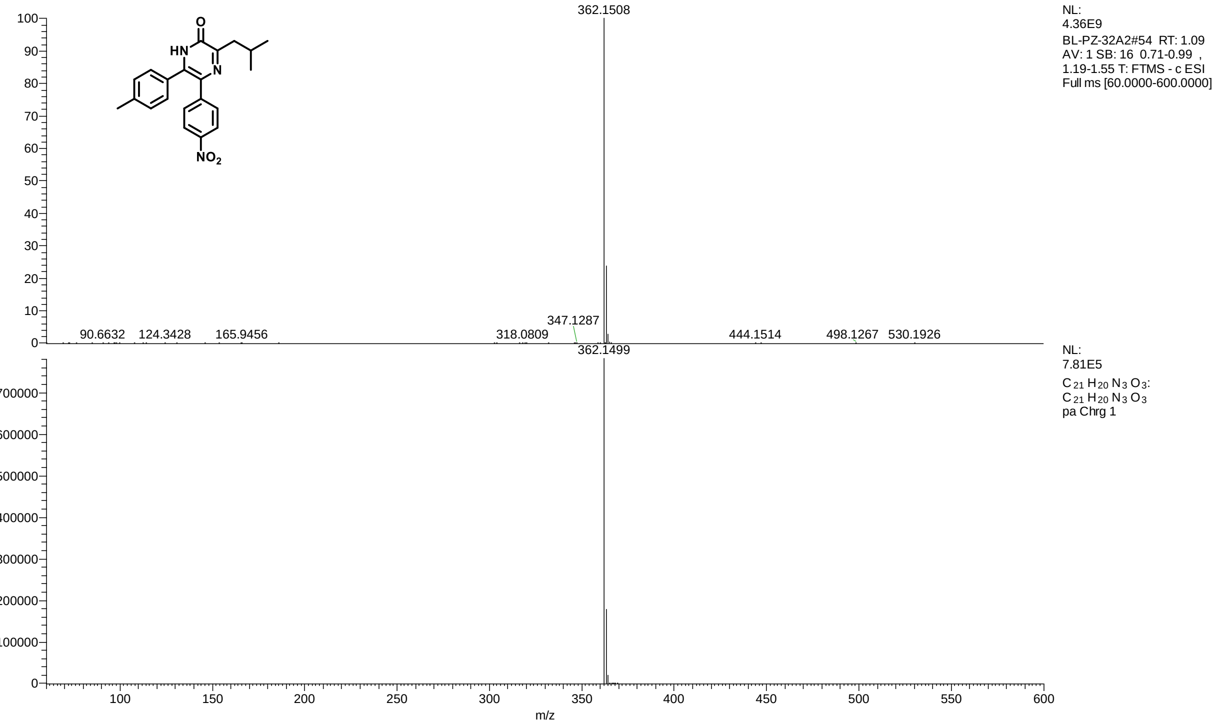

3-benzyl-5-(4-nitrophenyl)-6-(*p*-tolyl)pyrazin-2(1*H*)-one (7f)

ESI+

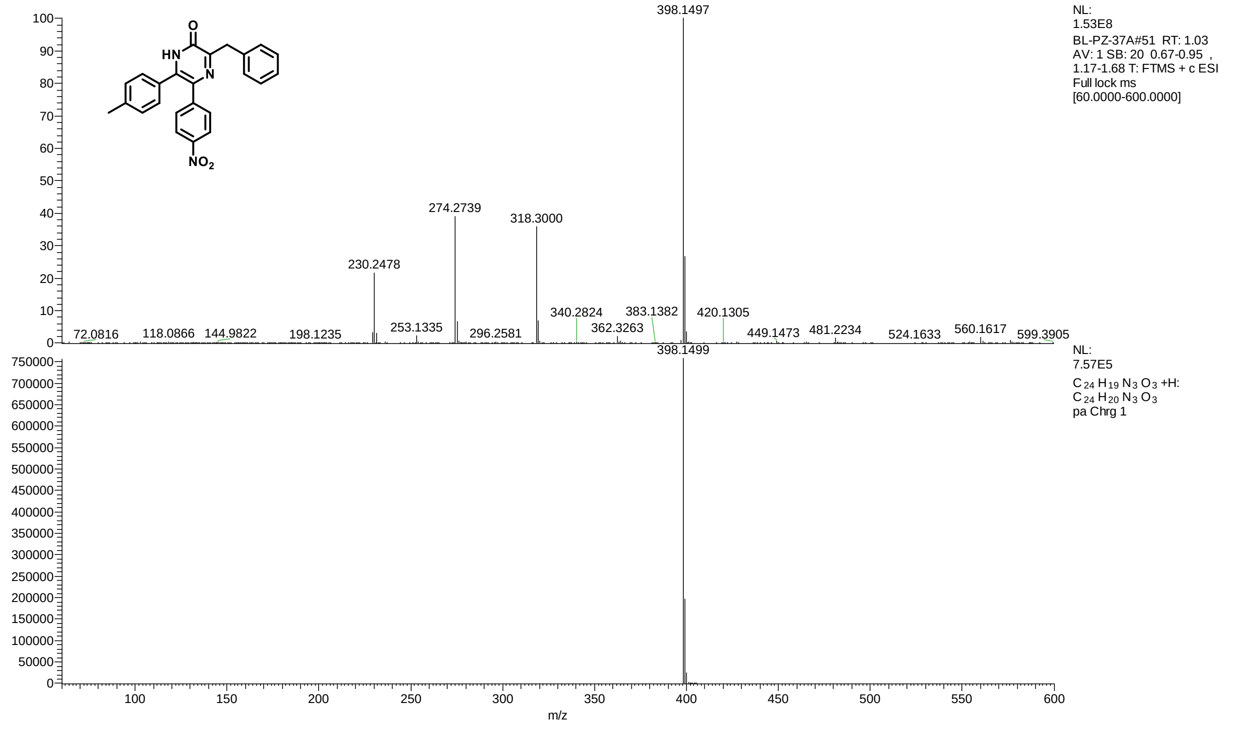

ESI-

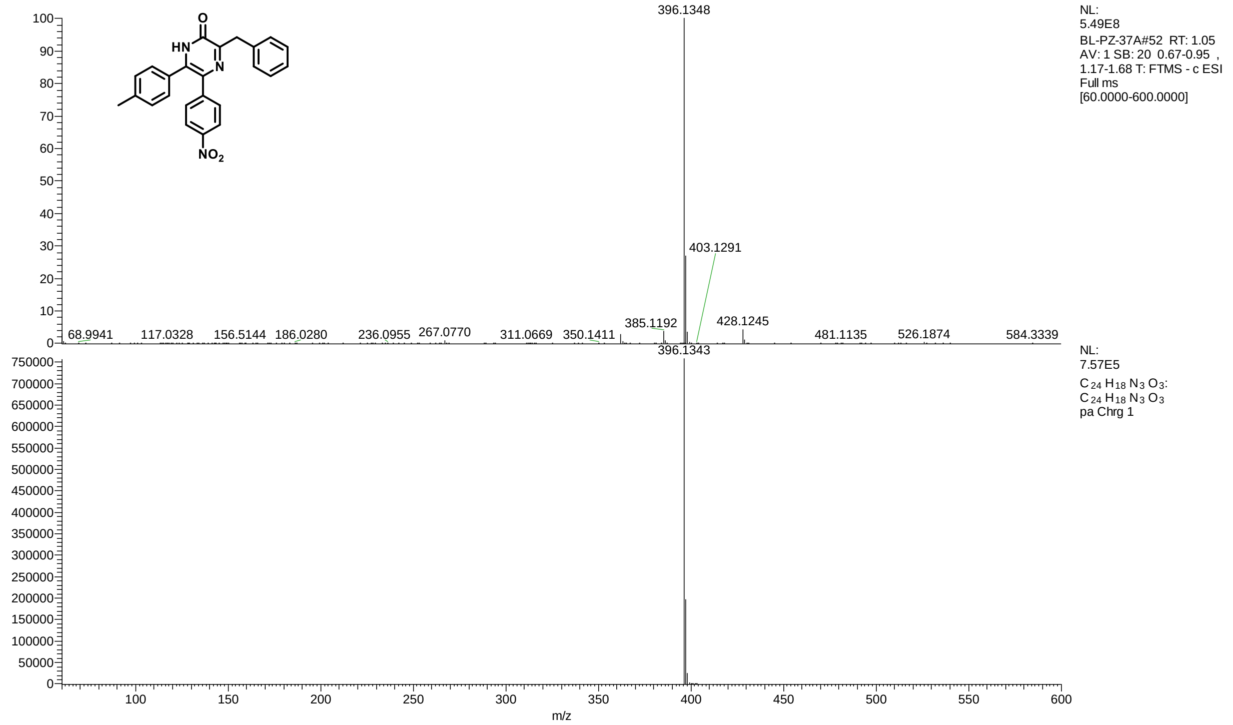

3-(4-hydroxybenzyl)-5-(4-nitrophenyl)-6-(*p*-tolyl)pyrazin-2(1*H*)-one (7g)

ESI+

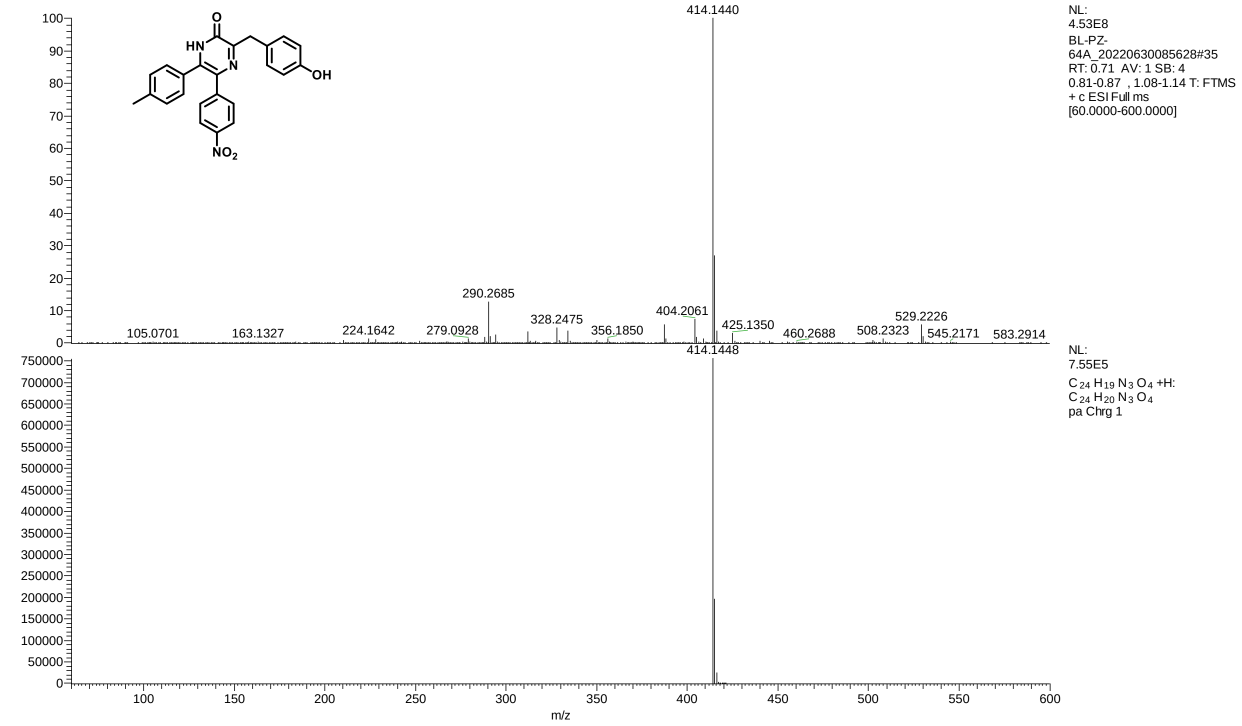

ESI-

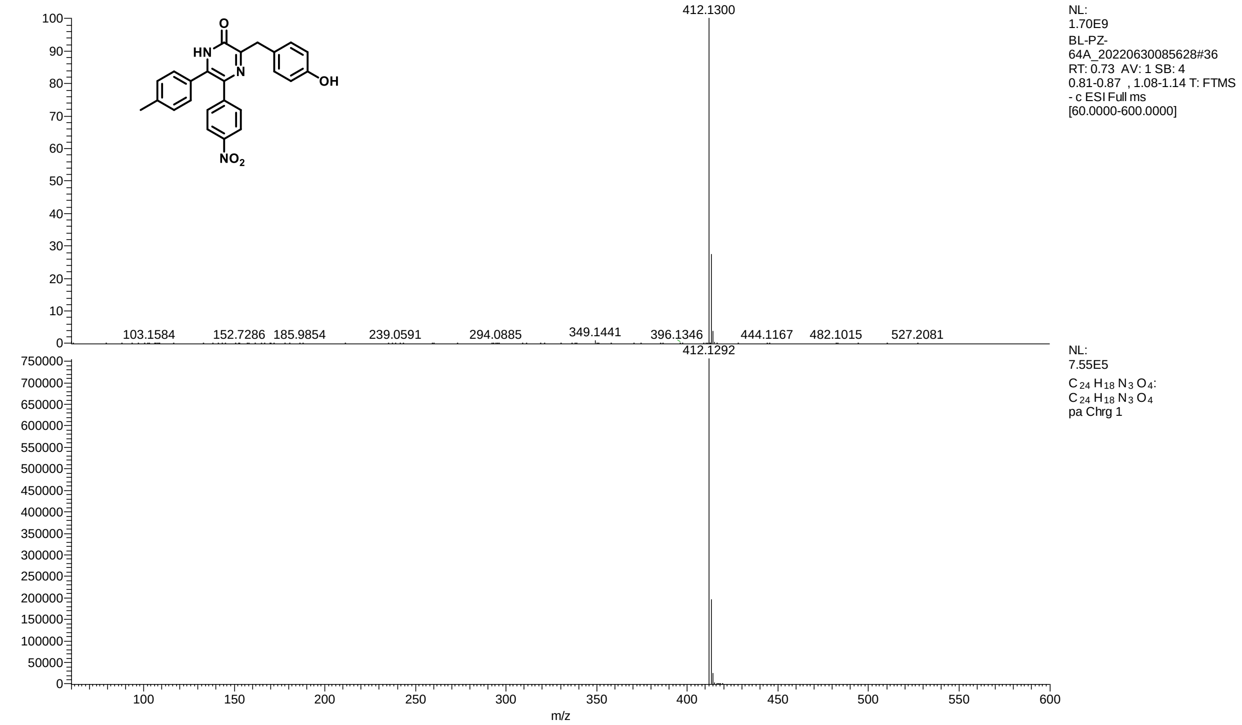

3-(2-(methylthio)ethyl)-5-(4-nitrophenyl)-6-(*p*-tolyl)pyrazin-2(1*H*)-one (7h)

ESI+

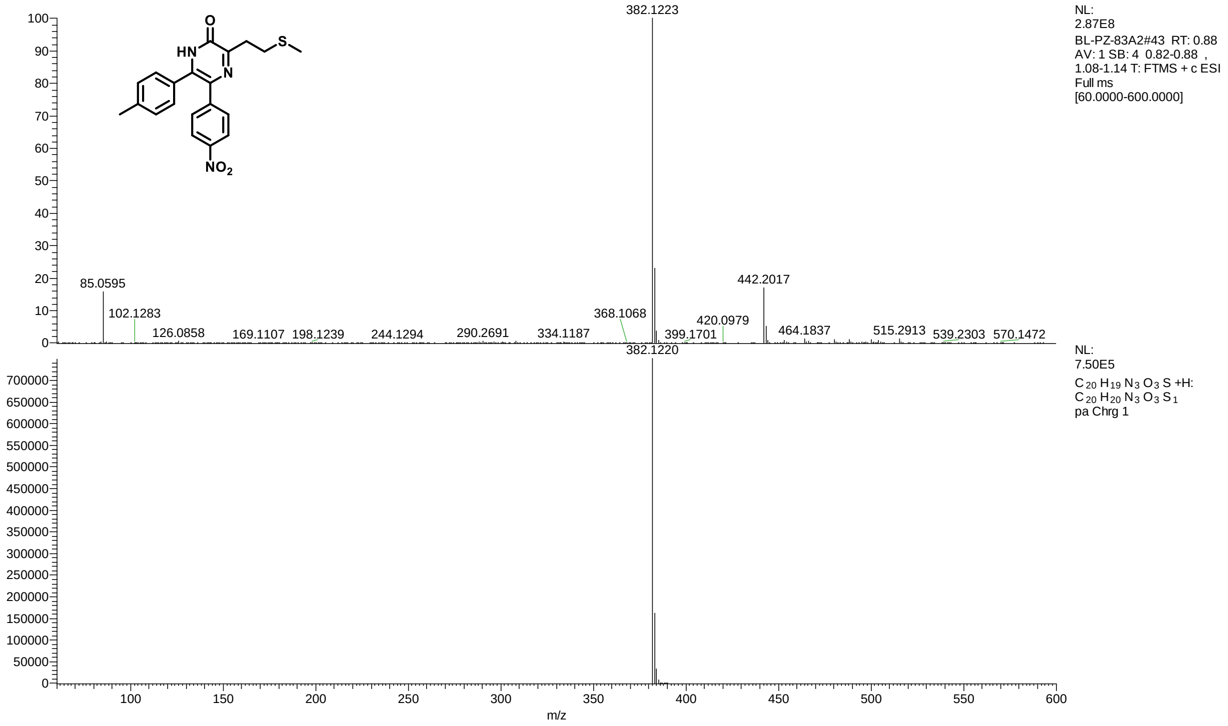

ESI-

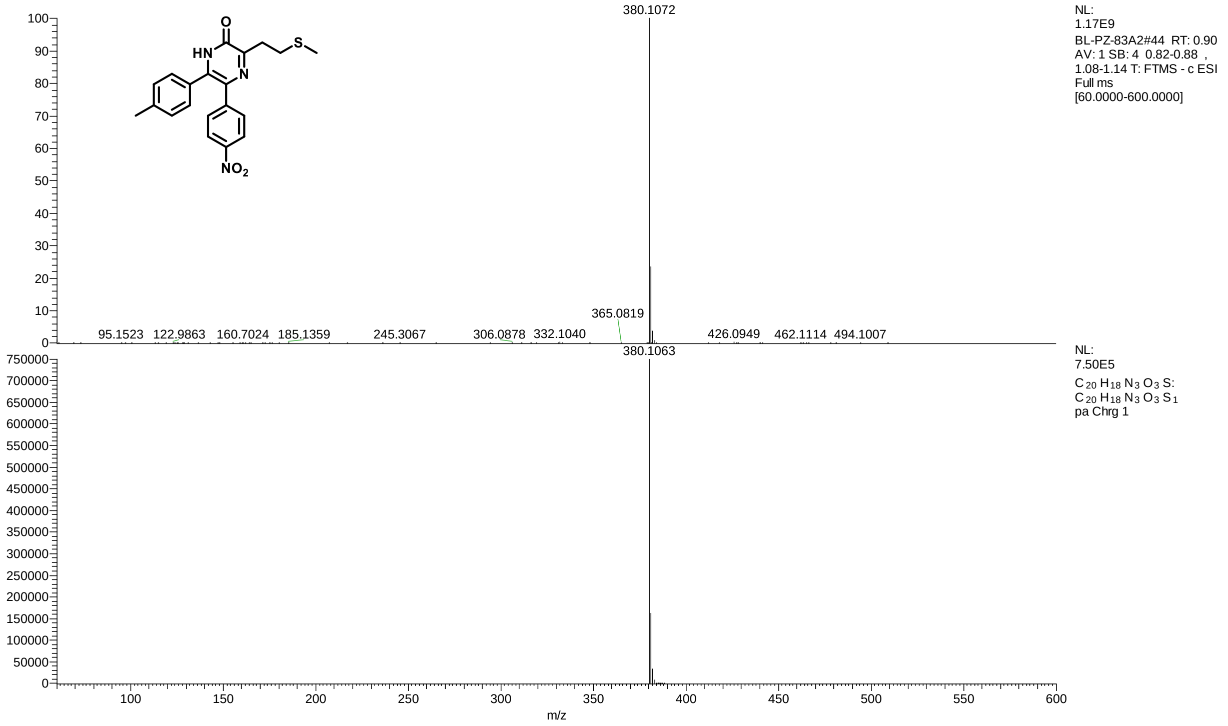

3-(6-(4-nitrophenyl)-3-oxo-5-(*p*-tolyl)-3,4-dihydropyrazin-2-yl)propanamide (7i)

ESI+

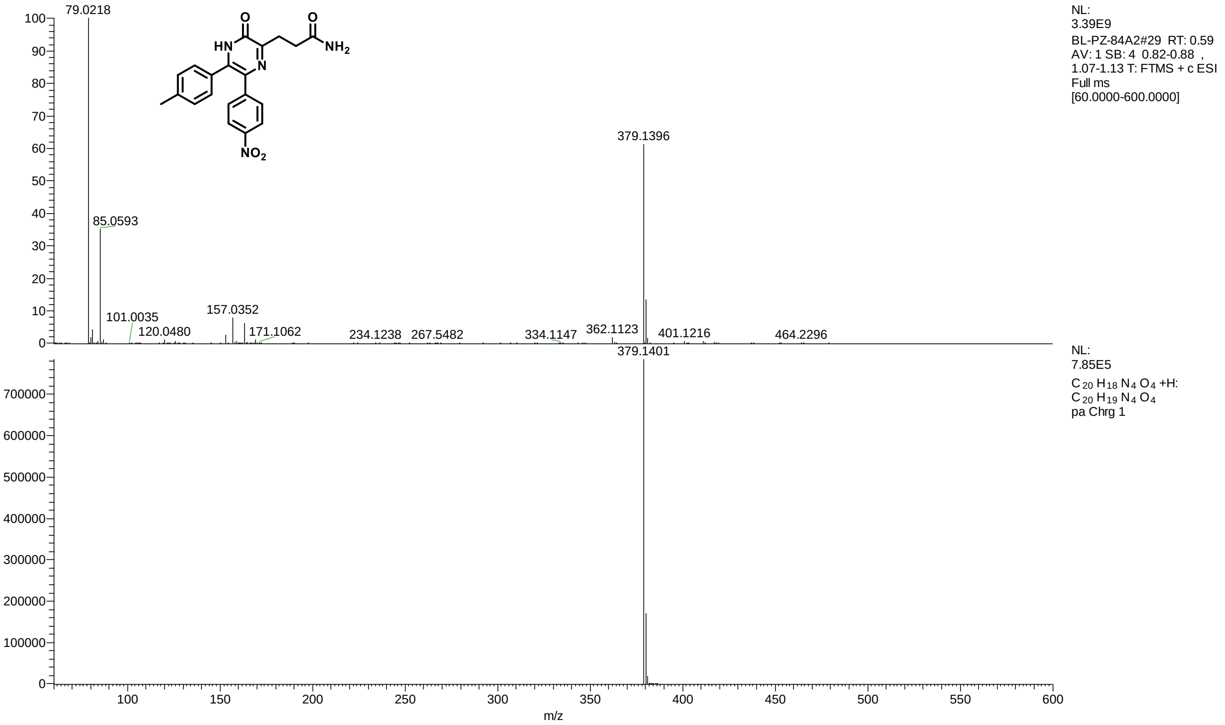

ESI-

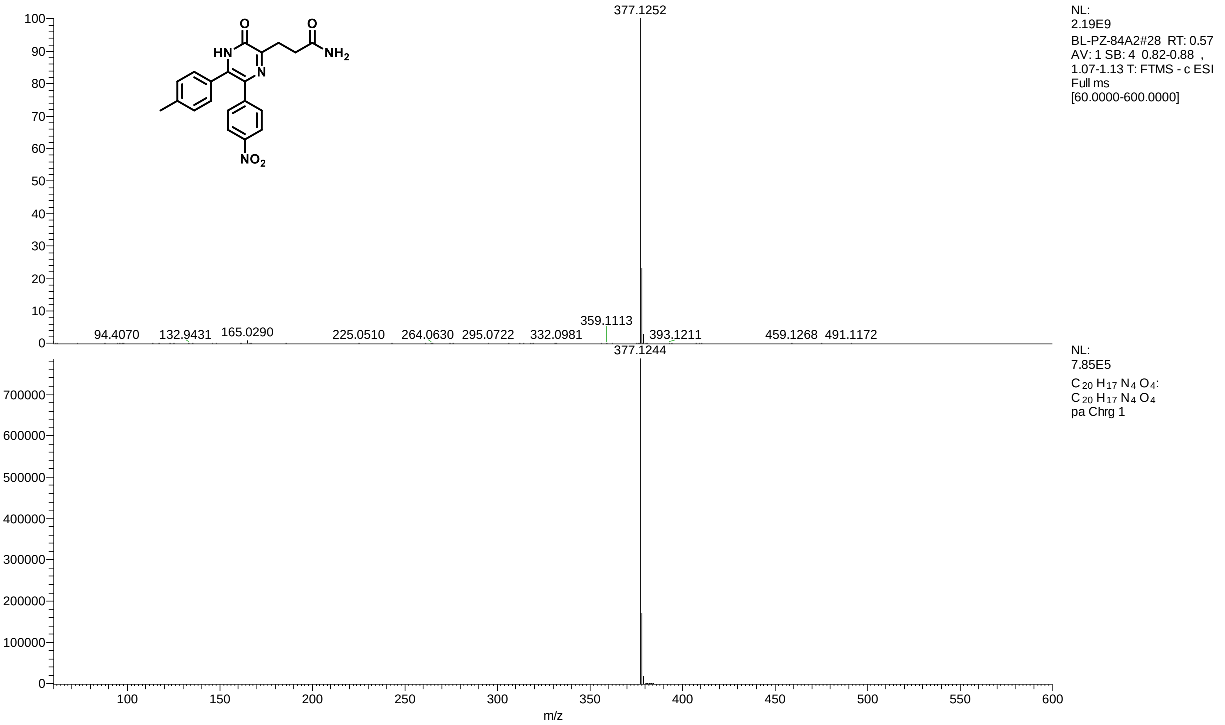

1-(3-(6-(4-nitrophenyl)-3-oxo-5-(*p*-tolyl)-3,4-dihydropyrazin-2-yl)propyl)guanidine (7j)

ESI+

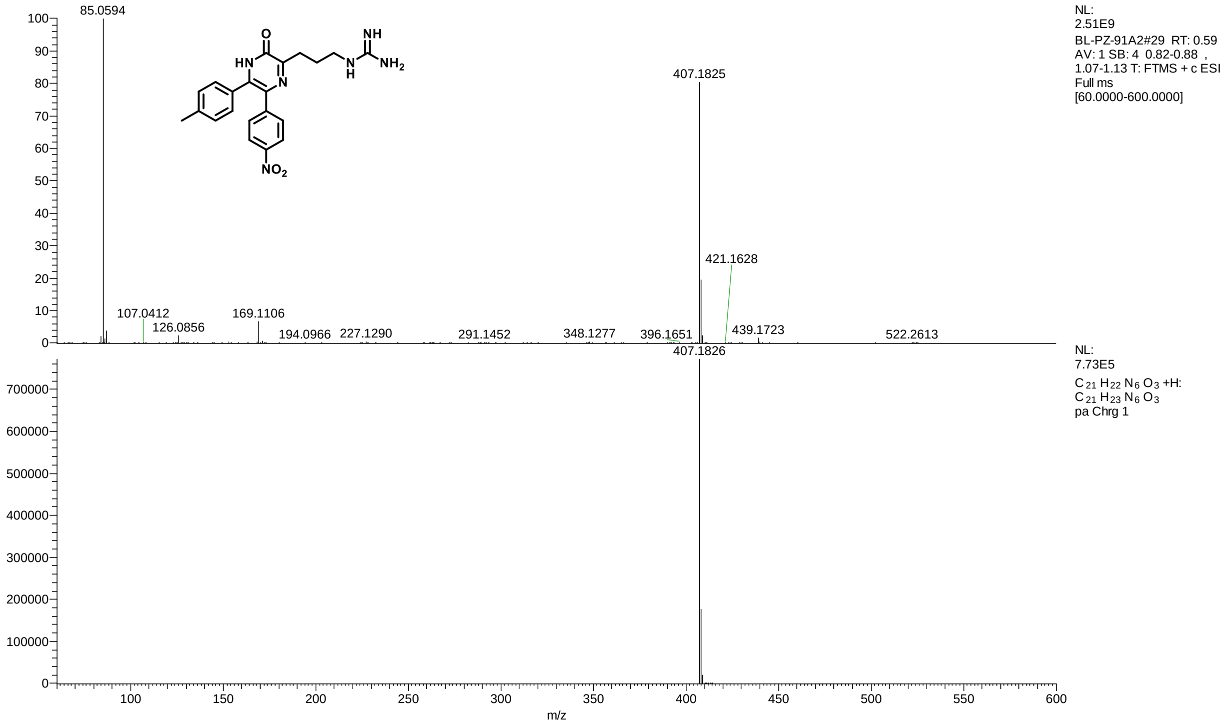

ESI-

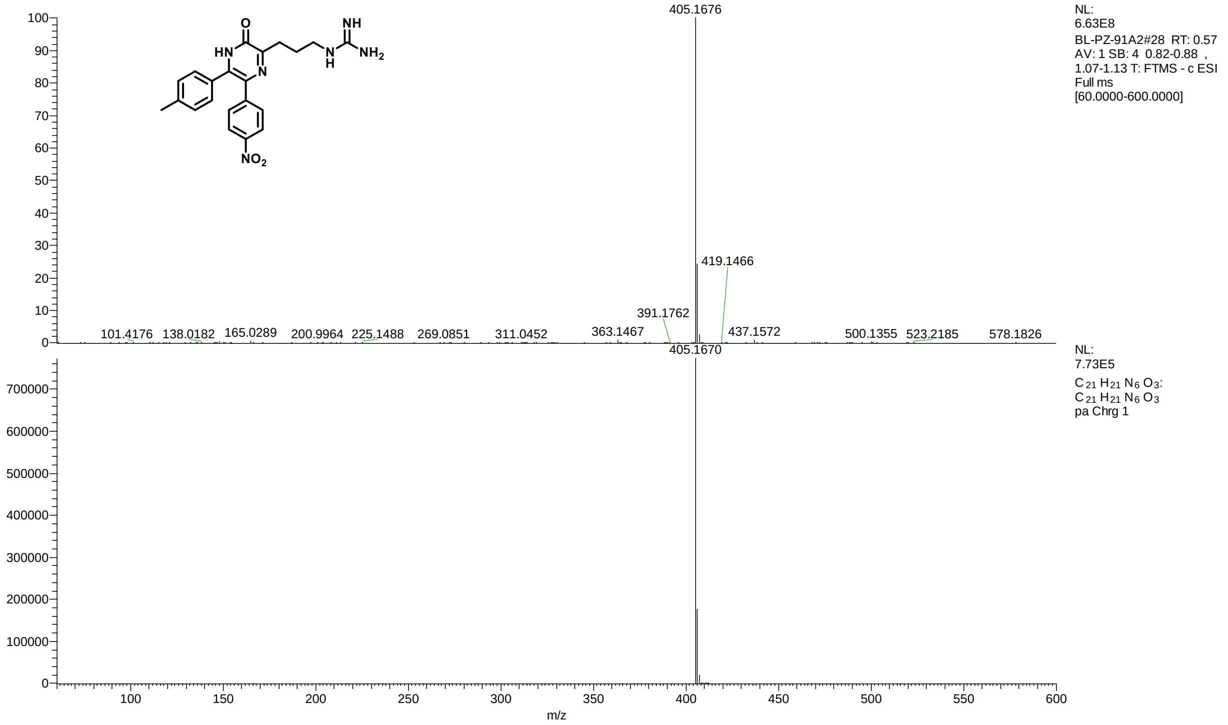

6-(4-amino-3,5-dichlorophenyl)-3-methyl-5-(4-nitrophenyl)pyrazin-2(1*H*)-one (7k)

ESI+

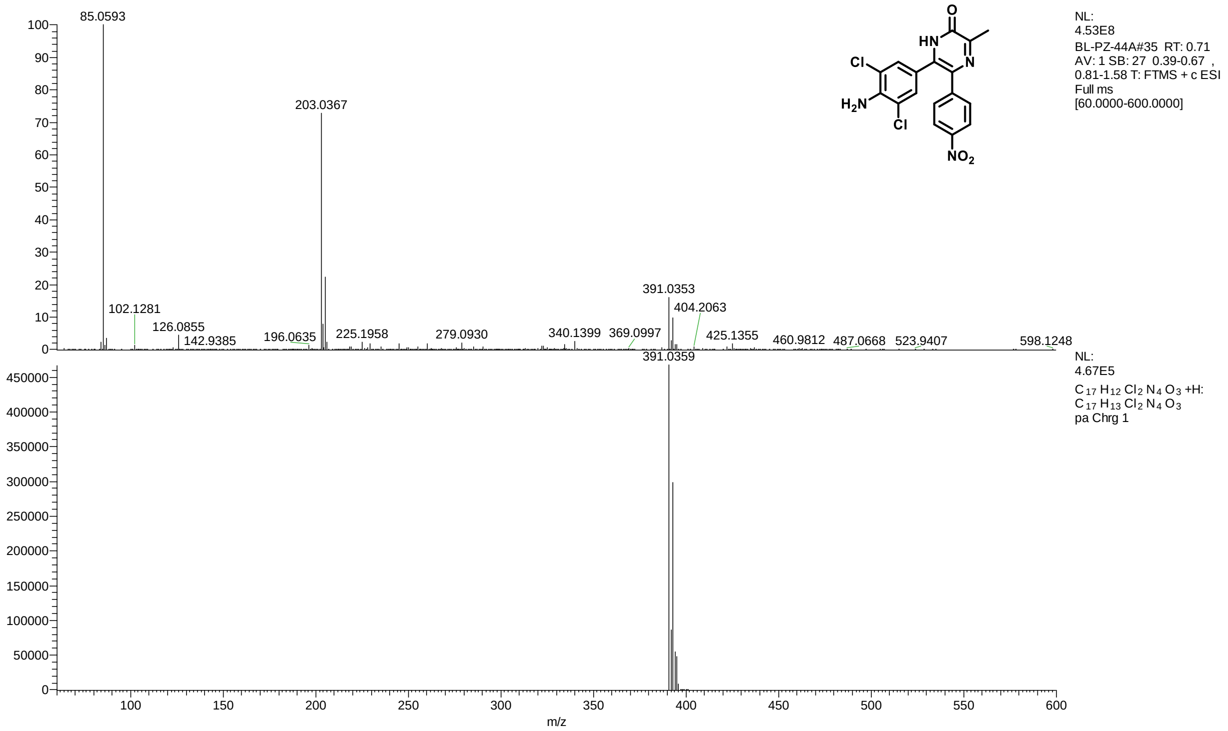

Zoom

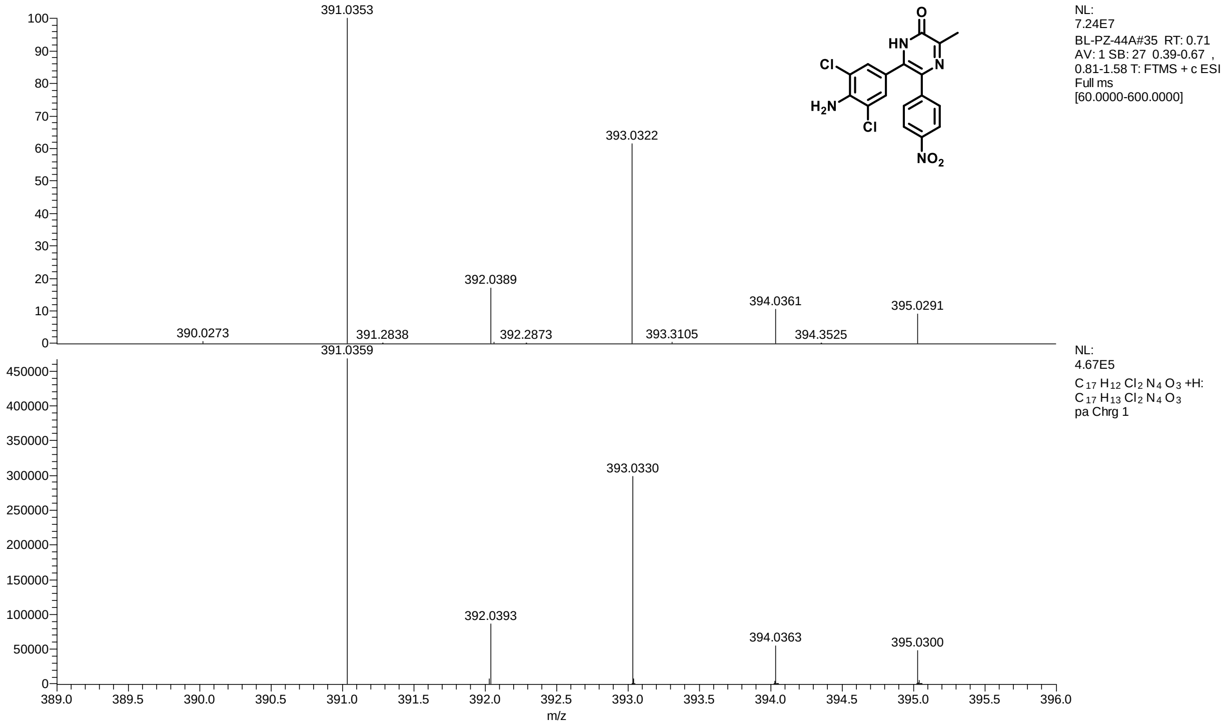

ESI-

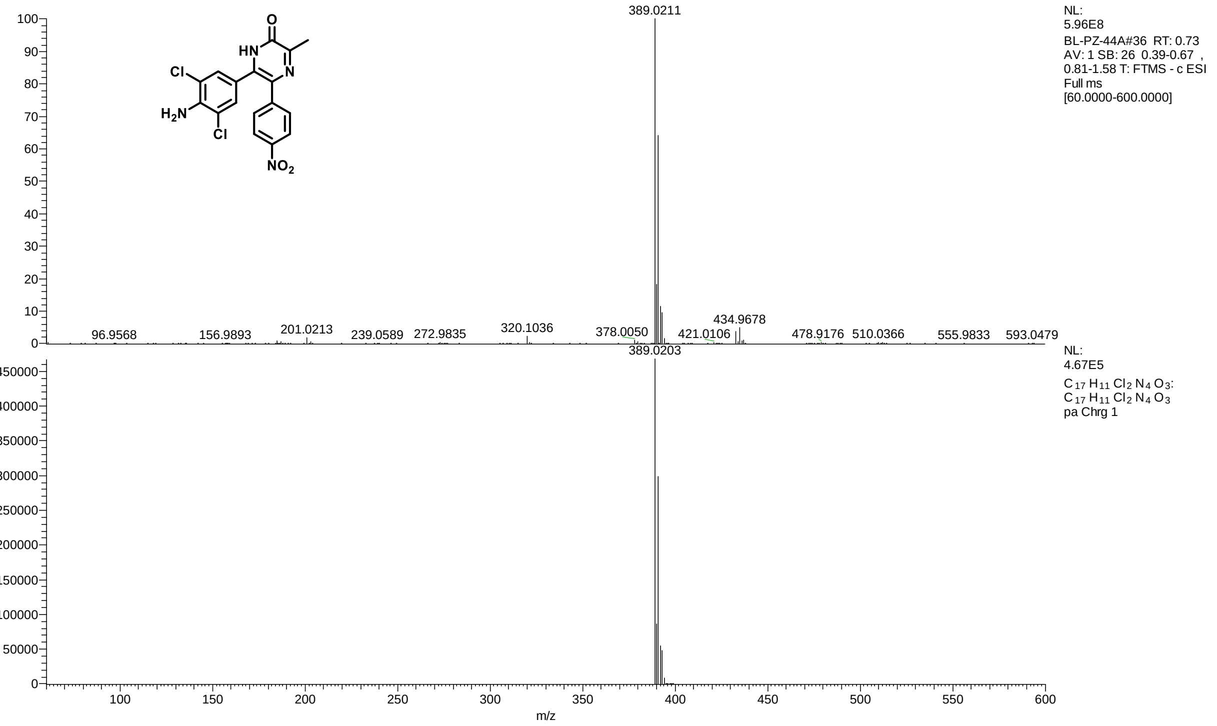
389.0211
NL:
5.96E8
BL-PZ-44A#36 RT: 0.73
AV: 1 SB: 26 0.39-0.67 ,
0.81-1.58 T: FTMS - c ESI
Full ms
[60.0000-600.0000]
96.9568
156.9893
201.0213
239.0589
272.9835
320.1036
378.0050
421.0106
434.9678
478.9176
510.0366
555.9833
593.0479
389.0203
NL:
4.67E5
C 17 H11 Cl2 N4 O3:
C 17 H11 Cl2 N4 O3
pa Chrg 1
m/z

Zoom

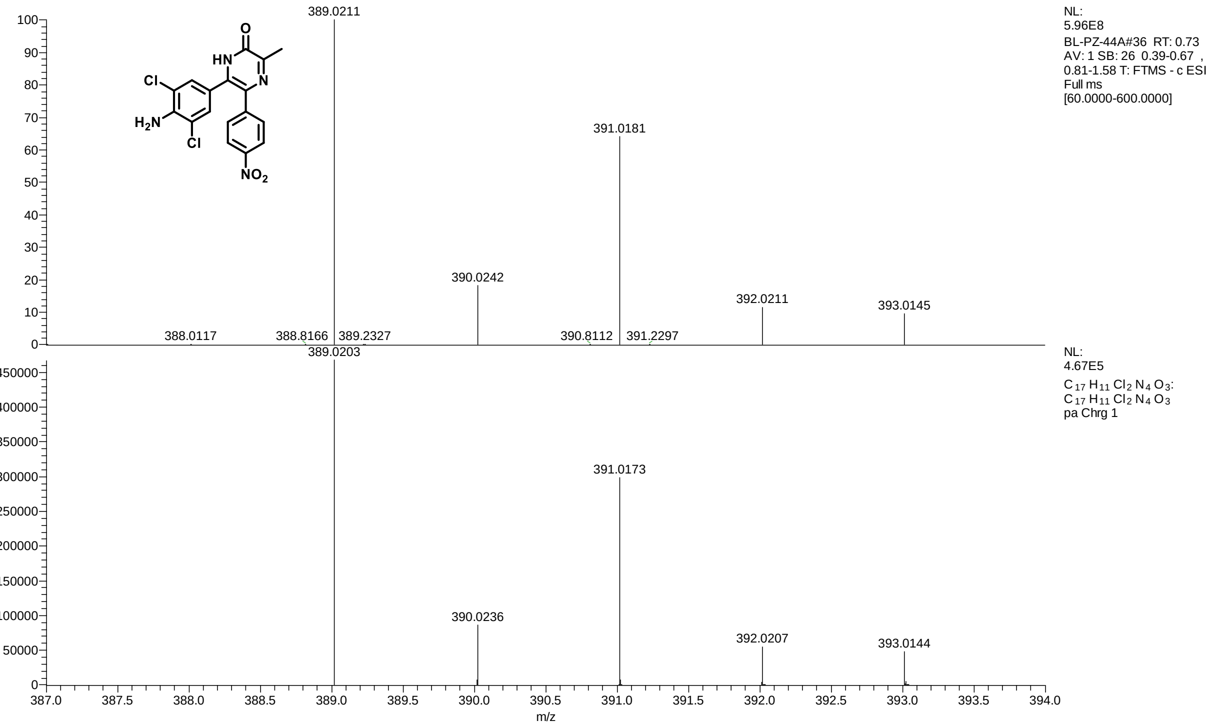
389.0211
NL:
5.96E8
BL-PZ-44A#36 RT: 0.73
AV: 1 SB: 26 0.39-0.67 ,
0.81-1.58 T: FTMS - c ESI
Full ms
[60.0000-600.0000]
391.0181
390.0242
392.0211
393.0145
388.0117
388.8166
389.2327
390.8112
391.2297
389.0203
NL:
4.67E5
C 17 H11 Cl2 N4 O3:
C 17 H11 Cl2 N4 O3
pa Chrg 1
391.0173
390.0236
392.0207
393.0144
m/z

3-((1*H*-indol-3-yl)methyl)-6-(4-methoxyphenyl)-5-(4-nitrophenyl)pyrazin-2(1*H*)-one (7l)

ESI+

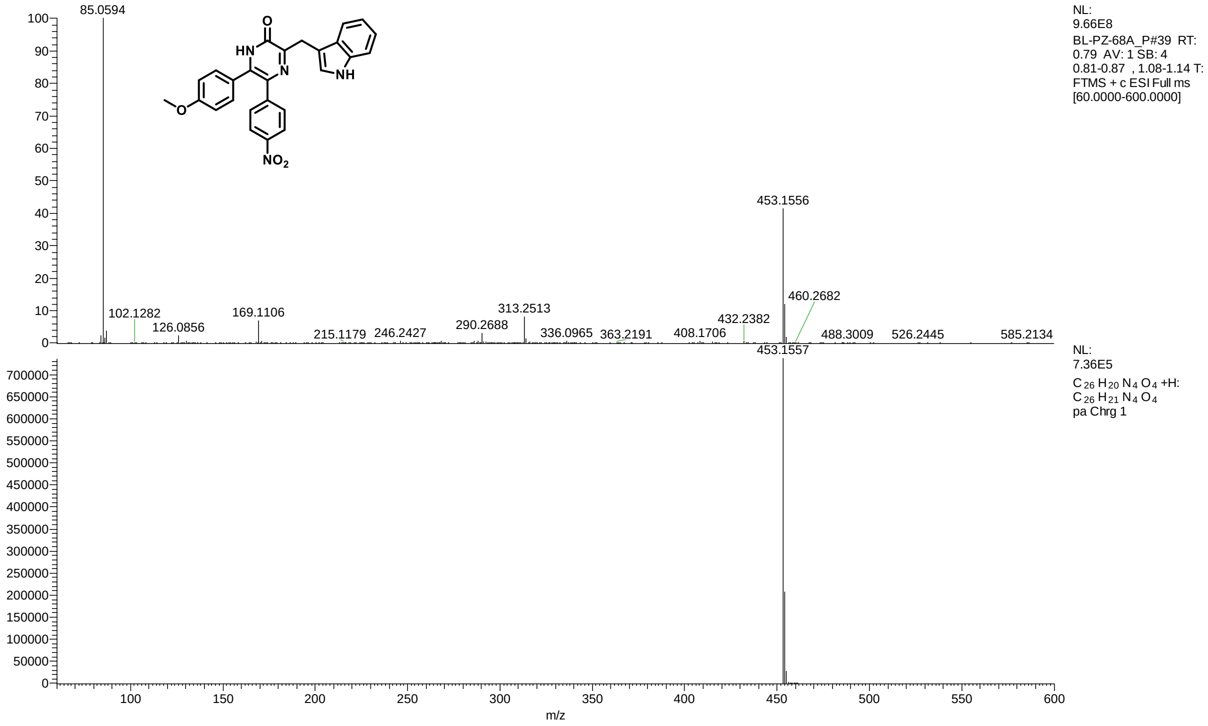

ESI-

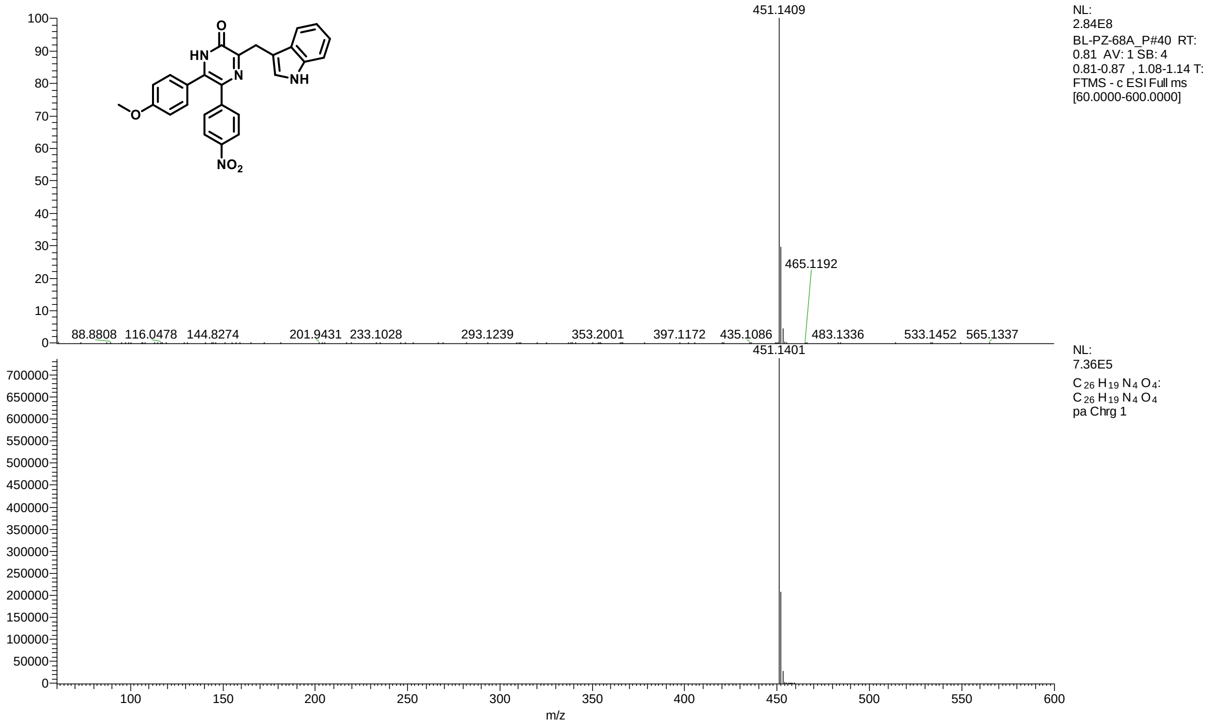

HPLC-UV spectra of crude final compounds (solvent peaks not integrated).

6-carbamoyl-2-(4-nitrophenyl)-3-(*p*-tolyl)isonicotinic acid (6a)

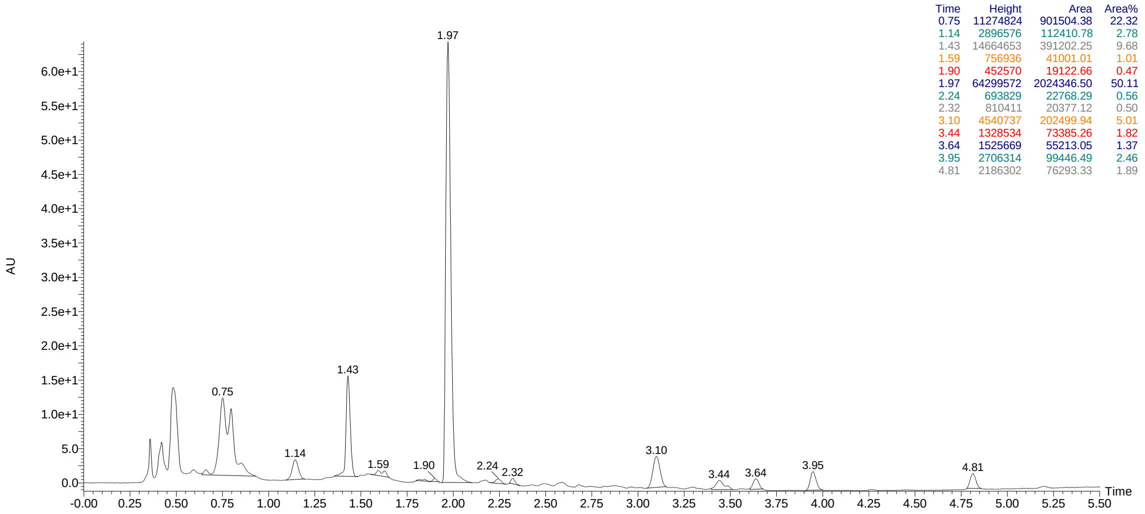

Time	Height	Area	Area%
0.75	11274824	901504.38	22.32
1.14	2896576	112410.78	2.78
1.43	14664653	391202.25	9.68
1.59	756936	41001.01	1.01
1.90	452570	19122.66	0.47
1.97	64299572	2024346.50	50.11
2.24	693829	22768.29	0.56
2.32	810411	20377.12	0.50
3.10	4540737	202499.94	5.01
3.44	1328534	73385.26	1.82
3.64	1525669	55213.05	1.37
3.95	2706314	99446.49	2.46
4.81	2186302	76293.33	1.89

6-carbamoyl-3-(4-methoxyphenyl)-2-(4-nitrophenyl)isonicotinic acid (6b)

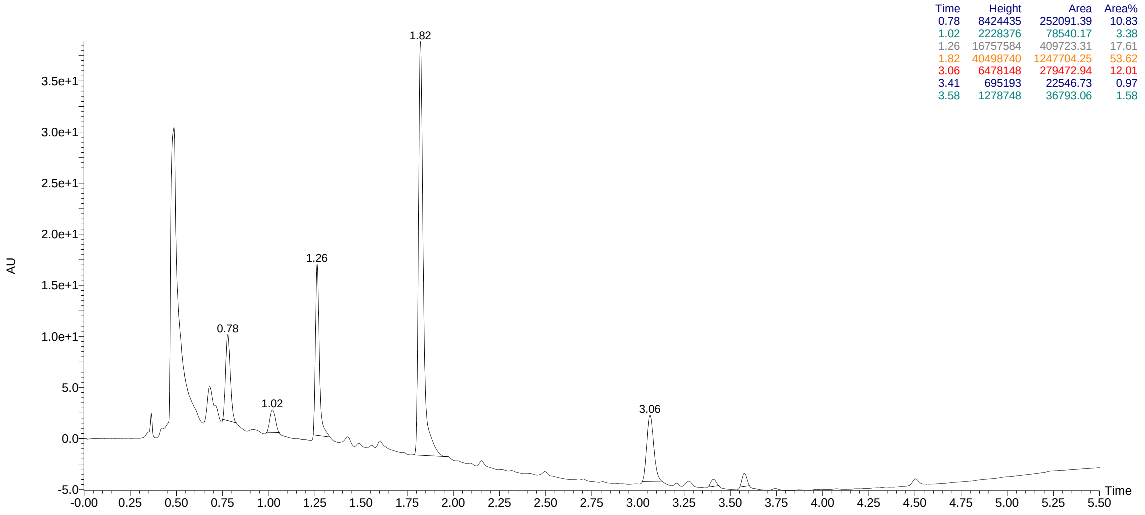

Time	Height	Area	Area%
0.78	8424435	252091.39	10.83
1.02	2228376	78540.17	3.38
1.26	16757584	409723.31	17.61
1.82	40498740	1247704.25	53.62
3.06	6478148	279472.94	12.01
3.41	695193	22546.73	0.97
3.58	1278748	36793.06	1.58

6-(3-carbamoylpyrrolidine-1-carbonyl)-2-(4-nitrophenyl)-3-(p-tolyl)isonicotinic acid (6c)

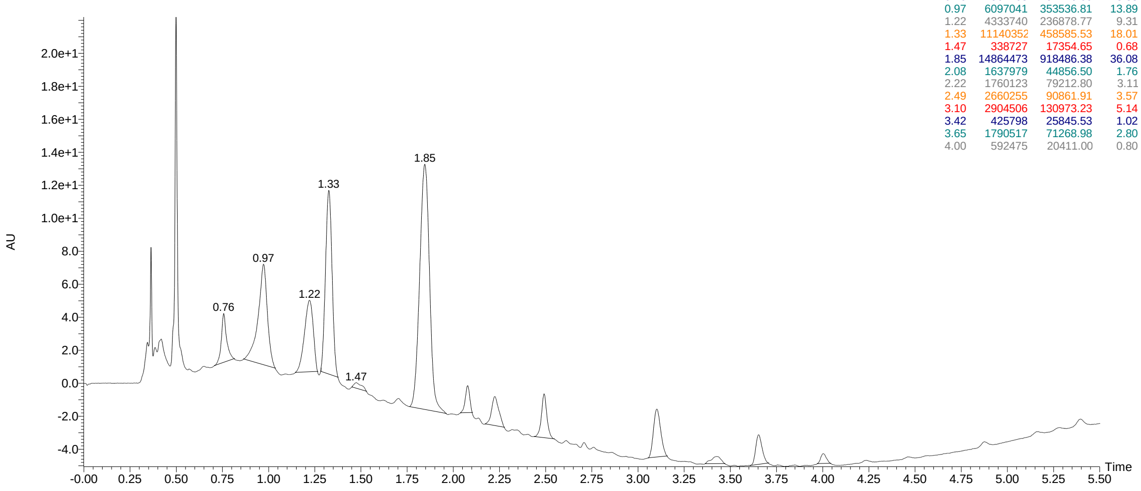

Time	Height	Area	Area%
0.76	2967263	97416.66	3.83
0.97	6097041	353536.81	13.89
1.22	4333740	236878.77	9.31
1.33	11140352	458585.53	18.01
1.47	338727	17354.65	0.68
1.85	14864473	918486.38	36.08
2.08	1637979	44856.50	1.76
2.22	1760123	79212.80	3.11
2.49	2660255	90861.91	3.57
3.10	2904506	130973.23	5.14
3.42	425798	25845.53	1.02
3.65	1790517	71268.98	2.80
4.00	592475	20411.00	0.80

3-(4-fluorophenyl)-2-(4-nitrophenyl)-6-(piperazine-1-carbonyl)isonicotinic acid (6d)

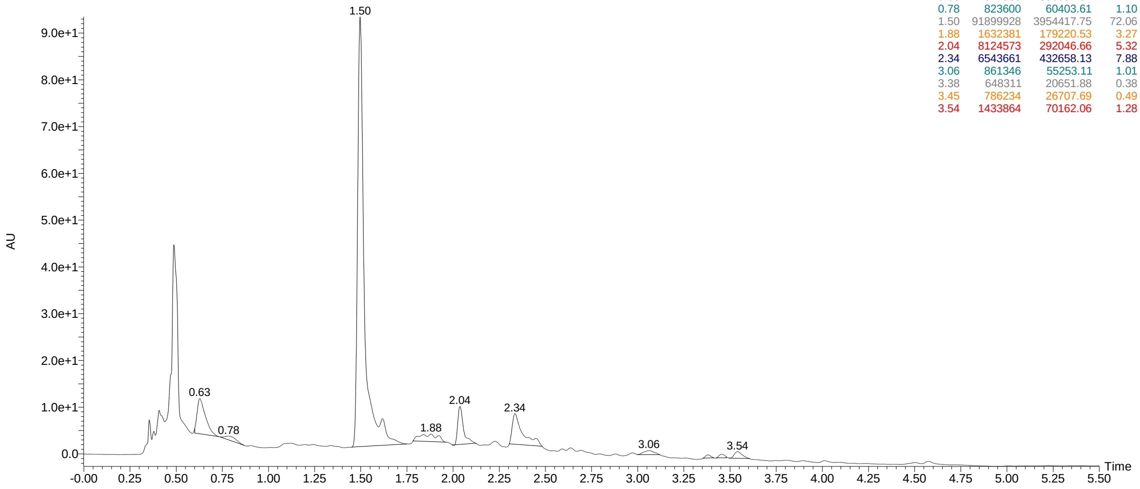

Time	Height	Area	Area%
0.63	7527863	396092.91	7.22
0.78	823600	60403.61	1.10
1.50	91899928	3954417.75	72.06
1.88	1632381	179220.53	3.27
2.04	8124573	292046.66	5.32
2.34	6543661	432658.13	7.88
3.06	861346	55253.11	1.01
3.38	648311	20651.88	0.38
3.45	786234	26707.69	0.49
3.54	1433864	70162.06	1.28

2-(4-nitrophenyl)-6-(piperazine-1-carbonyl)-3-(*p*-tolyl)isonicotinic acid (6e)

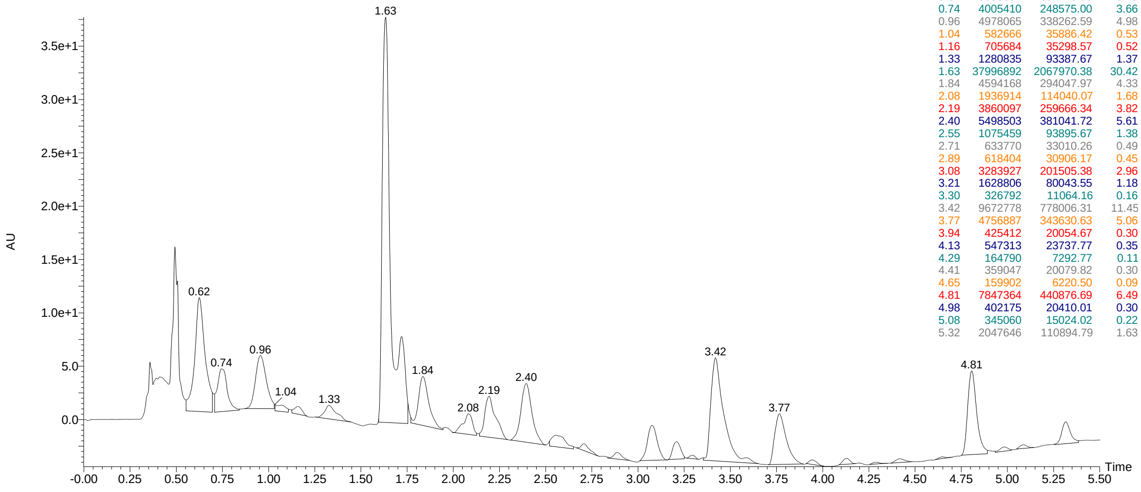

Time	Height	Area	Area%
0.62	10685594	692423.06	10.19
0.74	4005410	248575.00	3.66
0.96	4978065	338262.59	4.98
1.04	582666	35886.42	0.53
1.16	705684	35298.57	0.52
1.33	1280835	93387.67	1.37
1.63	37996892	2067970.38	30.42
1.84	4594168	294047.97	4.33
2.08	1936914	114040.07	1.68
2.19	3860097	259666.34	3.82
2.40	5498503	381041.72	5.61
2.55	1075459	93895.67	1.38
2.71	633770	33010.26	0.49
2.89	618404	30906.17	0.45
3.08	3283927	201505.38	2.96
3.21	1628806	80043.55	1.18
3.30	326792	11064.16	0.16
3.42	9672778	778006.31	11.45
3.77	4756887	343630.63	5.06
3.94	425412	20054.67	0.30
4.13	547313	23737.77	0.35
4.29	164790	7292.77	0.11
4.41	359047	20079.82	0.30
4.65	159902	6220.50	0.09
4.81	7847364	440876.69	6.49
4.98	402175	20410.01	0.30
5.08	345060	15024.02	0.22
5.32	2047646	110894.79	1.63

3-(3-methoxyphenyl)-2-(4-nitrophenyl)-6-(piperazine-1-carbonyl)isonicotinic acid (6f)

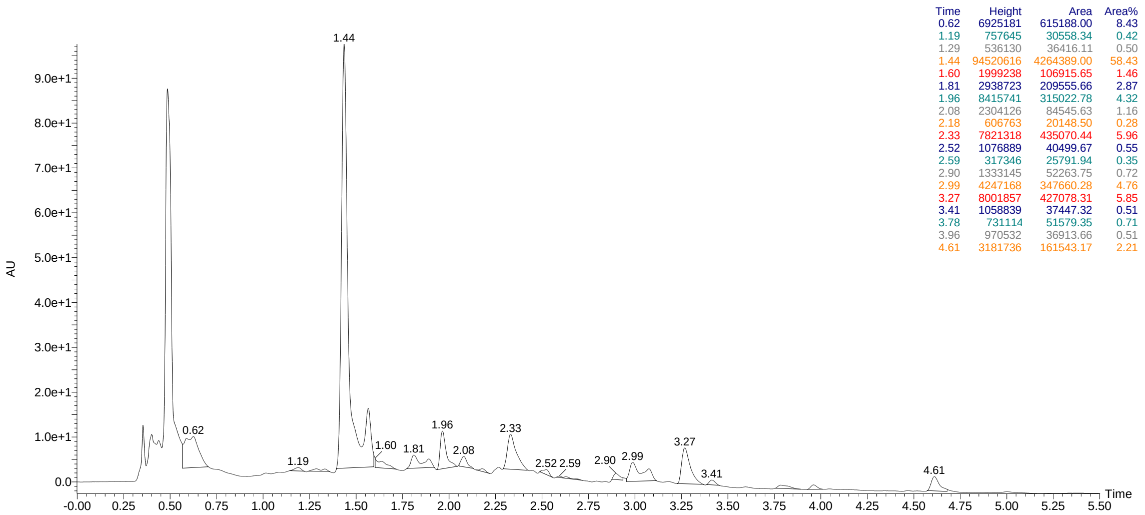

Time	Height	Area	Area%
0.62	6925181	615188.00	8.43
1.19	757645	30558.34	0.42
1.29	536130	36416.11	0.50
1.44	94520616	4264389.00	58.43
1.60	1999238	106915.65	1.46
1.81	2938723	209555.66	2.87
1.96	8415741	315022.78	4.32
2.08	2304126	84545.63	1.16
2.18	606763	20148.50	0.28
2.33	7821318	435070.44	5.96
2.52	1076889	40499.67	0.55
2.59	317346	25791.94	0.35
2.90	1333145	52263.75	0.72
2.99	4247168	347660.28	4.76
3.27	8001857	427078.31	5.85
3.41	1058839	37447.32	0.51
3.78	731114	51579.35	0.71
3.96	970532	36913.66	0.51
4.61	3181736	161543.17	2.21

3-(4-amino-3,5-dichlorophenyl)-2-(4-nitrophenyl)-6-(piperazine-1-carbonyl)isonicotinic acid (6g)

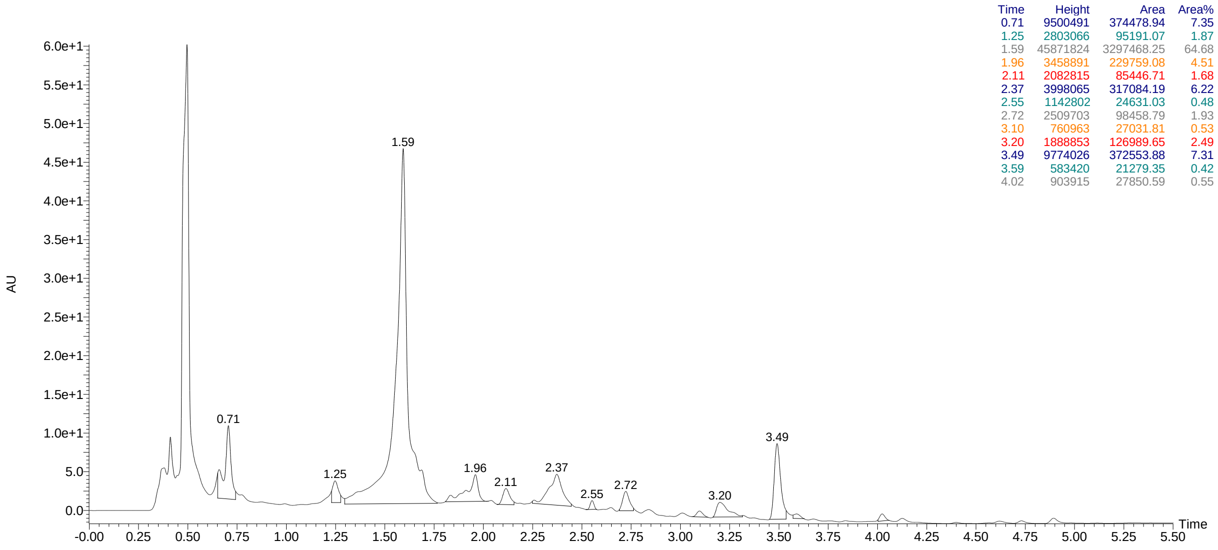

Time	Height	Area	Area%
0.71	9500491	374478.94	7.35
1.25	2803066	95191.07	1.87
1.59	45871824	3297468.25	64.68
1.96	3458891	229759.08	4.51
2.11	2082815	85446.71	1.68
2.37	3998065	317084.19	6.22
2.55	1142802	24631.03	0.48
2.72	2509703	98458.79	1.93
3.10	760963	27031.81	0.53
3.20	1888853	126989.65	2.49
3.49	9774026	372553.88	7.31
3.59	583420	21279.35	0.42
4.02	903915	27850.59	0.55

ethyl 3-(3-methoxyphenyl)-2-(4-nitrophenyl)-6-(piperazine-1-carbonyl)isonicotinate (6h)

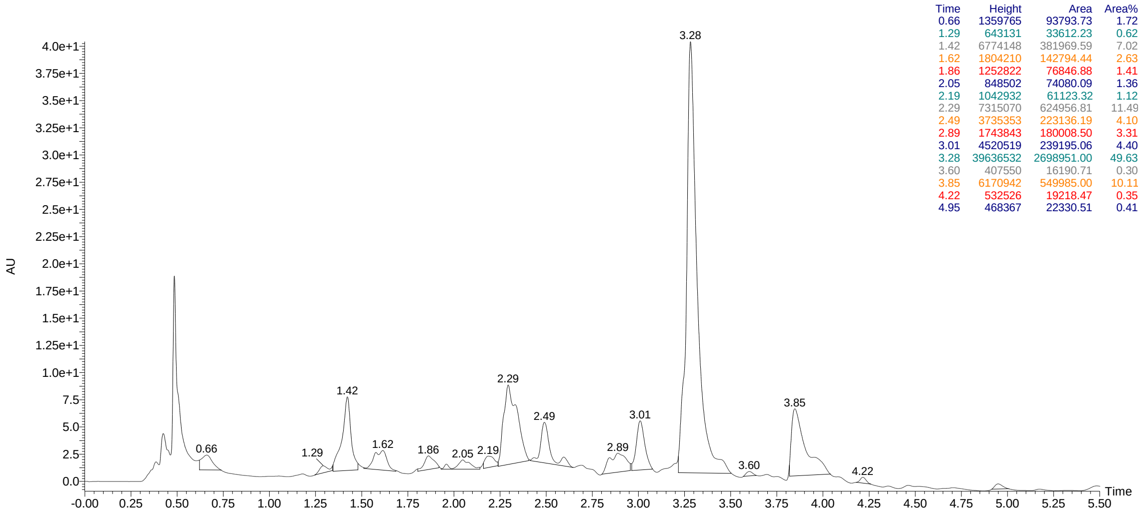

Time	Height	Area	Area%
0.66	1359765	93793.73	1.72
1.29	643131	33612.23	0.62
1.42	6774148	381969.59	7.02
1.62	1804210	142794.44	2.63
1.86	1252822	76846.88	1.41
2.05	848502	74080.09	1.36
2.19	1042932	61123.32	1.12
2.29	7315070	624956.81	11.49
2.49	3735353	223136.19	4.10
2.89	1743843	180008.50	3.31
3.01	4520519	239195.06	4.40
3.28	39636532	2698951.00	49.63
3.60	407550	16190.71	0.30
3.85	6170942	549985.00	10.11
4.22	532526	19218.47	0.35
4.95	468367	22330.51	0.41

(3-(3-methoxyphenyl)-2-(4-nitrophenyl)-6-(piperazine-1-carbonyl)pyridin-4-yl)(piperidin-1-yl)methanone (6i)

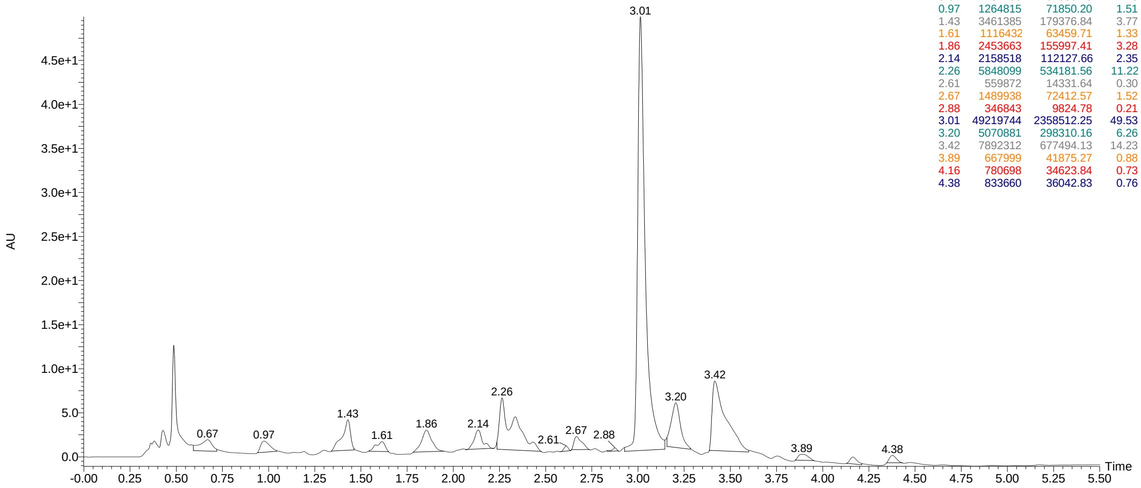

Time	Height	Area	Area%
0.67	1287200	101553.43	2.13
0.97	1264815	71850.20	1.51
1.43	3461385	179376.84	3.77
1.61	1116432	63459.71	1.33
1.86	2453663	155997.41	3.28
2.14	2158518	112127.66	2.35
2.26	5848099	534181.56	11.22
2.61	559872	14331.64	0.30
2.67	1489938	72412.57	1.52
2.88	346843	9824.78	0.21
3.01	49219744	2358512.25	49.53
3.20	5070881	298310.16	6.26
3.42	7892312	677494.13	14.23
3.89	667999	41875.27	0.88
4.16	780698	34623.84	0.73
4.38	833660	36042.83	0.76

2-(4-nitrophenyl)-6-(piperazine-1-carbonyl)-3-(thiophen-3-yl)isonicotinic acid (6j)

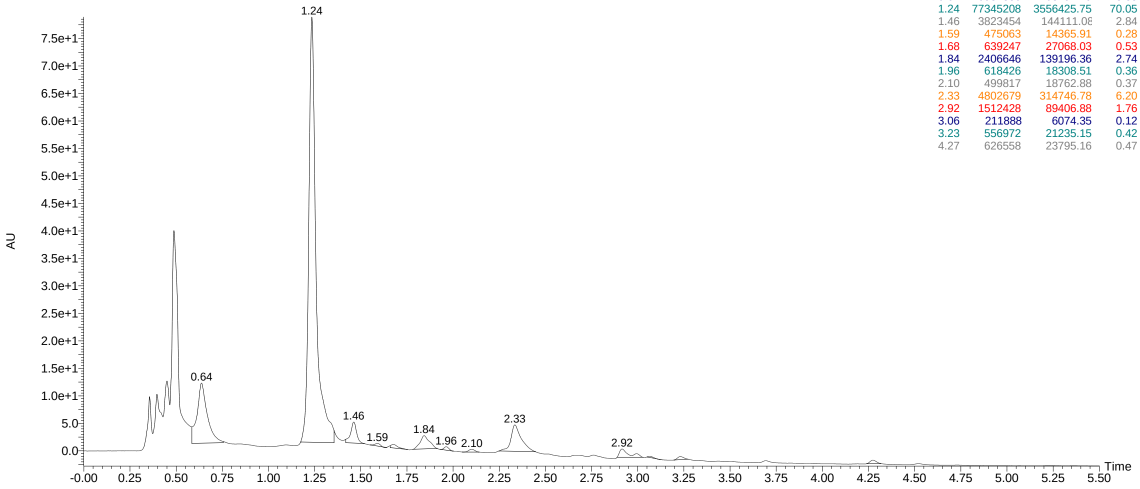

Time	Height	Area	Area%
0.64	10934850	703291.19	13.85
1.24	77345208	3556425.75	70.05
1.46	3823454	144111.08	2.84
1.59	475063	14365.91	0.28
1.68	639247	27068.03	0.53
1.84	2406646	139196.36	2.74
1.96	618426	18308.51	0.36
2.10	499817	18762.88	0.37
2.33	4802679	314746.78	6.20
2.92	1512428	89406.88	1.76
3.06	211888	6074.35	0.12
3.23	556972	21235.15	0.42
4.27	626558	23795.16	0.47

3-(3-methoxyphenyl)-2-(4-nitrophenyl)-6-(piperazine-1-carbonyl)-N-propylisonicotinamide (6k)

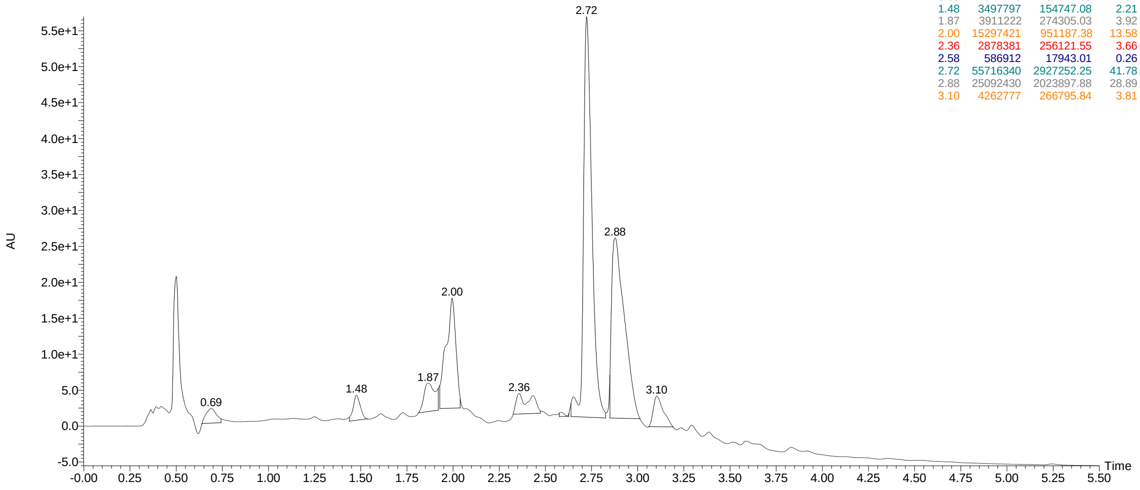

Time	Height	Area	Area%
0.69	2044173	134040.89	1.91
1.48	3497797	154747.08	2.21
1.87	3911222	274305.03	3.92
2.00	15297421	951187.38	13.58
2.36	2878381	256121.55	3.66
2.58	586912	17943.01	0.26
2.72	55716340	2927252.25	41.78
2.88	25092430	2023897.88	28.89
3.10	4262777	266795.84	3.81

3-(4-methoxyphenyl)-2-(4-nitrophenyl)-6-(propylcarbamoyl)isonicotinic acid (6l)

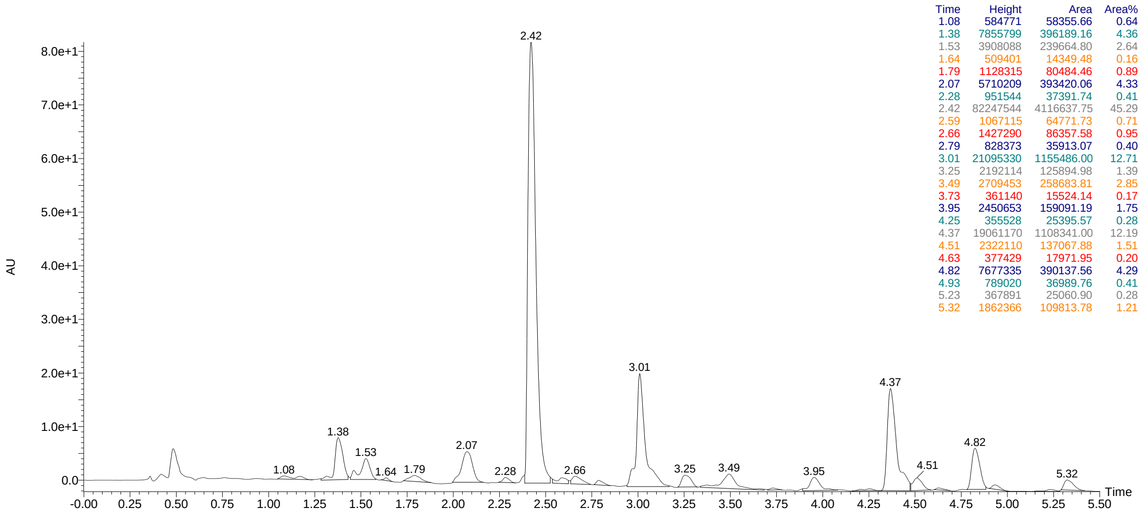

Time	Height	Area	Area%
1.08	584771	58355.66	0.64
1.38	7855799	396189.16	4.36
1.53	3908088	239664.80	2.64
1.64	509401	14349.48	0.16
1.79	1128315	80484.46	0.89
2.07	5710209	393420.06	4.33
2.28	951544	37391.74	0.41
2.42	82247544	4116637.75	45.29
2.59	1067115	64771.73	0.71
2.66	1427290	86357.58	0.95
2.79	828373	35913.07	0.40
3.01	21095330	1155486.00	12.71
3.25	2192114	125894.98	1.39
3.49	2709453	258683.81	2.85
3.73	361140	15524.14	0.17
3.95	2450653	159091.19	1.75
4.25	355528	25395.57	0.28
4.37	19061170	1108341.00	12.19
4.51	2322110	137067.88	1.51
4.63	377429	17971.95	0.20
4.82	7677335	390137.56	4.29
4.93	789020	36989.76	0.41
5.23	367891	25060.90	0.28
5.32	1862366	109813.78	1.21

3-(6-(4-nitrophenyl)-3-oxo-5-(*p*-tolyl)-3,4-dihydropyrazin-2-yl)propanoic acid (7a)

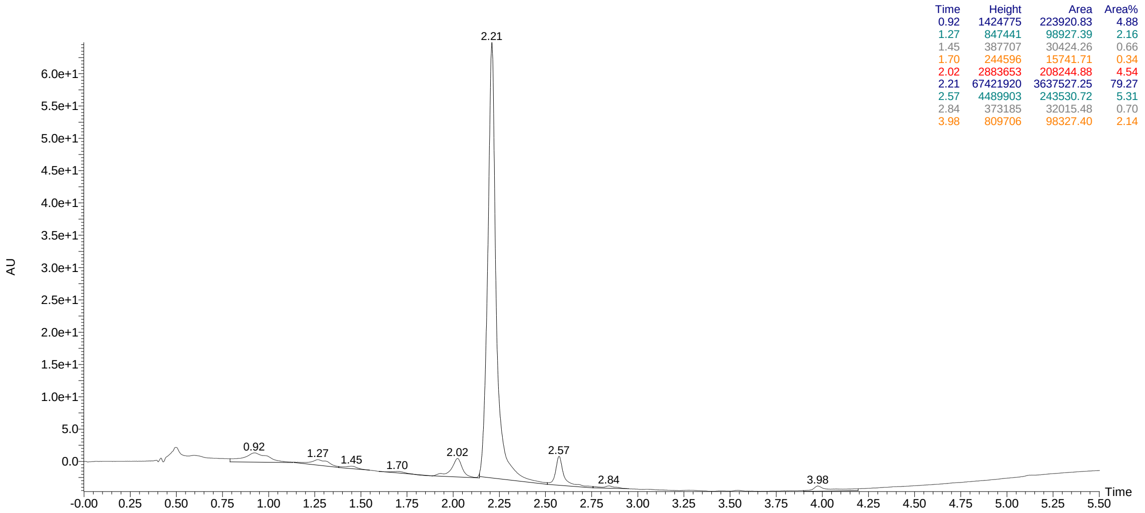

Time	Height	Area	Area%
0.92	1424775	223920.83	4.88
1.27	847441	98927.39	2.16
1.45	387707	30424.26	0.66
1.70	244596	15741.71	0.34
2.02	2883653	208244.88	4.54
2.21	67421920	3637527.25	79.27
2.57	4489903	243530.72	5.31
2.84	373185	32015.48	0.70
3.98	809706	98327.40	2.14

Methyl 3-(5-(4-methoxyphenyl)-6-(4-nitrophenyl)-3-oxo-3,4-dihydropyrazin-2-yl)propanoate (7b)

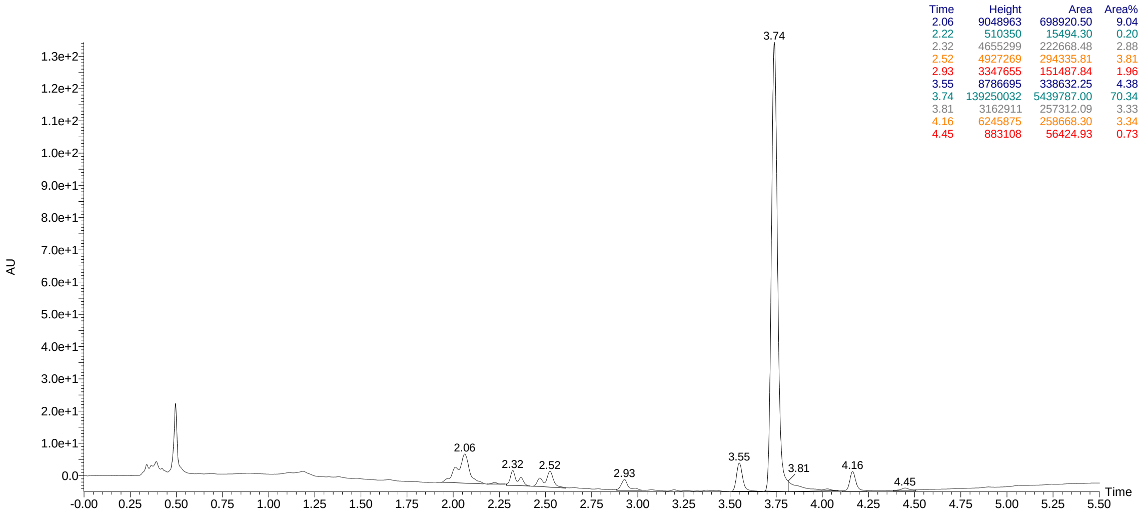

Time	Height	Area	Area%
2.06	9048963	698920.50	9.04
2.22	510350	15494.30	0.20
2.32	4655299	222668.48	2.88
2.52	4927269	294335.81	3.81
2.93	3347655	151487.84	1.96
3.55	8786695	338632.25	4.38
3.74	139250032	5439787.00	70.34
3.81	3162911	257312.09	3.33
4.16	6245875	258668.30	3.34
4.45	883108	56424.93	0.73

5-(4-nitrophenyl)-6-(*p*-tolyl)pyrazin-2(1*H*)-one (7c)

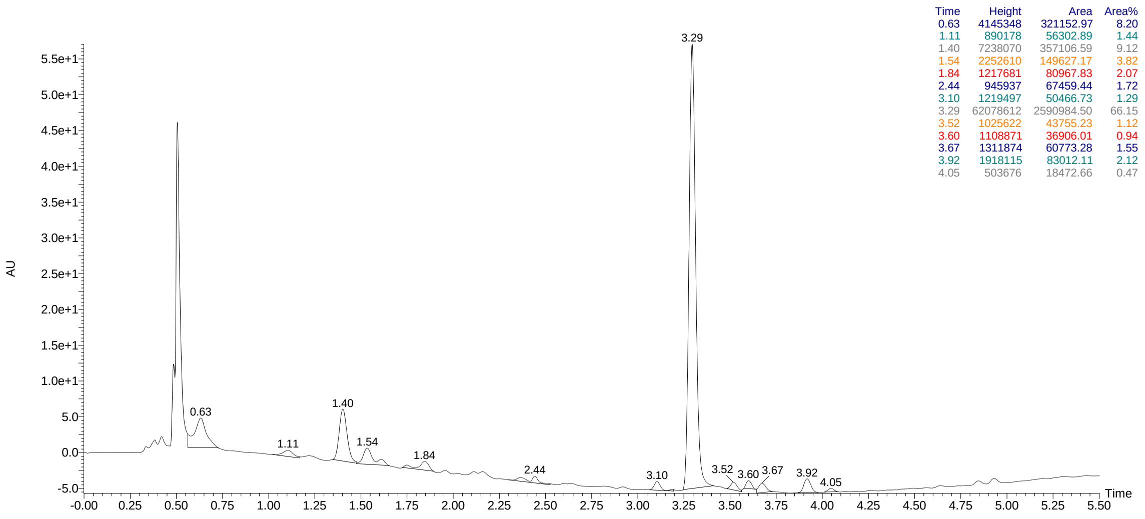

Time	Height	Area	Area%
0.63	4145348	321152.97	8.20
1.11	890178	56302.89	1.44
1.40	7238070	357106.59	9.12
1.54	2252610	149627.17	3.82
1.84	1217681	80967.83	2.07
2.44	945937	67459.44	1.72
3.10	1219497	50466.73	1.29
3.29	62078612	2590984.50	66.15
3.52	1025622	43755.23	1.12
3.60	1108871	36906.01	0.94
3.67	1311874	60773.28	1.55
3.92	1918115	83012.11	2.12
4.05	503676	18472.66	0.47

3-methyl-5-(4-nitrophenyl)-6-(*p*-tolyl)pyrazin-2(1*H*)-one (7d)

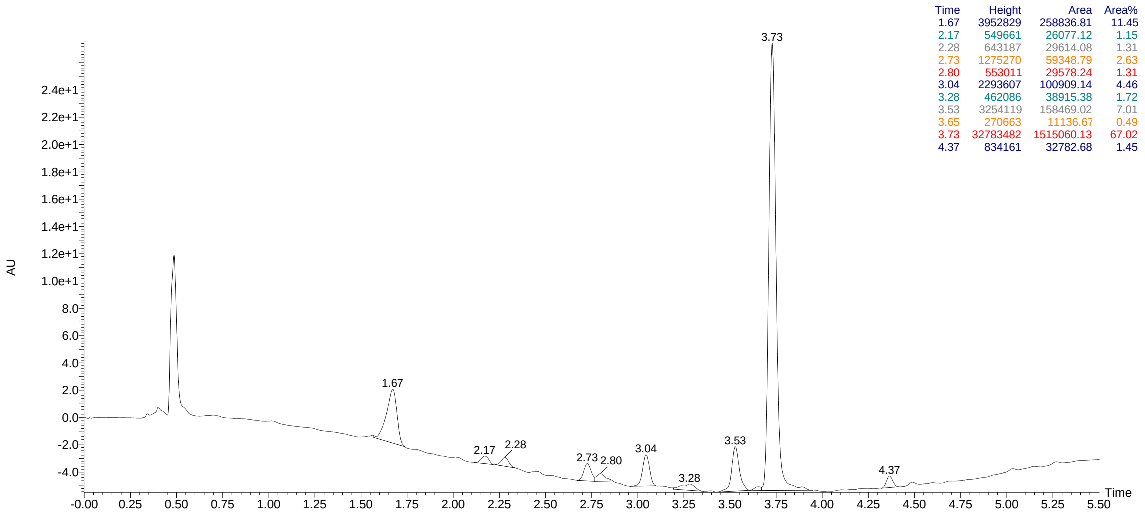

Time	Height	Area	Area%
1.67	3952829	258836.81	11.45
2.17	549661	26077.12	1.15
2.28	643187	29614.08	1.31
2.73	1275270	59348.79	2.63
2.80	553011	29578.24	1.31
3.04	2293607	100909.14	4.46
3.28	462086	38915.38	1.72
3.53	3254119	158469.02	7.01
3.65	270663	11136.67	0.49
3.73	32783482	1515060.13	67.02
4.37	834161	32782.68	1.45

3-isobutyl-5-(4-nitrophenyl)-6-(*p*-tolyl)pyrazin-2(1*H*)-one (7e)

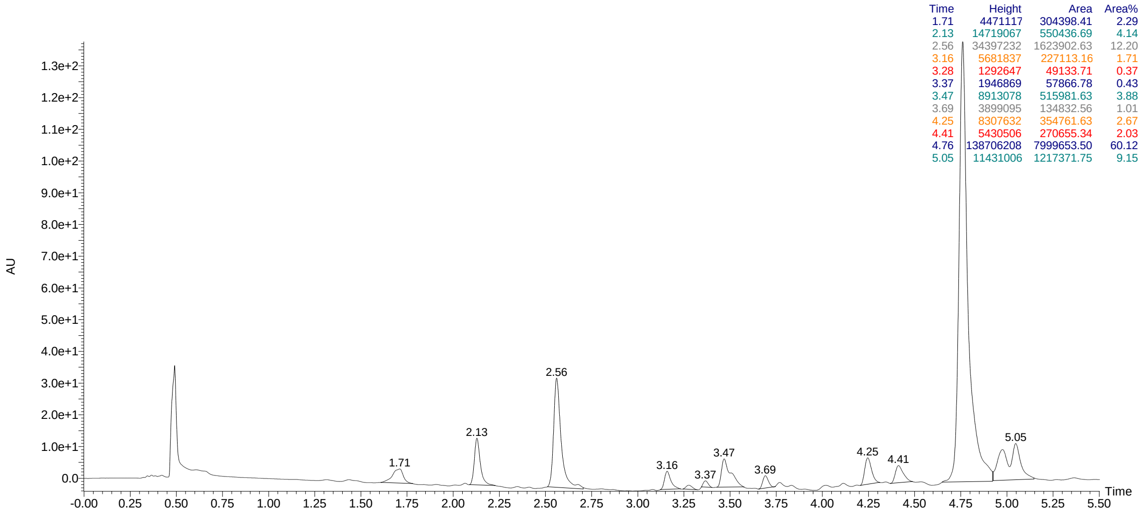

Time	Height	Area	Area%
1.71	4471117	304398.41	2.29
2.13	14719067	550436.69	4.14
2.56	34397232	1623902.63	12.20
3.16	5681837	227113.16	1.71
3.28	1292647	49133.71	0.37
3.37	1946869	57866.78	0.43
3.47	8913078	515981.63	3.88
3.69	3899095	134832.56	1.01
4.25	8307632	354761.63	2.67
4.41	5430506	270655.34	2.03
4.76	138706208	7999653.50	60.12
5.05	11431006	1217371.75	9.15

3-benzyl-5-(4-nitrophenyl)-6-(*p*-tolyl)pyrazin-2(1*H*)-one (7f)

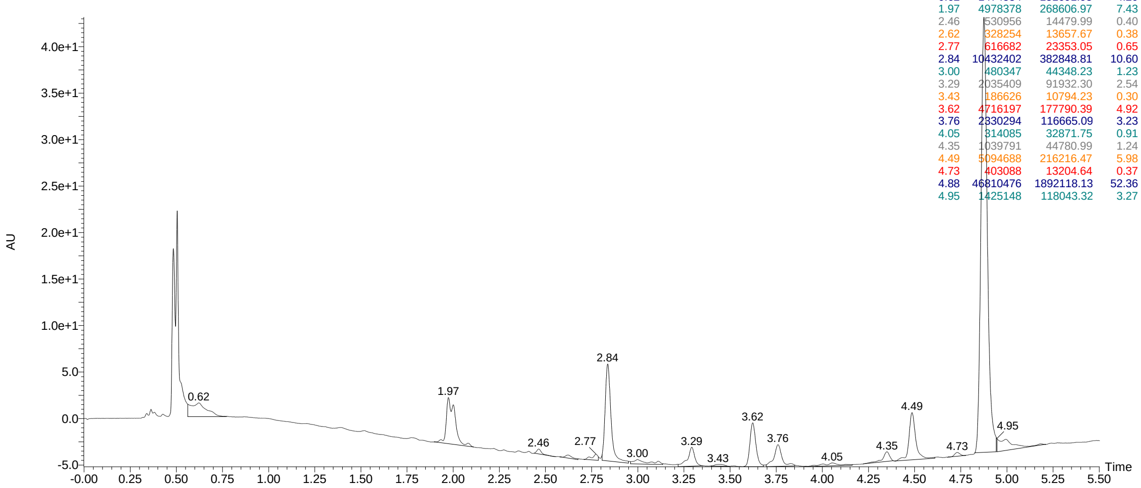

Time	Height	Area	Area%
0.62	1474554	151691.98	4.20
1.97	4978378	268606.97	7.43
2.46	530956	14479.99	0.40
2.62	328254	13657.67	0.38
2.77	616682	23353.05	0.65
2.84	10432402	382848.81	10.60
3.00	480347	44348.23	1.23
3.29	2035409	91932.30	2.54
3.43	186626	10794.23	0.30
3.62	4716197	177790.39	4.92
3.76	2330294	116665.09	3.23
4.05	314085	32871.75	0.91
4.35	1039791	44780.99	1.24
4.49	5094688	216216.47	5.98
4.73	403088	13204.64	0.37
4.88	46810476	1892118.13	52.36
4.95	1425148	118043.32	3.27

3-(4-hydroxybenzyl)-5-(4-nitrophenyl)-6-(*p*-tolyl)pyrazin-2(1*H*)-one (7g)

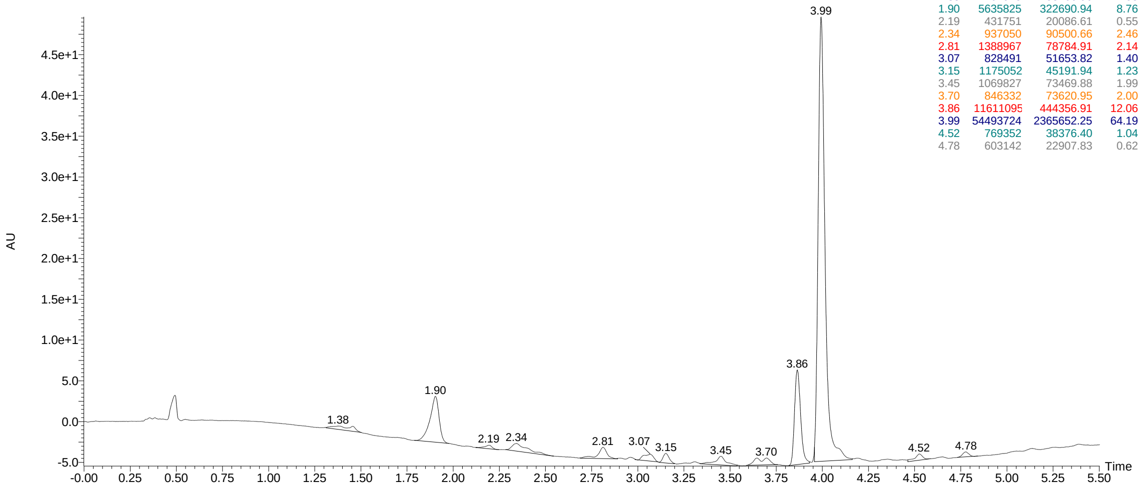

Time	Height	Area	Area%
1.38	407915	58166.96	1.58
1.90	5635825	322690.94	8.76
2.19	431751	20086.61	0.55
2.34	937050	90500.66	2.46
2.81	1388967	78784.91	2.14
3.07	828491	51653.82	1.40
3.15	1175052	45191.94	1.23
3.45	1069827	73469.88	1.99
3.70	846332	73620.95	2.00
3.86	11611095	444356.91	12.06
3.99	54493724	2365652.25	64.19
4.52	769352	38376.40	1.04
4.78	603142	22907.83	0.62

3-(2-(methylthio)ethyl)-5-(4-nitrophenyl)-6-(*p*-tolyl)pyrazin-2(1*H*)-one (7h)

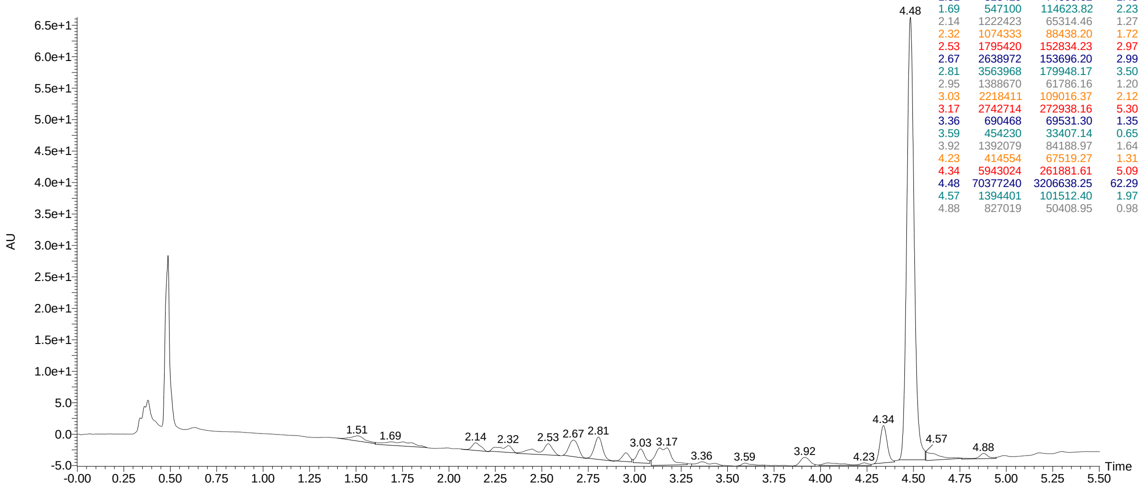

Time	Height	Area	Area%
1.51	823429	74600.62	1.45
1.69	547100	114623.82	2.23
2.14	1222423	65314.46	1.27
2.32	1074333	88438.20	1.72
2.53	1795420	152834.23	2.97
2.67	2638972	153696.20	2.99
2.81	3563968	179948.17	3.50
2.95	1388670	61786.16	1.20
3.03	2218411	109016.37	2.12
3.17	2742714	272938.16	5.30
3.36	690468	69531.30	1.35
3.59	454230	33407.14	0.65
3.92	1392079	84188.97	1.64
4.23	414554	67519.27	1.31
4.34	5943024	261881.61	5.09
4.48	70377240	3206638.25	62.29
4.57	1394401	101512.40	1.97
4.88	827019	50408.95	0.98

3-(6-(4-nitrophenyl)-3-oxo-5-(*p*-tolyl)-3,4-dihydropyrazin-2-yl)propanamide (7i)

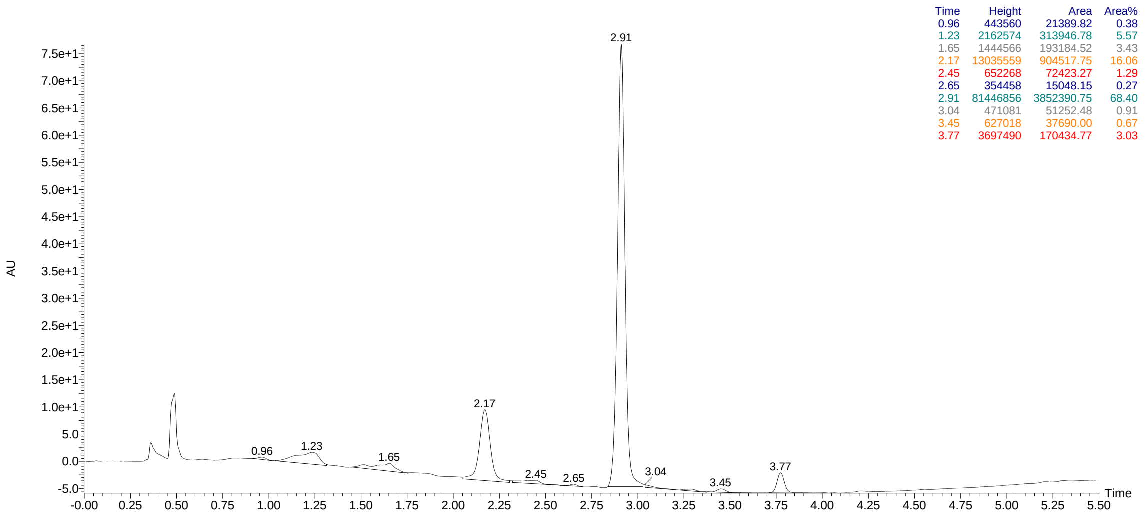

Time	Height	Area	Area%
0.96	443560	21389.82	0.38
1.23	2162574	313946.78	5.57
1.65	1444566	193184.52	3.43
2.17	13035559	904517.75	16.06
2.45	652268	72423.27	1.29
2.65	354458	15048.15	0.27
2.91	81446856	3852390.75	68.40
3.04	471081	51252.48	0.91
3.45	627018	37690.00	0.67
3.77	3697490	170434.77	3.03

1-(3-(6-(4-nitrophenyl)-3-oxo-5-(*p*-tolyl)-3,4-dihydropyrazin-2-yl)propyl)guanidine (**7j**)

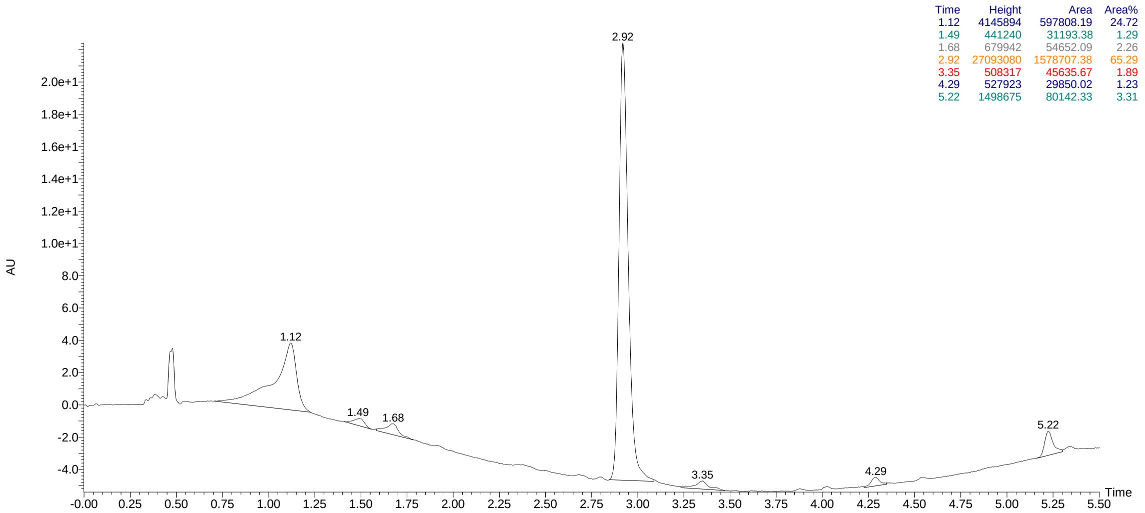

Time	Height	Area	Area%
1.12	4145894	597808.19	24.72
1.49	441240	31193.38	1.29
1.68	679942	54652.09	2.26
2.92	27093080	1578707.38	65.29
3.35	508317	45635.67	1.89
4.29	527923	29850.02	1.23
5.22	1498675	80142.33	3.31

6-(4-amino-3,5-dichlorophenyl)-3-methyl-5-(4-nitrophenyl)pyrazin-2(1*H*)-one (**7k**)

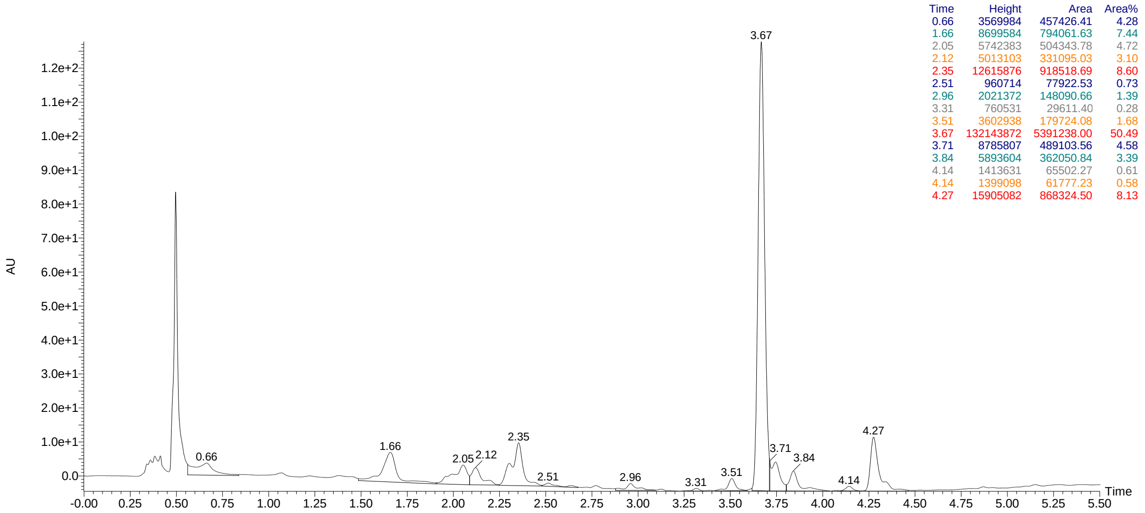

Time	Height	Area	Area%
0.66	3569984	457426.41	4.28
1.66	8699584	794061.63	7.44
2.05	5742383	504343.78	4.72
2.12	5013103	331095.03	3.10
2.35	12615876	918518.69	8.60
2.51	960714	77922.53	0.73
2.96	2021372	148090.66	1.39
3.31	760531	29611.40	0.28
3.51	3602938	179724.08	1.68
3.67	132143872	5391238.00	50.49
3.71	8785807	489103.56	4.58
3.84	5893604	362050.84	3.39
4.14	1413631	65502.27	0.61
4.14	1399098	61777.23	0.58
4.27	15905082	868324.50	8.13

3-((1*H*-indol-3-yl)methyl)-6-(4-methoxyphenyl)-5-(4-nitrophenyl)pyrazin-2(1*H*)-one (**7l**)

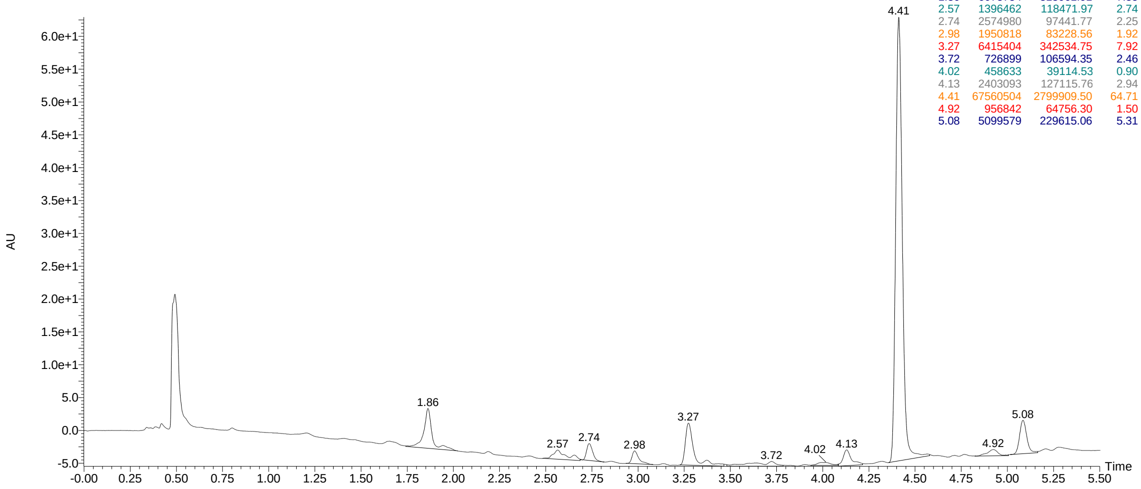

Time	Height	Area	Area%
1.86	6078754	318061.91	7.35
2.57	1396462	118471.97	2.74
2.74	2574980	97441.77	2.25
2.98	1950818	83228.56	1.92
3.27	6415404	342534.75	7.92
3.72	726899	106594.35	2.46
4.02	458633	39114.53	0.90
4.13	2403093	127115.76	2.94
4.41	67560504	2799909.50	64.71
4.92	956842	64756.30	1.50
5.08	5099579	229615.06	5.31
